# Nitro-fatty acids modulate germination onset through *S*-nitrosothiol metabolism

**DOI:** 10.1093/plphys/kiaf038

**Published:** 2025-01-25

**Authors:** Capilla Mata-Pérez, Juan C Begara-Morales, María N Padilla, Mounira Chaki, Beatriz Sánchez-Calvo, Alfonso Carreras, Lorena Aranda-Caño, Manuel Melguizo, Raquel Valderrama, Inmaculada Sánchez-Vicente, Óscar Lorenzo, Juan B Barroso

**Affiliations:** Group of Biochemistry and Cell Signaling in Nitric Oxide, Department of Experimental Biology, Faculty of Experimental Sciences, University Institute for Research in Olive Groves and Olive Oils, University of Jaén, Campus “Las Lagunillas” s/n, E-23071 Jaén, Spain; Faculty of Biology, Institute for Agribiotechnology Research (CIALE), University of Salamanca, E-37185 Salamanca, Spain; Group of Biochemistry and Cell Signaling in Nitric Oxide, Department of Experimental Biology, Faculty of Experimental Sciences, University Institute for Research in Olive Groves and Olive Oils, University of Jaén, Campus “Las Lagunillas” s/n, E-23071 Jaén, Spain; Group of Biochemistry and Cell Signaling in Nitric Oxide, Department of Experimental Biology, Faculty of Experimental Sciences, University Institute for Research in Olive Groves and Olive Oils, University of Jaén, Campus “Las Lagunillas” s/n, E-23071 Jaén, Spain; Group of Biochemistry and Cell Signaling in Nitric Oxide, Department of Experimental Biology, Faculty of Experimental Sciences, University Institute for Research in Olive Groves and Olive Oils, University of Jaén, Campus “Las Lagunillas” s/n, E-23071 Jaén, Spain; Department of Biochemistry and Biomedical Research Center (CEINBIO), School of Medicine, University of the Republic, 11800 Montevideo, Uruguay; Department of Basic Nutrition, School of Nutrition, University of the Republic, 11600 Montevideo, Uruguay; Group of Biochemistry and Cell Signaling in Nitric Oxide, Department of Experimental Biology, Faculty of Experimental Sciences, University Institute for Research in Olive Groves and Olive Oils, University of Jaén, Campus “Las Lagunillas” s/n, E-23071 Jaén, Spain; Group of Biochemistry and Cell Signaling in Nitric Oxide, Department of Experimental Biology, Faculty of Experimental Sciences, University Institute for Research in Olive Groves and Olive Oils, University of Jaén, Campus “Las Lagunillas” s/n, E-23071 Jaén, Spain; Department of Inorganic and Organic Chemistry, Faculty of Experimental Sciences, University of Jaén, Campus Universitario “Las Lagunillas” s/n, E-23071 Jaén, Spain; Group of Biochemistry and Cell Signaling in Nitric Oxide, Department of Experimental Biology, Faculty of Experimental Sciences, University Institute for Research in Olive Groves and Olive Oils, University of Jaén, Campus “Las Lagunillas” s/n, E-23071 Jaén, Spain; Faculty of Biology, Institute for Agribiotechnology Research (CIALE), University of Salamanca, E-37185 Salamanca, Spain; Faculty of Biology, Institute for Agribiotechnology Research (CIALE), University of Salamanca, E-37185 Salamanca, Spain; Group of Biochemistry and Cell Signaling in Nitric Oxide, Department of Experimental Biology, Faculty of Experimental Sciences, University Institute for Research in Olive Groves and Olive Oils, University of Jaén, Campus “Las Lagunillas” s/n, E-23071 Jaén, Spain

## Abstract

Nitro-fatty acids (NO_2_-FAs) have emerged as key components of nitric oxide (NO) signaling in eukaryotes. We previously described how nitro-linolenic acid (NO_2_-Ln), the major NO_2_-FA detected in plants, regulates *S*-nitrosoglutathione (GSNO) levels in Arabidopsis (*Arabidopsis thaliana*). However, the underlying molecular mechanisms remain undefined. Here, we used a combination of physiological, biochemical, and molecular approaches to provide evidence that NO_2_-Ln modulates *S*-nitrosothiol (SNO) content through *S*-nitrosylation of *S*-nitrosoglutathione reductase1 (GSNOR1) and its impact on germination onset. The *aer* mutant (a knockout mutant of the alkenal reductase enzyme; AER) exhibits higher NO_2_-Ln content and lower *GSNOR1* transcript levels, reflected by higher SNO content and *S*-nitrosylated proteins. Given its capacity to release NO, NO_2_-Ln mediates the *S*-nitrosylation of GSNOR1, demonstrating that NO_2_-FAs can indirectly modulate total SNO content in plants. Moreover, the ectopic application of NO_2_-Ln to dormant seeds enhances germination success similarly to the *aer* germination rate, which is mediated by the degradation of master regulator ABSCISIC ACID INSENSITIVE 5 (ABI5). Our results establish that NO_2_-FAs regulate plant development through NO and SNO metabolism and reveal a role of NO_2_-FAs in plant physiology.

## Teaser

Nitrated fatty acids regulate plant development through nitric oxide and *S*-nitrosothiol metabolism.

## Introduction

In recent years, research into the understanding of the nitric oxide (NO) function in plant systems has substantially grown. NO was initially found to be a modulator of plant defense during plant–pathogen interactions (Delledonne et al. 1998; Durner et al. 1998). More attention has been paid to this molecule due to its relation to diverse physiological processes, i.e. breaking seed dormancy and stimulating germination (Beligni and Lamattina 2000; Albertos et al. 2015), senescence (Begara-Morales et al. 2013), or stomatal movement (García-Mata and Lamattina 2001; Garcia-Mata and Lamattina 2007). In addition, NO has been related to response to several (a)biotic stresses, i.e. drought, salinity, cold, heat, or pathogen infection (Feechan et al. 2005; Valderrama et al. 2007; Chaki et al. 2009a, 2011b, 2011a, 2013; Lindermayr et al. 2010; Begara-Morales et al. 2014).

*S*-Nitrosoglutathione (GSNO), a potent NO donor and cellular reservoir for NO bioactivity, results from the interaction of NO and reduced glutathione (GSH) through a reaction called *S*-nitrosylation. This NO-derived posttranslational modification (NO-PTM) consists of the reversible binding of an NO moiety to a reactive thiol group (-SH) to form a protein-SNO or *S*-nitrosothiol (SNO). *S*-Nitrosylation is a well-established ubiquitous PTM in plant biology, which leads to alterations in protein stability, activity, conformation, and localization (Lindermayr et al. 2005; Romero-Puertas et al. 2008; Camejo et al. 2013; Begara-Morales et al. 2018, 2019). Protein-SNO can also transfer NO moiety to the sulfhydryl (-SH) group of other proteins by a process specifically termed *S*-transnitrosylation. NO can also react with superoxide anion (O_2_·^−^) to yield powerful oxidant peroxynitrite (ONOO^−^), which can modify tyrosine residues by adding a nitro group (-NO_2_) in an irreversible reaction known as protein tyrosine nitration (Radi 2012).

Unlike most other PTMs, SNO formation is not directly facilitated by enzymes. Nonetheless, a *S*-nitrosoglutathione reductase1 (GSNOR1) enzyme has been identified that controls GSNO levels by breaking it down into oxidized GSH and ammonium. The knockout of this enzyme results in high GSNO and, consequently, protein-SNO levels that disable the plant defense responses conferred by distinct resistance genes (Feechan et al. 2005). This indicates that GSNOR1 indirectly governs the protein-SNO level. GSNOR1 is also regulated by *S*-nitrosylation to provide NO with feedback control over its own signaling pathway (Frungillo et al. 2014). During hypoxia responses, *S*-nitrosylation at Cys10 induces the selective autophagy of GSNOR1 by establishing a molecular link between NO signaling and autophagy in Arabidopsis (*Arabidopsis thaliana*) (Zhan et al. 2018). Conversely, other studies demonstrate that higher SNO content due to GSNOR1 mutation leads to enhanced basal resistance against *Peronospora parasitica* (Rustérucci et al. 2007) or *Plasmopara halstedii* (Chaki et al. 2009a). High SNO levels have been described in abiotic stresses, including light, darkness, salinity, high and low temperature, and wounding stresses in several plant species (Valderrama et al. 2007; Corpas et al. 2008; Chaki et al. 2011b, 2011a; Begara-Morales et al. 2019). All this demonstrates the implication of SNO metabolism in plants' response to (a)biotic stressful situations.

Although studies on the functional role of NO in plants have focused mainly on NO-PTM of proteins by nitration and *S*-nitrosylation, research into the interaction of NO and reactive nitrogen species (RNS) with other biomolecules, such as electrophilic fatty acids (FAs), is currently growing. Indeed, the interaction of unsaturated FAs and NO-derived species leads to nitro-FA (NO_2_-FA) formation. These NO_2_-FAs have emerged in recent years as molecules that perform a relevant function in plants. In fact, the presence of nitro-linolenic acid (NO_2_-Ln) has been observed in Arabidopsis and in relevant crops like rice, pea, or lettuce (Mata-Pérez et al. 2016c, 2017).

NO_2_-FAs have recently been described as key signaling molecules during plant development and in the defense response against abiotic stress conditions through the induction of heat shock transcription factors (TFs) that regulate the expression of antioxidant systems (recently reviewed in Gupta et al. 2020; Begara-Morales et al. 2021; Di Fino et al. 2021). More recently, the unknown storage biomolecules of endogenous NO_2_-FAs, such as NO_2_-Ln, nitro-oleic acid (NO_2_-OA), and nitro-linoleic acid (NO_2_-LA), have been identified in *A. thaliana*. The distribution of nitrated FAs in storage biomolecules during plant development has been determined. Phytosterol esters (SEs) and triacylglycerols (TAGs) are reservoir biomolecules in seeds that are replaced with phospholipids and proteins in vegetative, generative, and senescence stages (Aranda-Caño et al. 2022a). The above results show that NO_2_-Ln, NO_2_-LA, and NO_2_-OA levels esterified in both lipid and protein storage biomolecules with a decreasing pattern throughout Arabidopsis development (Aranda-Caño et al. 2022a). Thus, analyzing NO_2_-Ln content throughout plant life evidences the highest content upon plant growth onset, specifically in seeds (Mata-Pérez et al. 2016c; Vollár et al. 2020). This finding suggests the putative implication of NO_2_-FAs in early plant development. In relation to its extreme abundance in seeds, recent findings provide insight into the molecular framework of the role of NO_2_-Ln in lipid accumulation during embryonic development. NO release from NO_2_-Ln accumulates in developing embryos and regulates the FA profile by basic/leucine zipper TF bZIP67 stabilization. This scenario provides compelling evidence for the in vivo functionality of NO_2_-FAs during plant developmental signaling (Sánchez-Vicente et al. 2024). Furthermore, the esterification of NO_2_-FAs in phospholipids and proteins reinforces their involvement in both biomembrane dynamics and signaling processes, respectively (Aranda-Caño et al. 2022a). Further detection of increased NO_2_-Ln levels according to several abiotic stressful traits suggests that NO_2_-Ln is able to elicit a defense response against cellular stress by inducing several antioxidant systems, such as ascorbate peroxidase (APX) and many heat shock proteins (HSPs) (Mata-Pérez et al. 2016c).

Despite these relevant NO_2_-FA properties, the mechanisms by which these molecules are able to launch defense responses remain unknown. One of the biological properties of NO_2_-FA is its ability to release NO in aqueous physiological environments (Schopfer et al. 2005; Gorczynski et al. 2007). This capacity has been described for NO_2_-LA and NO_2_-OA in animals (Lima et al. 2005; Schopfer et al. 2005; Gorczynski et al. 2007) and for NO_2_-Ln in plant systems (Mata-Pérez et al. 2016a). NO released from NO_2_-FA can mediate *S*-nitrosylation processes, as in the case of *S*-nitrosylation of proinflammatory member CD40 by triggering an antiinflammatory response (Faine et al. 2010). NO_2_-FA's own electrophilic properties allow it to interact with nucleophiles of biological systems, such as the thiol groups of cysteines (Geisler and Rudolph 2012). This reversible interaction of electrophilic NO_2_-FA and nucleophiles is specifically termed nitroalkylation. It is an important, widely described signaling mechanism in animals (Cui et al. 2006; Schopfer et al. 2010, 2011) and has been recently noted in yeast and plant systems (Aranda-Caño et al. 2019, 2022b).

NO_2_-FAs' capacity to perform important cellular signaling functions *via* NO release has been recently described for NO_2_-Ln. NO from this NO_2_-FA reacts with GSH to synthesize GSNO and is able to modulate GSNO levels in vitro and in vivo in Arabidopsis plants (Mata-Pérez et al. 2020). In parallel, the enzyme *alkenal reductase* (AER) has been suggested as a nitroalkene reductase that specifically affects NO_2_-Ln content. Thus, mutants deficient of this enzyme (*aer*) display significantly higher NO_2_-Ln and GSNO contents (Mata-Pérez et al. 2020), which highlights the role of NO_2_-Ln in modulating GSNO metabolism in plants. These results motivated us to further explore NO_2_-Ln's capacity to regulate SNO metabolism. We herein demonstrate that NO_2_-Ln mediates the *S*-nitrosylation of enzyme GSNOR1 given its capability to release NO, which leads to an increase in the protein-SNO involved in a myriad of physiological and stress-related processes. Finally, we evidence a role for NO_2_-Ln in seed dormancy break via the *S*-nitrosylation of the central hub for growth repression *ABSCISIC ACID INSENSITIVE 5* (ABI5), which underlines the relevance of NO signaling mediated by NO_2_-FAs in plants.

## Results

### NO_2_-Ln modulates SNO content

**Figure 1.**
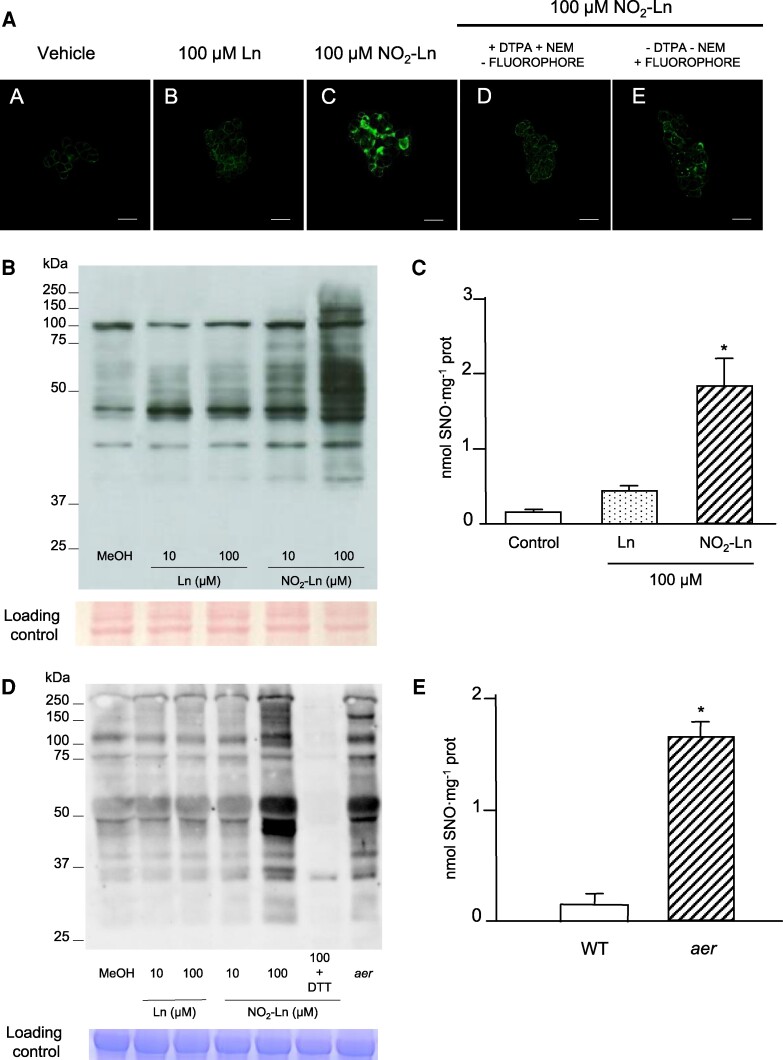
NO_2_-Ln modulates SNO content. **A)** Confocal laser scanning microscopy in vivo detection of SNO. The assay was performed using Alexa Fluor (AF) 488 Hg-link reagent as the fluorescent probe for SNO (in green). ACSCs were preincubated with methanol (vehicle) (A), 100 *µ*m (Ln) (B), or 100 *µ*m NO_2_-Ln (C). SNO signal specificity was demonstrated with different negative controls, including the incubation with and without DTPA and NEM, or with and without the fluorophore (D, E). Bars indicate 50 *µ*m. **B)** NO_2_-Ln induced *S*-nitrosylation in ACSCs. Cell suspension cultures were exposed to NO_2_-Ln and subjected to the BST. Cell cultures were incubated with methanol, 10 and 100 *μ*m Ln, and with 10 and 100 *μ*m NO_2_-Ln. Ponceau staining ensures equal protein loading (see Supplementary Fig. S1B). Molecular weight markers are highlighted on the left side of the immunoblot. **C)** Total SNO content was enhanced by NO_2_-Ln. ACSCs were incubated with methanol, 100 *µ*m Ln or NO_2_-Ln. Total SNO was detected by ozone chemiluminescence. **D)** Plant protein extracts were *S*-nitrosylated with NO_2_-Ln, and protein-SNO was analyzed and detected as in **B)**. SNO break induced by dithiothreitol (DTT) served as a control. Coomassie staining ensured equal protein loading (see Supplementary Fig. S1C). Molecular weight markers are highlighted on the left side of the immunoblot. **E)** Endogenous levels of SNO in the WT and *aer* seedlings analyzed as in **C)**. The error bars in **C)** and **E)** represent SEM (*n* = 3). Asterisks indicate significant differences from controls (Student's *t* test, *P* < 0.05).

There has been recent evidence for NO_2_-Ln being able to modulate GSNO content in Arabidopsis (Mata-Pérez et al. 2020). To examine whether this NO_2_-FA can modulate total SNO levels, we used Arabidopsis cell cultures because we initially evidenced these molecules' signaling effect in this plant system (Mata-Pérez et al. 2016c). Confocal microscopy analysis after incubating cultures with 100 *µ*m NO_2_-Ln prompted intense green fluorescence due specifically to NO_2_-Ln treatment compared to endogenous SNO and the corresponding controls (Fig. 1A). This result allowed us to further comprehend whether this increase in total SNO, together with NO_2_-FAs' ability to release NO (Lim et al. 2002; Baker et al. 2005; Mata-Pérez et al. 2016a), could impact NO-PTMs. Protein tyrosine nitration was firstly analyzed by immunoblot against 3-nitrotyrosine (NO_2_-Tyr) in cultures incubated with 10 and 100 *µ*m NO_2_-Ln (Supplementary Fig. S1, A and B). The results showed no change in protein tyrosine nitration abundance, which suggests that NO_2_-Ln does not modulate this NO-PTM. By the biotin switch technique (BST), we observed that NO_2_-Ln prompted a dose-dependent increase in protein-SNO, which was more noticeable when employing 100 *µ*m NO_2_-Ln (Fig. 1B). NO_2_-FAs lie in cells at low concentrations and range from picomolar to nanomolar in animals (Tsikas et al. 2009) and plants (Mata-Pérez et al. 2016a; Aranda-Caño et al. 2022a, 2022b). However, most studies in this field have used higher concentrations of these nitrated derivatives to evidence physiological implications in biological systems (Kansanen et al. 2009; Mata-Pérez et al. 2016a). In line with previous similar approaches, we selected 100 *µ*m of NO_2_-Ln as a standard concentration for all the assays, which herein showed evidence for a clear and solid response due to this NO_2_-FA.

These results motivated further SNO modulation characterization by NO_2_-Ln. Ozone chemiluminescence was used to measure total SNO levels. NO_2_-Ln significantly enhanced total SNO abundance (1.83 nmol mg^−1^ protein) (12-fold) compared to the endogenous content observed in the untreated and Ln-treated (respectively 0.15 and 0.42 nmol mg^−1^ protein) cell cultures (Fig. 1C). NO_2_-Ln can apparently modulate SNO content in Arabidopsis. These results are closely related to the recently proven modulation exerted by this NO_2_-FA on GSNO content (Mata-Pérez et al. 2020).

When seeking genetic evidence to support these data, we used an *aer-*deficient mutant line because it is reported to endogenously contain higher NO_2_-Ln levels (Mata-Pérez et al. 2020). Moving from cell cultures to Arabidopsis plants, we performed a BST using Col-0 and *aer* mutant seedlings. The total protein extracts from Col-0 were incubated with 10 and 100 *µ*m of Ln or NO_2_-Ln, or were left untreated. As Fig. 1D depicts, both 100 *µ*m NO_2_-Ln and the *aer* conditions brought about a similar prominent increase in protein-SNO abundance compared to the controls. The DTT treatment resulted in barely any SNO signal, which highlights NO_2_-Ln's specific capacity to induce protein-SNO. As described for GSNO, the endogenous total SNO levels were measured by ozone chemiluminescence in the *aer* mutant. As expected and in accordance with the higher GSNO levels found in this mutant (Mata-Pérez et al. 2020), total SNO content was 11-fold higher vs. the wild-type (WT) seedlings (Fig. 1E). Overall, the data presented herein evidence NO_2_-Ln's implication in SNO metabolism in Arabidopsis.

### GSNOR1 is a target of NO_2_-Ln

**Figure 2.**
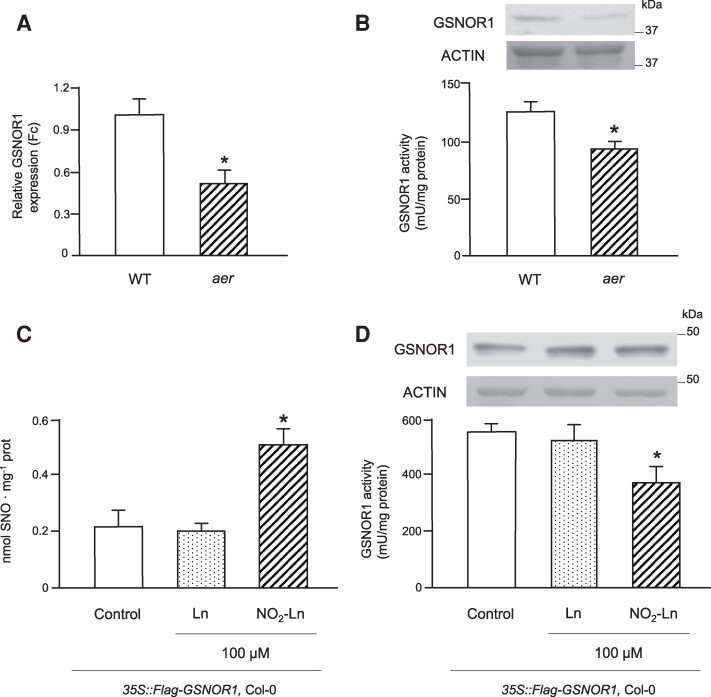
GSNOR1 is a target of NO_2_-Ln. **A)***GSNOR1* expression decreased in the *aer* mutant. *GSNOR1* transcript abundance was analyzed by real-time quantitative PCR in the WT and *aer* seedlings. *18S* and *L2* RNA served as internal controls. **B)** The protein abundance and enzymatic activity of GSNOR1 in the WT and *aer* seedlings. GSNOR1 was detected using an anti-GSNOR1 antibody. GSNOR1 enzymatic activity in the WT seedlings was 130 mU mg^−1^ protein. Actin was used as the loading control. The position of a 37 kDa marker is indicated. **C)** NO_2_-Ln induces SNO formation in the *35S:Flag-GSNOR1* construction in the WT. Seedlings were treated with 100 *µ*m NO_2_-Ln, and total SNO was analyzed by ozone chemiluminescence. The H_2_O and 100 *µ*m Ln treatments showed endogenous SNO content. **D)** GSNOR1 enzyme activity was affected by the NO_2_-Ln treatment. Both GSNOR1 protein level and enzymatic activity were studied in the *35S:Flag-GSNOR1* construction in the WT after incubation with 100 *µ*m NO_2_-Ln. The H_2_O and 100 *µ*m Ln treatments served as controls. GSNOR1 activity in the control seedlings was 550 mU mg^−1^ protein. Actin was used as the loading control. The position of a 50 kDa marker is indicated. In all graphs, error bars represent SEM (*n* = 3). Asterisks indicate significant differences from controls (Student's *t* test, *P* < 0.05).

In order to understand how NO_2_-Ln modulates SNO levels, we analyzed the transcriptional and translational GSNOR1 abundance. The *aer*-deficient mutant showed a 50% decrease in the *GSNOR1* transcript levels compared to the WT seedlings (Fig. 2A). This reduction was concomitant with the lesser abundance of GSNOR1 protein content (Fig. 2B), which evidences that NO_2_-Ln directly affects GSNOR1 bioactivity and therefore modifies SNO metabolism.

Given these results, we looked for a strategy in which GSNOR1 is constitutively expressed. We incubated *35S:Flag-GSNOR1* with 100 *µ*m Ln or NO_2_-Ln and measured total SNO content by ozone chemiluminescence. As presented in Fig. 2C, NO_2_-Ln prompted a 61% rise in SNO content compared to the Ln and untreated controls. This behavior was accompanied by a 35% drop in GSNOR1 enzymatic activity after incubating with NO_2_-Ln. No affect was observed under the control conditions (Fig. 2D). To determine whether this NO_2_-FA could affect protein stability, we further tested GSNOR1 protein abundance after the NO_2_-Ln treatment. No changes took place in any of the analyzed conditions (Fig. 2D). The herein presented data support evidence that NO_2_-Ln modulates GSNOR1 bioactivity mainly at the posttranslational level, which affects total SNO content in plants.

### NO_2_-Ln mediates the *S*-nitrosylation of GSNOR1

**Figure 3.**
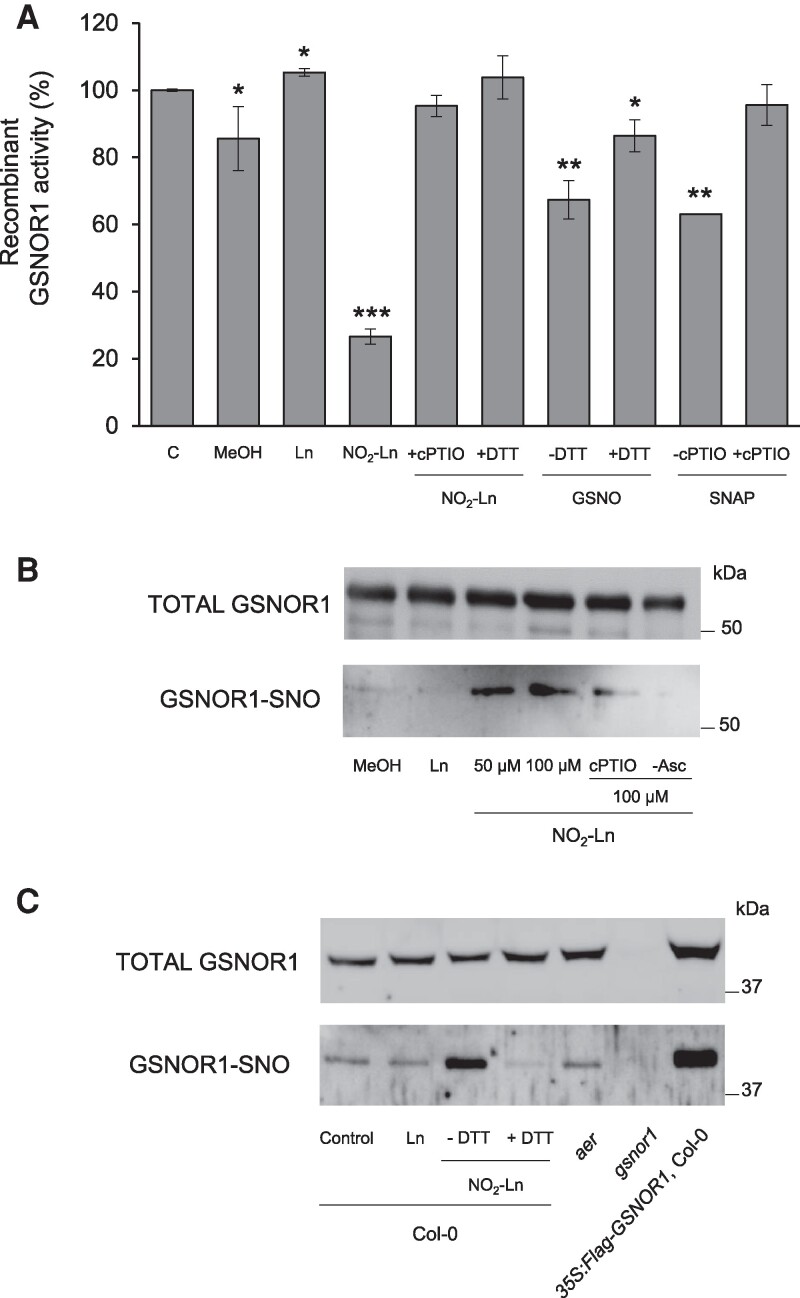
NO released from NO_2_-Ln mediates the GSNOR1-SNO. **A)** Modification of Arabidopsis GSNOR1 with reactive nitrogen donors inhibited enzyme activity. Arabidopsis recombinant GSNOR1 (Lytag-GSNOR1) was incubated with 100 *µ*m Ln, NO_2_-Ln, GSNO, or SNAP in the presence or absence of 500 *µ*m cPTIO or 5 mm DTT. GSNOR1 enzymatic activity was 3.5 *µ*moL NADH min^−1^ mg^−1^ protein under the control condition. Error bars represent SEM (*n* = 3). Asterisks indicate significant differences from the untreated **C)** condition (Student's *t* test, *P < 0.05, **P < 0.01, ***P < 0.001). **B)** Arabidopsis recombinant GSNOR1 (Lytag-GSNOR1) was incubated in the presence of methanol, 100 *µ*m Ln; 50 and 100 *µ*m NO_2_-Ln; 100 *µ*m NO_2_-Ln in combination with 200 *µ*m cPTIO; and 100 *µ*m NO_2_-Ln without ascorbate (used as the negative control). GSNOR1-SNO was detected by the BST and is shown in relation to total GSNOR1. The position of a 50 kDa marker is indicated. **C)** Protein extracts from Arabidopsis seedlings were *S*-nitrosylated with 100 *µ*m NO_2_-Ln. GSNOR1-SNO was detected by the BST and is shown in relation to total GSNOR1. DTT-induced SNO break and *gsnor1* served as the negative controls. The position of a 37 kDa marker is indicated.

The previously presented data indicated the posttranslational regulation of GSNOR1 by NO_2_-Ln. We firstly carried out in vitro characterization of the enzymatic activity of Arabidopsis recombinant GSNOR1 in the presence of several NO donors (i.e. GSNO, SNAP, and NO_2_-Ln). GSNOR1 activity was significantly reduced in all the NO treatments and accounted for an approximately 30% drop after adding GSNO and SNAP. GSNOR1 bioactivity inhibition was 70% when 100 *µ*m NO_2_-Ln was applied (Fig. 3A). To decipher whether this inhibition was due to an NO-dependent mechanism, GSNOR1 was concomitantly assayed in the presence of a NO scavenger (i.e. cPTIO or reductant-like DTT). As Fig. 3A depicts, both treatments allowed enzymatic activity to be restored back to the control levels. These findings generally highlight that GSNOR1 bioactivity is affected by NO_2_-Ln through a NO-dependent mechanism. These results agree with data published after testing other NO donors (Frungillo et al. 2014; Guerra et al. 2016). Our obtained results prompted us to perform an in vitro BST assay. It is evidenced that NO_2_-Ln can mediate the *S*-nitrosylation of GSNOR1 (GSNOR1-SNO) in a NO-dependent manner, as observed when both NO_2_-Ln and cPTIO were concomitantly applied to the GSNOR1 recombinant protein (Fig. 3B).

From a more physiological view, we used protein extracts from the Col-0 seedlings incubated with 100 *µ*m Ln or NO_2_-Ln, and other genetic backgrounds like *aer*, *gsnor1*, and 35S:Flag-GSNOR1, to explore the GSNOR1-SNO by NO_2_-Ln. The results evidenced that this NO_2_-FA mediated an increase in GSNOR1-SNO abundance compared to the Ln- and untreated conditions. This signal was specific when adding reductant DTT (SNO break) and confirmed the results obtained with recombinant GSNOR1 (Fig. 3C). Under our experimental conditions, we observed a slight increase in the *S*-nitrosylation of the endogenous GSNOR1 in the *aer* background, and no signal was detected in the *gsnor1* mutant. Finally, as expected, *35S:Flag-GSNOR1* exhibited larger amounts of total GSNOR1 and GSNOR1-SNO (Fig. 3C).

It is well established that NO modifies protein structure and function mainly through PTMs like *S*-nitrosylation. Almost 1 decade ago, Frungillo et al. (2014) well described how GSNOR1 can be regulated by nitrated-derived NO through *S*-nitrosylation, which leads to reduced protein activity. Other plant GSNOR enzymes, such as tomato (*Solanum lycopersicum*), cauliflower (*Brassica oleracea* var. *botrytis*), and lettuce (*Lactuca sativa*), or human and yeast (*Saccharomyces cerevisiae*), are susceptible to this PTM (Guerra et al. 2016; Tichá et al. 2017). Hence, these findings define NO_2_-Ln's capacity to modulate cellular SNO levels by inhibiting GSNOR1 activity via *S*-nitrosylation.

### NO_2_-Ln modulates germination onset

**Figure 4.**
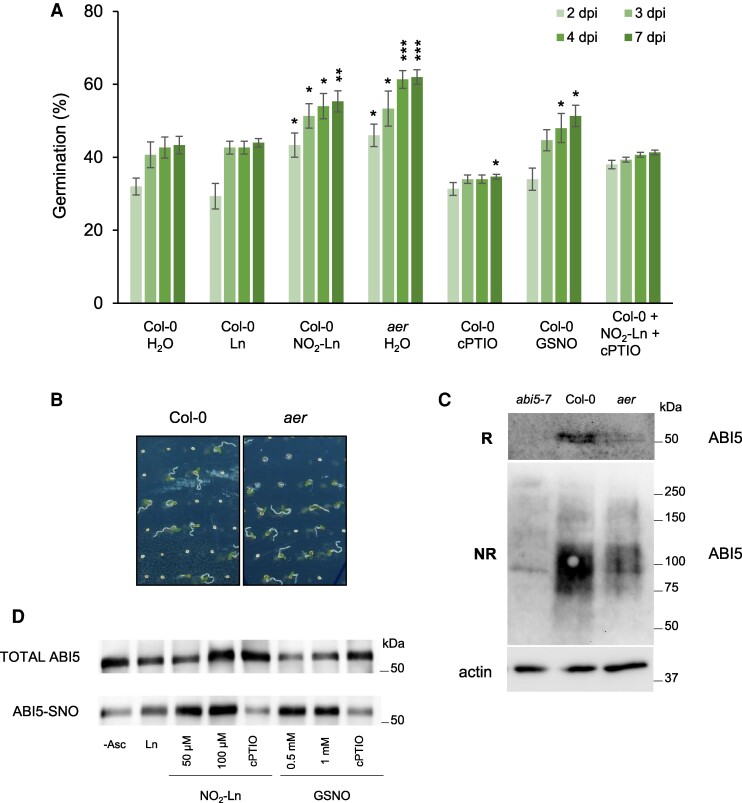
NO_2_-Ln modulates germination onset. **A)** Bar plot for the germination of the WT (Col-0) and *aer* seeds in media containing 100 *µ*m Ln, 100 *µ*m NO_2_-Ln, 1 mm GSNO, a combination of 100 *µ*m NO_2_-Ln with 300 *µ*m cPTIO, or 300 *µ*m cPTIO alone. The percentage of seeds with an emerged radicle (germination) was determined 2, 3, 4, and 7 d after sowing (dpi). Each value represents the average germination percentage of 60 seeds with the Se of 3 replicates. Experiments were run 3 times. Representative behavior obtained from 3 approaches is shown. Dunnett's test was performed to determine statistical differences against the Col-0 control seeds (*P < 0.05, **P < 0.01, ***P < 0.001). **B)** Representative pictures of the different treatments performed as described in **A)**. Pictures were taken 4 d post-imbibition (4 dpi). A portion of the Petri plate showing the behavior of every condition is depicted. **C)** Immunoblot analysis of the ABI5 protein levels in the seed extracts of the Col-0 and *aer* seeds. Seeds were vernalized for 48 h at 4 °C, sown on MES plates and collected 48 h after sowing. The upper panel depicts western blotting under reducing (R) conditions. The bottom panel shows it under nonreducing (NR) conditions. Actin protein levels are revealed as a loading control. *abi5-7*: the loss-of-function mutant of ABI5. Molecular weight markers are highlighted on the right side of the immunoblot. **D)***S*-Nitrosylation assay of recombinant ABI5. The Arabidopsis ABI5 recombinant protein was subjected to the BST and incubated in the presence of 100 *µ*m NO_2_-Ln without ascorbate (used as the negative control), 100 *µ*m Ln, 50 and 100 *µ*m NO_2_-Ln, 100 *µ*m NO_2_-Ln in combination with 200 *µ*m cPTIO, 500 *µ*m and 1 mm GSNO, and 1 mm GSNO in combination with 1 mm cPTIO. The total ABI5 protein ensures equal protein loading. The position of a 50 kDa marker is indicated. -Asc, without ascorbate.

As previously described, the highest NO_2_-Ln levels were detected in Arabidopsis seeds with almost 3-fold the abundance found in the 14-day-old seedlings (Mata-Pérez et al. 2016c). Plant survival depends on seed germination and progression through postgerminative developmental checkpoints. Considerable research has demonstrated that NO impacts seed dormancy and germination by either the exogenous application of NO donors or genetic evidence on mutants with disrupted endogenous levels in Arabidopsis (Beligni and Lamattina 2000; Lozano-Juste and León 2010). These results encouraged us to look more closely at NO_2_-Ln's involvement in early plant life stages. To do so, we treated after-ripened Col-0 seeds with this NO_2_-FA to determine its impact on seed germination. NO_2_-Ln application caused a significant dose-dependent increase in germination success compared to the control and Ln-treated seeds (Fig. 4A; Supplementary Fig. S2A). Conversely, endogenous NO depletion by the cPTIO scavenger, either alone or in combination with NO_2_-Ln, inhibited seed germination. Pharmacological treatments that include known NO donors like GSNO are typically applied at nonphysiological concentrations (Ferrarini et al. 2008; Begara-Morales et al. 2014; Albertos et al. 2015). Unlike other NO donors, such as sodium nitroprusside (SNP), how NO_2_-Ln releases NO to media resembles GSNO kinetics (Mata-Pérez et al. 2016b). Accordingly, we observed that NO_2_-Ln promoted seed germination similarly to when GSNO was applied to seeds (Supplementary Fig. S2A). More interestingly, the germination rates found in the *aer* mutant (detail shown on Fig. 4, A and B) were analogous to those noted in the seeds incubated with NO_2_-Ln (Fig. 4A; Supplementary Fig. S2A). These results highlight similar in vitro and in vivo dynamics and reinforce the idea that this NO_2_-FA plays a role in seed dormancy breakage. Furthermore, the higher germination observed in the *aer* mutant was further increased by the application of 100 *µ*m NO_2_-Ln and 1 mm GSNO. This confirms that this NO_2_-FA does not saturate intracellular SNO content (Supplementary Fig. S2B).

Abscisic acid (ABA) is a stress phytohormone that inhibits seed germination and prompts early seedling development arrest (Lopez-Molina et al. 2001; Nambara et al. 2010). Such capacity is based on basic leucine zipper-type (bZIP) TF *ABI5*. Decisions on promoting or not germination rely mainly on the equilibrium between ABI5 stability and NO. It has been shown that the *S*-nitrosylation of this TF leads to its proteasome-dependent degradation and, therefore, to seed germination (Gibbs et al. 2014; Albertos et al. 2015). Considering NO_2_-Ln's implication in promoting germination onset, we studied ABI5 protein abundance in both Col-0 and the *aer* mutant background. The analysis of *S*-nitrosylation on ABI5 was performed on its functional homodimeric structure under nonreducing conditions (Nakamura et al. 2001; Albertos et al. 2015). ABI5 levels drastically lowered in the *aer* mutant-deficient line compared to the Col-0 seeds (Fig. 4C). These findings suggest that NO_2_-Ln might stimulate seed germination through the degradation of ABI5, a central hub for growth repression. As previously mentioned, NO targets ABI5 to proteasome-dependent degradation through *S*-nitrosylation (Albertos et al. 2015). We tested in vitro NO_2_-Ln's capacity to mediate the *S*-nitrosylation of ABI5 (ABI5-SNO). As seen in Fig. 4D, both NO_2_-Ln and GSNO were able to promote this PTM compared to the controls. The -SNO signal drastically reduced when NO_2_-Ln was applied in combination with cPTIO. This highlights that this NO_2_-FA mediates a NO-dependent PTM of this TF. As described, *S*-nitrosylation of ABI5 relies mainly on Cys153 (Albertos et al. 2015). By using the mutated version of the ABI5 recombinant protein where Cys153 was mutated to Ser (*Cys153Ser*), we found that NO_2_-Ln and GSNO were unable to promote ABI5-SNO. This finding suggests that both NO donors target Cys153 for *S*-nitrosylation in the ABI5 structure (Supplementary Fig. S2C). Together, these findings highlight that NO_2_-FAs, and specifically NO_2_-Ln, might promote seed dormancy break by being able to release NO which, in turn, mediates the *S*-nitrosylation of ABI5 by possibly targeting it for proteasome-dependent degradation.

### Identification of protein-SNO mediated by NO_2_-Ln

NO_2_-FAs can modulate protein activity through different PTMs, which highlights *S*-nitrosylation and nitroalkylation (Cui et al. 2006; Faine et al. 2010; Schopfer et al. 2010, 2011; Geisler and Rudolph 2012). The latter involves the adduction of NO_2_-FAs to protein nucleophiles, such as His, Cys, or Lys residues, in a NO-independent mechanism. This has been shown to occur in animal, yeast, and plant systems (Aranda-Caño et al. 2019, 2022b). Based on NO_2_-FAs' previously described capacity to release NO and the close connection to protein *S*-nitrosylation events in eukaryotes, the total protein extracts from the Arabidopsis cell suspension cultures (ACSCs) treated with methanol, 100 *µ*m Ln, or 100 *µ*m NO_2_-Ln were subjected to BST and MS (see Materials and methods) to identify the protein-SNO targets mediated by NO_2_-Ln.

In response to this NO_2_-FA, 1,520 proteins were detected and at least 1 unique peptide was identified. Irrespective of its length, a unique peptide is defined as a peptide that exists only in one protein of a proteome of interest, even though this peptide may appear more than once in the same protein. According to this definition, we restricted the list of protein candidates to the identification of 2 unique peptides. This analysis resulted in the presence of 593 proteins (Supplementary Table S1).

**Figure 5.**
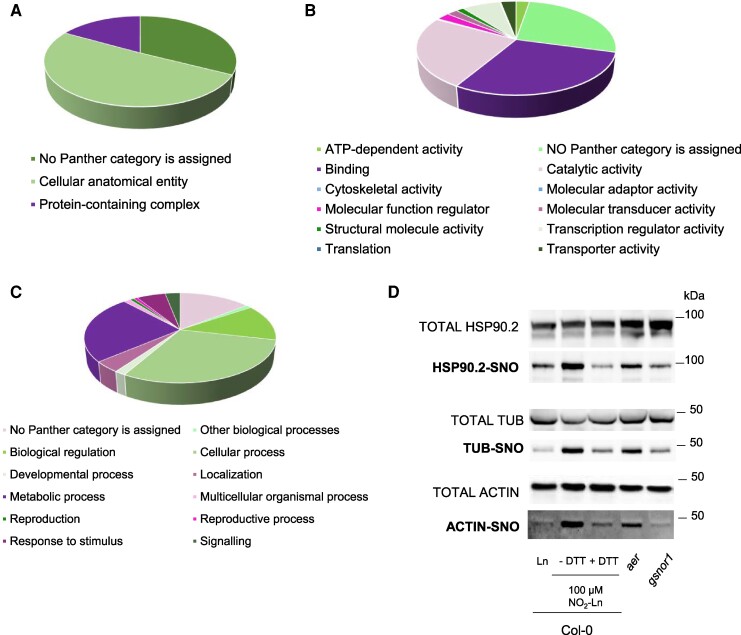
The GO analysis protein-SNO mediated by NO_2_-Ln. The *S*-nitrosylated proteins identified by MS approaches after incubating ACSCs with 100 *µ*M NO_2_-Ln were analyzed by the Panther software. GO categories are depicted as **A)** cellular component, **B)** molecular function, and **C)** biological processes. **D)** The protein extracts from Arabidopsis seedlings were *S*-nitrosylated with 100 *µ*m Ln or NO_2_-Ln. Protein-SNO was detected by the BST and shown in relation to totals. Both mutants *aer* and *gsnor1* were included to show the endogenous *S*-nitrosylation status of the selected proteins in these 2 genetic backgrounds. The DTT-induced SNO break served as the negative control. Molecular weight markers are highlighted on the right side of immunoblots.

Proteins were classified with functional Gene Ontology (GO) terms (Fig. 5). This analysis showed that most protein-SNOs were included in the GO term cellular anatomical entity, followed by a protein-containing complex term (Fig. 5A). An in-depth detail evidenced that NO_2_-Ln prompts the *S*-nitrosylation of diverse proteins that are widely located within the cell, and mainly in the cytoplasm, cytosol, nucleus, membrane, and organelles (Supplementary Fig. S3A). Interestingly, a portion of the protein-SNO modulated by NO_2_-Ln formed part of catalytic, membrane, proteasome, and ribonucleoprotein complexes (Supplementary Fig. S3B).

For molecular functions, binding, catalytic, and transcription regulator activities were overrepresented (Fig. 5B). These data suggest that NO_2_-Ln targets *S*-nitrosylation proteins and has the capacity to bind mostly organic cyclic or heterocyclic compounds, ions, and other proteins (Supplementary Fig. S3C). Of the protein-SNO with enzymatic activity, the hydrolase and oxidoreductase activities (Supplementary Fig. S3D) depicted by NADP-malic enzyme 2 (NADP-ME2), diverse peroxiredoxins (PRXIIB, PRXIIC, and PRXIIF), or ascorbate peroxidase 1 (APX1), among others, are highlighted. Interestingly, several of these antioxidant systems, including Arabidopsis recombinant APX and the peroxiredoxin Tsa1 from *S. cerevisiae*, are well modulated by NO_2_-FAs by protein nitroalkylation and, thus, impact their catalytic activities (Aranda-Caño et al. 2019, 2022a, 2022b). These findings generally show that NO_2_-Ln can modulate the bioactivity of several antioxidant systems (i.e. APX) via the interplay between 2 distinct NO-mediated PTMs.

Of the main biological processes modulated by NO_2_-Ln, we stress cellular and metabolic processes, the response to stimuli, and development (Fig. 5C). Closely related to these aspects is protein class categorization, which underlines the marked representation of metabolic interconversion enzymes, translational proteins, and, interestingly, chaperones (Supplementary Fig. S3E). When looking in detail at the protein-SNO list mediated by NO_2_-Ln, we saw that almost 20 proteins belonged to the HSP family (Supplementary Table S1).

Based on NO_2_-Ln's capacity to release NO and to intervene in SNO metabolism, we compared the available *S*-nitrosylomes obtained with several NO donors (Supplementary Table S2). As presented, we detected some overlap between the protein-SNO identified in this paper and those previously described, including stress- and redox-related proteins (HSP90-3; GPX, APX), members involved in signaling/regulating functions, or cytoskeleton members (ACT, TUB). We also spotted other candidates that have never been described in Arabidopsis as targets of protein-SNO. They include enzymes related to metabolism, such as galactokinase and redox-related proteins like nucleoredoxin 1 (NRX1), among others.

Finally, to test the results obtained by proteomics, we randomly selected 3 *S*-nitrosylated proteins identified in this study with commercially available antibodies (i.e. HSP90.2, tubulin, and actin). Figure 5D depicts how NO_2_-Ln is able to induce the *S*-nitrosylation of the 3 candidates. This validated the results obtained in this proteomic approach. More interestingly, we also observed that the 3 tested protein-SNOs exhibited a higher *S*-nitrosylation status in the *aer* mutant vs. Col-0 and differentially behaved in the *gsnor1* mutant background. These results confirm that NO_2_-Ln can modulate the *S*-nitrosylation pattern within the cell by differentially impacting protein-SNO.

## Discussion

Our results reveal that NO_2_-Ln modulates SNO content through the GSNOR1-SNO in Arabidopsis. NO_2_-FAs stem from the reaction of unsaturated fatty acids with NO and nitrite anion species (Schopfer et al. 2011; Koutoulogenis and Kokotos 2021). These molecules' bioactivity has been attributed largely to nitroalkene moiety. This makes it a strong Michael acceptor that can undergo nucleophilic attack from thiol groups of proteins (Geisler and Rudolph 2012). However, NO_2_-FAs' biological activity also relies on the capability to act as NO donors with similar capacity to GSNO (Mata-Pérez et al. 2016a). We recently observed that the NO released from NO_2_-Ln can interact with GSH to form GSNO. This result was confirmed in both in vitro and in vivo to evidence NO_2_-FA's role in GSNO metabolism in Arabidopsis (Mata-Pérez et al. 2020). NO enables downstream responses by PTMs, which mainly include *S*-nitrosylation or protein tyrosine nitration. Here, we analyzed the possibility of NO_2_-Ln to modulate these 2 NO-PTMs by NO release. Although no changes were detected in protein tyrosine nitration terms, interestingly, by several cellular and biochemical approaches, we found that NO_2_-Ln was able to prompt SNO accumulation in both cell cultures and seedlings of Arabidopsis plants. We used the *aer-*deficient mutant line to apply an in vivo approach because it accumulates higher NO_2_-Ln and GSNO levels (Mata-Pérez et al. 2020). Alkenal reductase (AER) emerged as a nitroalkene reductase found in Arabidopsis that performs similar functions to those attributed to prostaglandin reductase 1 (PGR1) in animals (Vitturi et al. 2013). Working with *aer* mutant-deficient line allowed us to simulate what we could similarly obtain by the application of exogenous NO_2_-Ln to our Arabidopsis model plant. Thus, we confirmed that endogenous NO_2_-Ln present in *aer* mutants is responsible for total SNO accumulation and, therefore, for protein-SNO. This allowed us to establish a direct link between NO_2_-FAs and the metabolism of SNOs.

Cellular GSNO levels are controlled by GSNOR1 as previously mentioned. Arabidopsis *GSNOR1* knockout mutants display increased protein-SNO levels due to the inability to remove GSNO. Thus, it is considered to be the enzyme that indirectly controls protein-SNO levels (Rustérucci et al. 2007; Lee et al. 2008; Kwon et al. 2012). Other enzymes are also emerging as denitrosylases in plant systems. One example is thioredoxin-*h*5 (TRX*h*5) oxidoreductase that directly targets a set of protein-SNO and plays a key role in plant immunity (Kneeshaw et al. 2014; Mata-Pérez and Spoel 2018). It has been recently shown that NO derived from nitrate uptake is able to suppress GSNOR1 activity by *S*-nitrosylation with the subsequent increase in SNO levels. This rise also leads to the suppression of the nitrate transporters and enzymes responsible for its reduction and therefore controlling total NO abundance in plants (Frungillo et al. 2014). Inasmuch as GSNOR1 indirectly controls SNO levels in plants together with NO_2_-Ln's capacity to modulate SNO content in Arabidopsis, in the *aer-*deficient mutants, we observed a significant decrease in the GSNOR1 transcript levels vs. the Col-0 plants. Although the precise mechanism has not yet been established, the electrophile adduction of HSP90 and HSP72 promotes dissociation from HSF1, which leads to binding and, therefore, to the activation of HSEs (Kansanen et al. 2009; Jacobs and Marnett 2010). Similarly, NO_2_-Ln might mediate conformational changes in TFs that affect their binding to GSNOR1 regulatory regions and therefore affect GSNOR1 expression.

A direct inhibitory effect of NO from nitrate reductase on GSNOR activity has been demonstrated (Frungillo et al. 2014). Our results showed that the GSNOR1 protein content in the *aer* mutant background slightly lowered concomitantly with a significant decrease in GSNOR1 enzymatic activity. As NO can affect GSNOR1 degradation, the constitutive expression of GSNOR1 under the CaMV35S promoter (*35S:Flag-GSNOR1* transgenic line) allowed us to study the pharmacological effect of NO_2_-Ln application on both total SNO content and GSNOR bioactivity without affecting protein stability. So we evidenced an increase in total SNO by NO_2_-Ln concomitantly with a decrease in the GSNOR function. This scenario suggests that the NO released from this NO_2_-FA impacts GSNOR bioactivity. Indeed, we noted a significant fall in recombinant GSNOR activity after NO_2_-Ln incubation, which was fully restored by cPTIO. All this reinforces the role of NO in the regulation of GSNOR bioactivity (Frungillo et al. 2014; Guerra et al. 2016; Tichá et al. 2017). We further confirmed that GSNOR is *S*-nitrosylated in vitro and in vivo by NO_2_-Ln. Indeed, the *S*-nitrosylation of GSNOR at Cys10 induces conformational changes that expose its autophagy-related 8 (ATG8)-interacting motif (AIM). So ATG8 is accessible by autophagy machinery and recruits GSNOR to degradation by selective autophagy, which is particularly relevant under hypoxic conditions (Zhan et al. 2018). In certain circumstances, our results highlight that NO_2_-Ln can release NO by targeting GSNOR for *S*-nitrosylation, which regulates its activity. This reinforces the idea that NO mediates feedback control over its own signaling pathway.

NO is a potent dormancy-releasing agent in many species, including Arabidopsis (Beligni and Lamattina 2000). NO_2_-FAs, such as NO_2_-Ln (Mata-Pérez et al. 2016c) and NO_2_-OA (Vollár et al. 2020), have been detected in early seed germination stages when NO burst takes place. Exogenous NO_2_-OA application to *Brassica napus* seeds induces the germination rate by a concomitant increase in the NO level, which suggests a role for NO_2_-FAs in this developmental stage (Vollár et al. 2020). We were able to accordingly obtain very similar results with NO_2_-Ln to endorse that the effect on seed germination is NO-mediated. These results were biologically proven in the *aer* mutants, where the germination rate was similar to that obtained with exogenous NO_2_-Ln. Several developmental functions of RNS result from interference with phytohormone signaling pathways, mostly by the *S*-nitrosylation of key intermediate signaling proteins. Regarding dormancy control, the ABA network governing seed dormancy involves the activity of bZIP TF ABI5. Crosstalk between NO and ABA has been demonstrated through ABI5 stability because NO assists the degradation of this TF by *S*-nitrosylation at Cys153 and by targeting it for degradation through the proteasome pathway (Albertos et al. 2015). We firstly observed that ABI5 levels significantly lowered in the *aer* mutant, which supports the data about the germination rates obtained in this mutant background. We further assessed ABI5 susceptibility to *S*-nitrosylation by NO_2_-Ln. As shown through a biotin switch, ABI5 is indeed susceptible to being *S*-nitrosylated by the NO released from this nitrated fatty acid. Seeds are enriched in unsaturated fatty acids that accumulate in endosperm, which consists of the main source of energy to break dormancy and to initiate seed germination. In fact, imbibition leads to the hydration of these lipid reservoirs concomitantly with NO burst (Beligni and Lamattina 2000; Bethke et al. 2004, 2006, 2007). In this context, we hypothesized that enzymes like phospholipases could mediate the release of NO_2_-FAs from cell membranes, where they could be esterified with complex lipids for downstream cell signaling actions (Freeman et al. 2008). NO_2_-Ln, which accumulates considerably in seeds (Mata-Pérez et al. 2016c), can release NO and modulate seed dormancy break in several ways. The NO from NO_2_-Ln can firstly contribute to the endogenous NO pool, which is necessary for seed dormancy break. Secondly, this NO can mediate the *S*-nitrosylation of specific targets that are relevant in the control of this physiological process, i.e. the master regulator of growth repression ABI5. NO_2_-Ln-mediated *S*-nitrosylation can affect proteasome-dependent ABI5 degradation, which leads to germination onset. Thirdly, we cannot rule out the *S*-nitrosylation of GSNOR in the early plant development stages that might control the NO pool and induce protein-SNO and may be needed for germination to take place.

We were interested in identifying the proteins that are modified through *S*-nitrosylation by NO_2_-Ln. Despite the increasing number of protein-SNO identified and characterized in many eukaryotes, most of these approaches have been performed by using NO donor GSNO. We thought it would be meaningful to identify protein-SNO modulated by NO_2_-Ln. Indeed, the proteomic analyses resulted in the identification of members related to the response to stimuli, signaling/regulating, redox-related, protein folding/degradation, metabolic, and other proteins. By making a comparison to other *S*-nitrosylomes (Lindermayr et al. 2005; Kato et al. 2012; Niu et al. 2019), we detected some overlap with our proteomics findings. For instance, of the *S*-nitrosylated proteins herein identified, the enzymes related to GSH metabolism (GSH *S*-transferase or peroxidase), HSP 90-3, peroxiredoxins, many cytoskeleton proteins (tubulin, actin, annexin), and many other metabolic enzymes (fructose bisphosphate aldolase, triosephosphate isomerase, GAPDH, enolase, among others) have already been identified to undergo *S*-nitrosylation in *A. thaliana*, *Solanum tuberosum*, or *Cucumis sativus*. Nevertheless, interesting candidates were spotted that involve the malic enzyme, galactokinase 1, nucleoredoxin 1, or thioredoxin *h*3 and *h*5 proteins, among many others. These results highlight that NO_2_-Ln also prompts a specific manner to modulate protein *S*-nitrosylation.

Transcriptomic analyses on the role of NO_2_-FA have shown the involvement of the heat shock response (HSR) through the induction of HSP expression (Kansanen et al. 2009; Mata-Pérez et al. 2016c). Previous findings have demonstrated that NO_2_-FAs can mediate an electrophilic modification of their targets (i.e. nitroalkylation of members of the HSR) by altering the capacity to bind to promoter regions of heat shock binding elements (HSEs) (Jacobs and Marnett 2010). From the protein-SNOs mediated by NO_2_-Ln, we found several HSP family members. This is not the first time that the *S*-nitrosylation of members of this family has been evidenced because HSC70-1 can be endogenously modified at Cys319 in Arabidopsis cultures (Fares et al. 2011), and GSNO prompts the *S*-nitrosylation of HSP90-3 (Lindermayr et al. 2005). Interestingly, we found the *S*-nitrosylation of different ubiquitin-related proteins, which suggests that NO_2_-Ln-mediated S-nitrosylation might be involved in regulating the ubiquitination process. Indeed the interplay between *S*-nitrosylation and ubiquitination has been demonstrated in several phytohormone signaling pathways, including the control of seed germination by ABA (Albertos et al. 2015) or immune responses through the SA signaling pathway (Tada et al. 2008). *S*-Nitrosylation has also been reported to affect a variety of PTMs, including ubiquitination, phosphorylation, or SUMOylation (Hess and Stamler 2012).

Interestingly, we did not detect GSNOR1 among the proteins identified in our proteomic study. Indeed, GSNOR1 has not been previously detected in other protein-SNO studies (Lindermayr et al. 2005; Romero-Puertas et al. 2008; Fares et al. 2011; Kato et al. 2012) despite it being described as a target of *S*-nitrosylation (Frungillo et al. 2014). Additionally, GSNOR1-SNO has been described to possibly lead to its degradation by selective autophagy (Zhan et al. 2018). In our experimental design, we cannot determine to what extent NO_2_-Ln might interfere with GSNOR1 stability but the treatment of ACSC with NO_2_-Ln increased S-nitrosylation of GSNOR, and could be the reason why we were unable to find GSNOR1-SNO among the identified SNO-proteins in the proteomic study. We believe that ABI5, a crucial TF for seed break dormancy with a marked expression in early plant development stages, is not present among the *S*-nitrosylated proteins detected by our proteomics study because we employed cell suspension cultures where ABI5 is essentially not needed. As *S*-nitrosylation of ABI5 leads to its degradation through the proteasome pathway (Albertos et al. 2015), we cannot rule out that a combination of both factors could be responsible for not detecting ABI5-SNO in this study.

**Figure 6.**
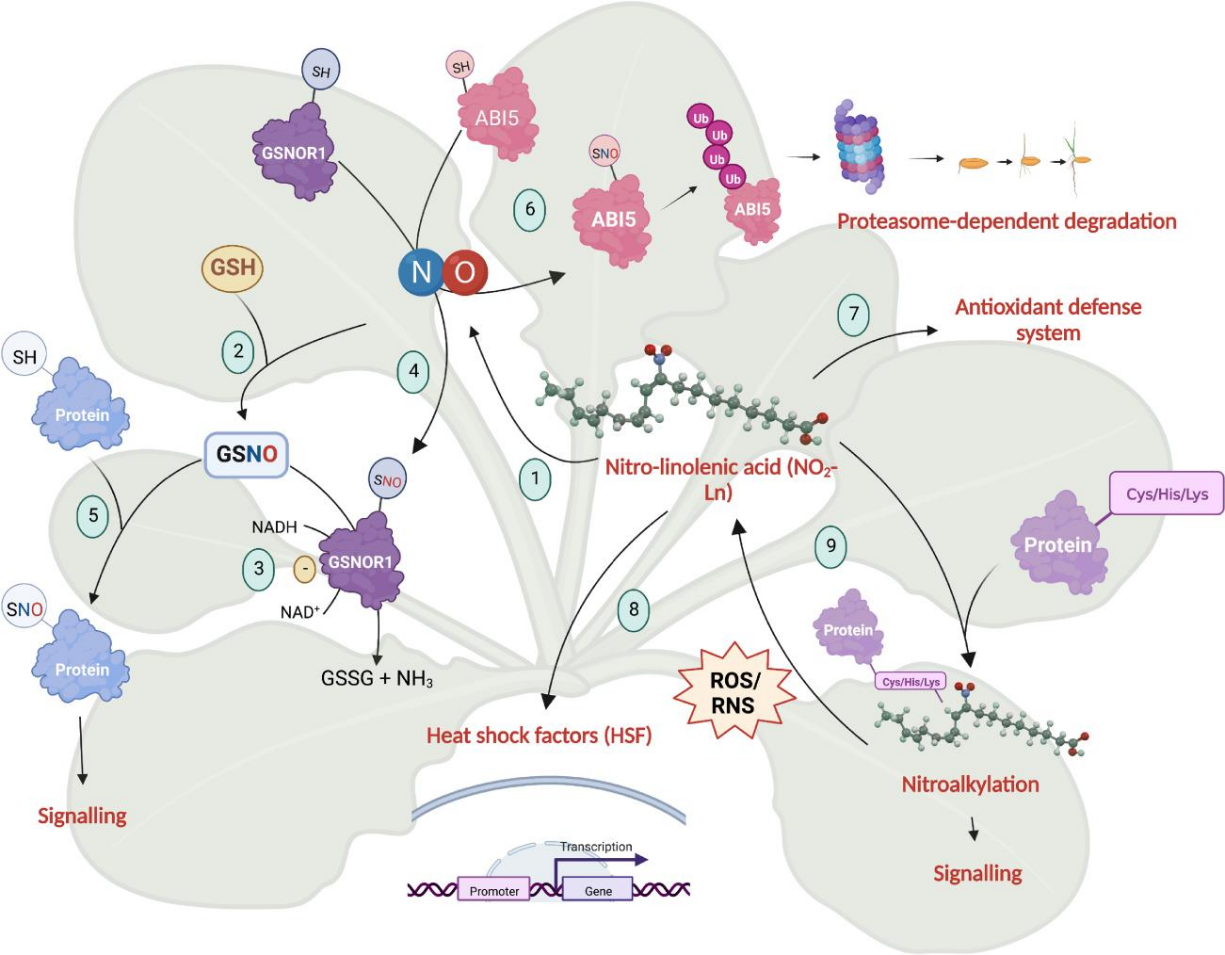
Proposed model. NO_2_-Ln acts as a physiological NO donor (1) that binds to intracellular GSH to form GSNO in vivo, the main reservoir of NO in cells (2) (Mata-Pérez et al. 2016a, 2016b, 2020). GSNOR1 catalyzes the GSNO breakdown (3) and it can also be *S*-nitrosylated by the NO released from NO_2_-Ln to consequently reduce its enzymatic activity (4). This inhibition leads to an increase in available GSNO and indirectly to a rise in intracellular protein-SNO content (5). NO_2_-Ln's ability to release NO can target ABI5 for *S*-nitrosylation and subsequent proteasome-dependent degradation (6) to lead to seed germination. This NO_2_-FA is able to drive the expression of the HSR and antioxidant defense systems, which may mediate abiotic stress responses in plants (7, 8) (Mata-Pérez et al. 2016c). Finally, NO_2_-Ln can act on nucleophilic protein residues via a reversible PTM called nitroalkylation, which can modulate protein function (9) (Padilla et al. 2017; Aranda-Caño et al. 2019). Created with BioRender.com.

Finally, as a summary about the functional implications of NO_2_-Ln in plant physiology, we propose a model that includes what we know so far about NO_2_-Ln signaling in Arabidopsis (Fig. 6). NO_2_-Ln acts as a physiological NO donor (1) that binds to intracellular GSH to form GSNO in vivo, the main reservoir of NO in cells (2) (Mata-Pérez et al. 2016a, 2016b, 2020). GSNOR1 catalyzes the breakdown of GSNO (3) and it can also be *S*-nitrosylated by the NO released from NO_2_-Ln to consequently reduce its enzymatic activity (4). This inhibition leads to an increase in available GSNO and indirectly to a rise in intracellular protein-SNO content (5). Furthermore, NO_2_-Ln's ability to release NO can target ABI5 for *S*-nitrosylation and subsequent proteasome-dependent degradation (6) to lead to seed germination. This NO_2_-FA is able to drive the expression of the HSR and antioxidant defense systems, which may mediate abiotic stress responses in plants (7, 8) (Mata-Pérez et al. 2016c). Finally, NO_2_-Ln can act on nucleophilic protein residues through a reversible PTM called nitroalkylation, which can modulate the protein function (9) (Padilla et al. 2017; Aranda-Caño et al. 2019).

## Materials and methods

### Plant material

For different purposes, the Arabidopsis (*A. thaliana*) accession Col-0 was used as WT. Mutant *gsnor1-3/hot5-2*, (*GSNOR1*, AT5G43940) and the *35S:FLAG-GSNOR1* (Col-0) construct (reported by Frungillo et al. 2014) were kindly provided by Steven Spoel, PhD (University of Edinburgh, Scotland, United Kingdom). The Alkenal Reductase (*AER*, ATG16970)-deficient line with a T-DNA insertion in the promoter region was obtained from the Arabidopsis Biological Resource Center (ABRC, line SALK 005324C). The homozygous line was characterized as previously described (Mata-Pérez et al. 2020).

### Growth conditions

For the in vitro culture, *A. thaliana* seeds were surface-sterilized as described elsewhere (Mata-Pérez et al. 2016c). Seeds were vernalized for 48 h at 4 °C in the dark and then sown on MS solid medium for 14 d. Seedlings were incubated with 100 *µ*m linolenic acid (Ln) or 100 *µ*m NO_2_-Ln, or were not treated, for 30 min under the same growing conditions.

The 9-d-old ACSCs were grown as previously described (Mata-Pérez et al. 2016c). Treatments on ACSCs were performed by adding 100 *µ*m linolenic acid (Ln) or 100 *µ*m NO_2_-Ln, or were not treated, for 1 h and left under the same growing conditions. The cell pellet was later obtained as described in Mata-Pérez et al. (2016a, 2016c).

### NO_2_-Ln synthesis

As NO_2_-Ln is not commercially available, we carried out NO_2_-Ln synthesis by a nitroselenation–oxidation–hydroselenoxide elimination sequence as described elsewhere (Mata-Pérez et al. 2016c, 2020; Aranda-Caño et al. 2022a). Briefly, linolenic acid was incubated with solid mercury chloride, phenylselenyl bromide, and sodium nitrite in a mixture of tetrahydrofuran-acetonitrile (1:1, v/v). After filtration, the residue was left to cool down to 0 °C, incubated with 30% H_2_O_2_ solution and mixed for 20 min. Next, the reaction was extracted with hexane and washed with brine, dried, filtered, and evaporated to dryness. The residue was then purified by flash column chromatography with a mixture of hexane–ether–acetic acid (80:20:1, v/v/v) by ensuring the purification of mono-nitrated NO_2_-Ln. Fractions were analyzed by TLC, and the samples containing NO_2_-Ln were then pooled and dried by vacuum. Spectra were analysed by NMR and LC-MS/MS.

### NO_2_-Ln treatment of *A. thaliana* seeds and germination assays

The seed lots to be compared were harvested on the same day from the individual plants grown under identical environmental conditions. To break seed dormancy and obtain after-ripened seeds, they were stored for 10 d at room temperature. The Col-0 or *aer* after-ripened seeds were incubated in dH_2_O containing methanol, Ln, NO_2_-Ln, a combination of NO_2_-Ln with cPTIO (2-4-carboxyphenyl-4,4,5,5-tetramethylimidazoline-1-oxyl-3-oxide), cPTIO alone, GSNO, or untreated. All the treatments were maintained in an orbital shaker for 24 h in the dark. Next, seeds were washed 3 times to remove any leftover and were sown on 5 mm MES with 0.8% agar at pH 5.8.

### Immunoblotting

The total protein from ACSCs and the 14-d-old seedlings was extracted as described elsewhere (Mata-Pérez et al. 2016c). Tissue was powdered using a mortar and pestle and was incubated for 5 min with extraction buffer (50 mm Tris-HCl, pH 7.5, 150 mm NaCl, 0.25% NP-40, 1 mm PMSF, and 1X protease inhibitor cocktail, Sigma) followed by centrifugation for 15 min at 14,000 × *g* at 4 °C. The protein concentration was determined in all cases by the Bio-Rad Protein Assay (Bio-Rad) and subjected to immunoblotting. For immunodetection, the anti-3-nitrotyrosine (NO_2_-Tyr, 1:8,000) (Chaki et al. 2009b), anti-GSNOR1 (Agrisera, 1:1,000), anti-ABI5 (Biomedal, 1:10,000), anti-Actin clone 10-B3 (Sigma, 1:10,000), anti-HSP90.2 (Agrisera, 1:3,000), anti-tubulin alpha chain (Agrisera, 1:1,000), ECL-Peroxidase-labelled anti-rabbit (Amersham; 1:10,000), and anti-mouse (Amersham; 1:10,000) antibodies were used for several immunoblot analyses. Detection was carried out using ECL (ECL-PLUS; Amersham), and chemiluminescence was detected with either photographic film (Hyperfilm; Amersham) or the ChemiDoc MP Imaging System (Bio-Rad).

### Expression and purification of *A. thaliana* GSNOR1

cDNA was obtained from total Arabidopsis leaf RNA by using the first-strand cDNA synthesis kit (Roche, Basel, Switzerland). Then, GSNOR1 amplification (AY087250.1) was performed by PCR using FastStart High-Fidelity polymerase (Roche, Basel, Switzerland) and specific direct and reverse primer sets: 5′-GGATCCAATGGCGACTCAAGGTCAGGTTATC-3′ and 5′-GAAAGCTCGAGTGTCATTTGCTGGTATCGAG-3′, respectively. The PCR product (1,158 bp) was cloned into the pALEXb vector (Biomedal SL, Seville, Spain), and the expression of the recombinant protein carrying an N-terminal choline-binding domain (hereinafter referred to as Lytag) was performed according to Begara-Morales et al. (2014, 2019). The recombinant protein was induced by adding 1 mm salicylate and 10 mm 3-methyl benzoate. Protein purification was then carried out using a 1-mL LYTRAP column (Biomedal SL, Seville, Spain), in which protein was eluted in 1 mL fractions employing a discontinuous gradient of choline. Lytag (21.28 kDa) addition to the GSNOR1 Arabidopsis protein (40.7 kDa) resulted in a molecular weight of 62 kDa. Finally, the purity grade of the recombinant protein expression was analysed by 10% SDS-PAGE.

### GSNOR1 enzymatic assay

GSNOR1 activity was measured spectrophotometrically (Barroso et al. 2006; Chaki et al. 2011b) by determining NADH oxidation at 340 nm over time.

With the Lytag recombinant GSNOR1 protein, 100 *µ*m Ln, NO_2_-Ln, GSNO, or SNAP (*S*-nitroso-*N*-acetylpenicillamine) were incubated for 30 min at 25 °C. Then, enzymatic activity was determined. For the cPTIO and DTT treatments, the GSNOR1 recombinant protein was concomitantly incubated with 100 *µ*m NO_2_-Ln and 500 *µ*m cPTIO or 5 mm DTT for 30 min at 25 °C. This was followed by NADH oxidation at 340 nm for 10 min.

### SNO determination by ozone chemiluminescence

Total SNO content was determined by the method described in (Chaki et al. 2009a, 2011b). All the operations were performed at 0 to 4 °C under a red safety light to protect SNO from light-dependent decomposition. Two aliquots were prepared per sample: (i) treated with 10 mm sulphanylamide for 15 min to remove nitrite and (ii) treated with 10 mm sulphanylamide for 15 min and 7.3 mm HgCl_2_ for 15 min to remove nitrite and SNO, respectively. Samples were analysed in a NO analyzer (NOA 280i Sievers). The data from (i) and (ii) represented the total SNO concentration.

### SNO detection by confocal laser scanning microscopy

For the confocal microscopy analysis, SNOs were detected using the Alexa Fluor 488 Hg-link (Thermo Fisher) phenylmercury fluorescent reagent prepared in PBS as described elsewhere (Chaki et al. 2011a). Cell cultures were incubated with methanol (vehicle), 100 *µ*m Ln, or 100 *µ*m NO_2_-Ln for 1 h. Next, samples were incubated with 100 *µ*m diethylenetriaminepentaacetic acid (DTPA) and 10 mm*N*-ethylmaleimide (NEM) for 2 h at 25 °C to block free sulfhydryl groups (-SH). Following DTPA and NEM incubation, samples were washed 3 times with 10 mm PBS for 15 min each. Then, these samples were incubated with 10 *µ*m Alexa Fluor 488 Hg-link phenylmercury (Molecular Probes, Eugene, Oregon, United States, cat. No. H30462) for 1 h at 25 °C in the dark and washed 3 times in PBS for 15 min each.

Regarding negative controls and after incubating cell cultures with 100 *µ*m NO_2_-Ln and washing 3 times with 10 mm PBS, 1 sample was incubated with DTPA and NEM as stated above, followed by 3 washes with 10 mm PBS. Alexa Fluor 488 Hg-link phenylmercury was not added to this sample. Likewise, samples were not previously incubated with DTPA and NEM. Then, after the following washes with PBS, the fluorophore was added to the sample and used as a negative control for the signal given by Alexa Fluor 488 Hg-link phenylmercury fluorophore.

### RT-qPCR

Total RNA was extracted as described elsewhere (Mata-Pérez et al. 2016c), and RT-qPCR was performed in a CFX96 real-time PCR Detection System (Bio-Rad). *GSNOR1* amplification was carried out with specific primers (Supplementary Table S1), and results were normalized using *18S rRNA* (AT2G01010) and *ribosomal protein L2 family* (AT2G44065) as internal controls.

### In vitro and in vivo *S*-nitrosylation assays

For *S*-nitrosylation detection purposes, we followed the BST (Jaffrey and Snyder 2001). This PTM was analyzed in vitro for the recombinant GSNOR1 and ABI5 proteins and in vivo for the ACSC and 14-d-old Col-0 and *aer* seedlings incubated with NO_2_-Ln as previously described.

For in vitro *S*-nitrosylation, the purified GSNOR1 and ABI5 recombinant proteins were incubated with either 1 mm GSNO or 100 *µ*m NO_2_-Ln as NO donors in the dark at room temperature for 1 h with gentle shaking. Reagents were removed by precipitation with 2 volumes of −20 °C acetone, and proteins were assayed by the BST. To ensure specificity, sodium ascorbate (SNO-specific) removal and the 20 mm DTT application (SNO break) served as negative controls.

In vivo *S*-nitrosylation was carried out in ACSCs and the 14-d-old Col-0 and *aer* seedlings protein extracts incubated with 100 *µ*m NO_2_-Ln. The different experimental conditions were subjected to the BST as previously described (Begara-Morales et al. 2014). All the *S*-nitrosylation assays were performed independently 3 times. The figures show the most representative result.

To demonstrate that sodium ascorbate removal and DTT application have a similar effect on the S-nitrosylation pattern mediated by NO_2_-Ln, we used protein extracts from 14 d Arabidopsis seedlings. Then, the extracts were treated as mentioned above and subjected to the BST using both negative controls. Supplementary Fig. S4 shows NO_2_-Ln's *S*-nitrosylating capacity, and the addition of DTT reduces the formation of SNOs after NO_2_-Ln treatment, consistent with the presence of a reversible thiol modification. The biotin switch was also assayed without ascorbate to assure that the immune reactivity of the protein was ascorbate dependent and to prevent switching SNO for biotin.

The protein-SNO of the Arabidopsis cell cultures treated with methanol, 100 *µ*m Ln, or 100 *µ*m NO_2_-Ln were purified on a streptavidin resin (Sigma) and used for identification by MS approaches (LC-MS/MS).

### Protein-SNO detection and identification by MS analysis

In an attempt to gain an overview of the proteins potentially *S*-nitrosylated by NO_2_-Ln, the protein-SNO from the ACSCs treated with methanol, 100 *µ*m Ln, or 100 *µ*m NO_2_-Ln (as shown above in Materials and methods) were subjected to a MS analysis. Three biological replicates from each condition were subjected to the BST and pooled after purification to obtain the protein-SNO of the 3 experimental situations for the proteomics analysis.

Before digestion, 50 *µ*g of protein-SNO from each condition was reduced by adding 50 mm DTT at 95 °C for 5 min. Then, samples were centrifuged at 1,600 × *g* and 13 °C for 5 min to rule out any traces of suspended material before filter-aided sample preparation digestion.

For protein digestion, samples were filtered and centrifuged for 14 min at 14,000 × *g*. Then, urea buffer was passed through the filter to discard flow through. The proteins retained in the membrane were alkylated with 0.05 m iodoacetic acid at 650 × *g* for 1 min, followed by another 20-min period without shaking in the dark. Filters were centrifuged at 14,000 × *g* for 10 min and washed with urea buffer, followed by 3 washes with 200 mm triethylammonium bicarbonate (TEAB) buffer. For protein digestion, 20 ng/*µ*L trypsin was dissolved in 200 mm TEAB buffer and digested overnight at 37 °C. The digested peptides were recovered by centrifuging for 15 min at 14,000 × *g*. Three washes with TEAB buffer were performed and followed by sample acidification with 1% trifluoroacetic acid.

Next, samples were dried and dissolved in 200 *µ*L of 30% acetonitrile containing 0.1% formic acid for subsequent separation by ion exchange chromatography to reduce sample complexity. Chromatography was performed as follows: phase A: 30% acetonitrile, 0.1% formic acid; phase B: 30% acetonitrile, 0.1% formic acid, 500 mm ammonium chloride. Peptides were gradually eluted by using higher ammonium chloride concentrations, and 6 fractions were collected. Fractioning was monitored by an UV detector at 214 nm.

For peptide sequencing, the different samples were injected into a chromatographic system equipped with a C18 preconcentration column (300 *µ*m id × 0.5 cm) and an analytical column (100 *µ*m id × 15 cm). Peptides were introduced into the preconcentration column using 1% formic acid as a solvent and eluted to the analytical column at a flow rate of 300 nL/min using a gradient of 3% to 35% acetonitrile/0.1% formic acid for 120 min. The chromatographic system was online connected to a high-resolution mass spectrometer LTQ-Orbitrap XL (Thermo Fisher) to run the analysis in the dependent scan mode: a full scan using Orbitrap at the 6,000 resolution and 8 parallel MS/MS scans (4 in the CID mode and 4 in the HCD mode) of the most abundant precursors.

Peptide identification was performed by the Protein Discovery 2.5 software of Thermo Instruments using a 1% false discovery rate. The peptides identified as unique (peptide sequences that can belong only to 1 protein where at least 2 peptides were detected) were considered for the mass spectrometric analysis. Then 1,520 unique peptide spectra defining 593 proteins were detected.

Specifically, a first comparison between the protein-SNO detected in methanol and the Ln-treated cell suspension cultures was made to rule out the proteins that could be *S*-nitrosylated by fatty acid alone (Ln). This protein-SNO set was then compared to the NO_2_-Ln-treated cell cultures to know the identity of protein-SNO as shown in Supplementary Table S1.

### Bioinformatics

A gene set enrichment analysis was carried out using PANTHER, version 16 (Mi et al. 2021). The enrichment of the GO terms from the proteomic analysis was determined in relation to the GO term frequency in the Arabidopsis genome (TAIR10 2022 assembly). Statistical significance was calculated by Fisher's exact test and the Bonferroni correction for multiple testing to select the GO terms with adjusted *P* < 0.05.

### Statistical analysis

For the germination data, an ANOVA and/or its nonparametric version (Kruskal–Wallis test) was performed to investigate whether there were significant differences among samples (genotypes, treatments, time points). Bonferroni correction (or Dunnett test) was performed if a significant difference was found to determine which samples were responsible for these significant differences.

### Accession numbers

Sequence data from this article can be found in the GenBank/EMBL data libraries under accession numbers AT5G43940 (GSNOR1) and ATG16970 (AER).

## Supplementary Material

kiaf038_Supplementary_Data

## Data Availability

There are no data available other than those published in the manuscript and in the supplementary material. Furthermore, these data are openly available to the scientific community.

## Supplementary data

The following materials are available in the online version of this article.

**Supplementary Figure S1.** NO_2_-Ln does not lead changes in protein tyrosine nitration levels.

**Supplementary Figure S2.** NO_2_-FAs play a role in seed dormancy breakage.

**Supplementary Figure S3.** GO terms.

**Supplementary Figure S4.** Ascorbate and DTT specificity in the *S*-nitrosylation mediated by NO_2_-Ln.

**Supplementary Table S1.** List of the protein-SNOs mediated by NO_2_-Ln.

**Supplementary Table S2.** Selected candidates of the identified *S*-nitrosylated proteins in the plant extracts treated with several NO donors.

**Supplementary Table S3.** List of primers for RT-qPCR.

## Dive Curated Terms

The following phenotypic, genotypic, and functional terms are of significance to the work described in this paper:

HSF1 Gramene: AT4G17750
HSF1 Araport: AT4G17750

PRXIIF Gramene: AT3G06050
PRXIIF Araport: AT3G06050

PTM AmiGo: PO:0005039

APX1 Gramene: AT1G07890
APX1 Araport: AT1G07890

GSNOR Gramene: AT5G43940
GSNOR Araport: AT5G43940
