# Supplementary material for: Nitro-fatty acids modulate germination onset through S-nitrosothiol metabolism
Source: Plant Physiol. 2025 Jan 25;197(2):kiaf038. doi: 10.1093/plphys/kiaf038 (PMC11831805; doi:10.1093/plphys/kiaf038)
Supplement: kiaf038_Supplementary_Data [file kiaf038_supplementary_data.zip › kiaf038_Supplementary_Data.pdf]

(a)

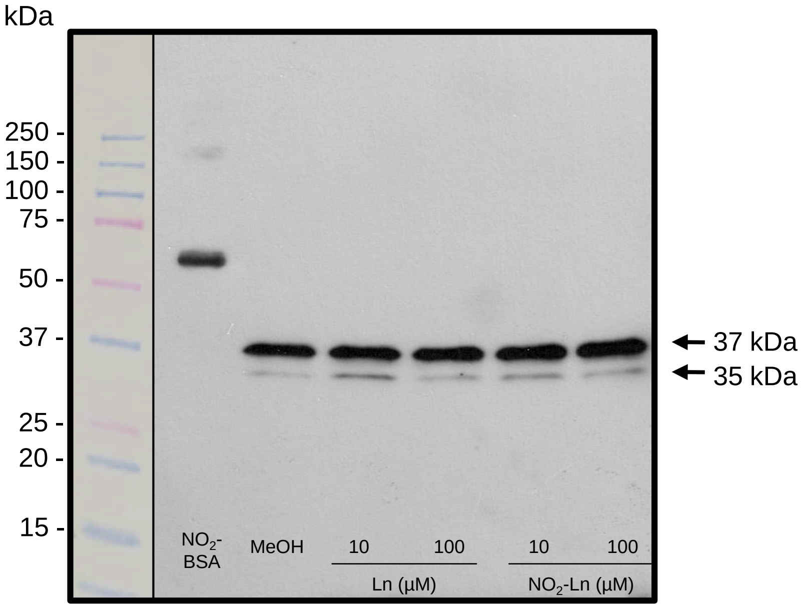

kDa
250 -
150 -
100 -
75 -
50 -
37 -
25 -
20 -
15 -
NO2-BSA
MeOH
10
100
10
100
Ln (µM)
NO2-Ln (µM)
37 kDa
35 kDa

(b)

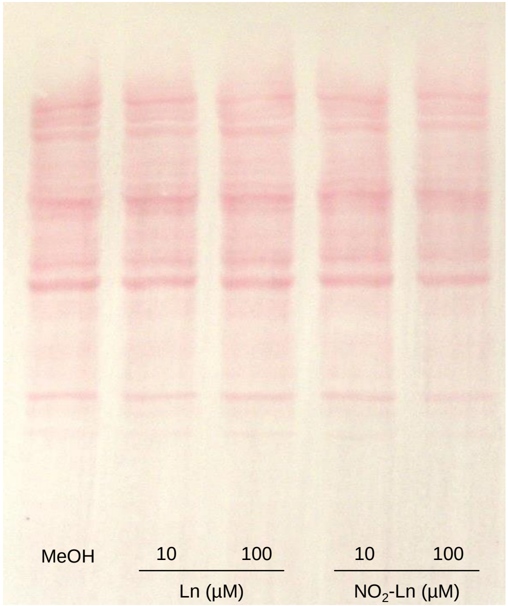

MeOH
10
100
10
100
Ln (µM)
NO2-Ln (µM)

(c)

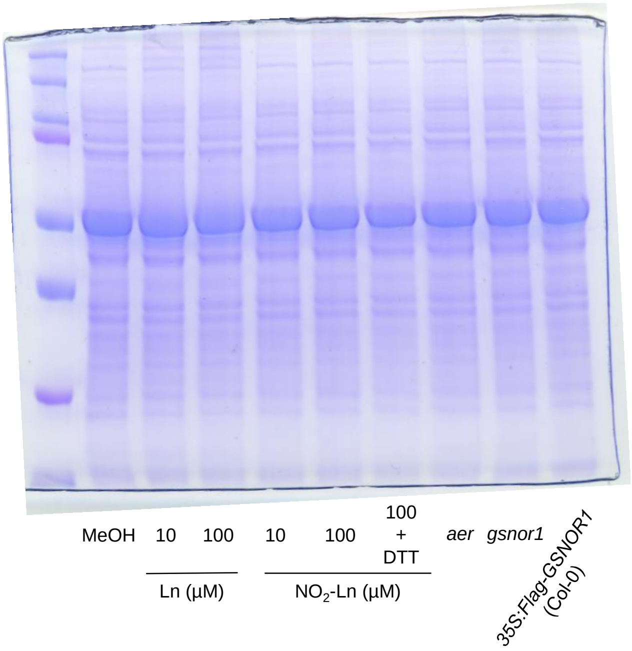

MeOH
10
100
10
100
100 + DTT
aer
gsnor1
35S:Flag-GSNOR1 (Col-0)
Ln (µM)
NO2-Ln (µM)

Supplementary Figure S1. NO_2-Ln does not lead changes in protein tyrosine nitration levels. A) Protein tyrosine nitration modulation by nitro-linolenic acid (NO_2-Ln). A representative immunoblot showing protein tyrosine nitration in the crude extracts of the Arabidopsis cell-suspension cultures (ACSCs) incubated with 10 and 100 µM NO_2-Ln or Ln. Samples (10 µg of protein per lane) were subjected to sodium dodecyl sulphate-polyacrylamide gel electrophoresis (SDS–PAGE) and a Western blotting analysis using an antibody against 3-nitrotyrosine (NO_2-Tyr) (dilution 1: 1500). From left to right: commercial nitrated Bovine Serum Albumin (NO_2-BSA) (5 µg of protein) was used as the positive control; ACSCs pre-incubated with methanol, 10 and 100 µM Ln, and with 10 and 100 µM NO_2-Ln. The numbers on the left side of the immunoblot indicate the relative molecular masses of protein markers. **B)** Ponceau staining of the ACSCs treated with NO_2-Ln. ACSCs pre-incubated with methanol, 10 and 100 µM Ln, and with 10 and 100 µM NO_2-Ln. A 10-µg aliquot of protein from each situation was used per lane. Then proteins were separated under non-reducing (NR) conditions by 10% SDS-PAGE and blotted onto a Polyvinylidene Fluoride (PVDF) membrane as described in Methods. **C)** Coomassie staining for the total protein loading from the 9-day-old seedlings (Figure S1c) used in Figure 1d. From left to right: methanol; 10 and 100 µM Ln; 10 and 100 µM NO_2-Ln; DTT-induced SNO break (served as a control); *aer*, *gsnor1* and *35S:Flag-GSNOR1* construction in Col-0.

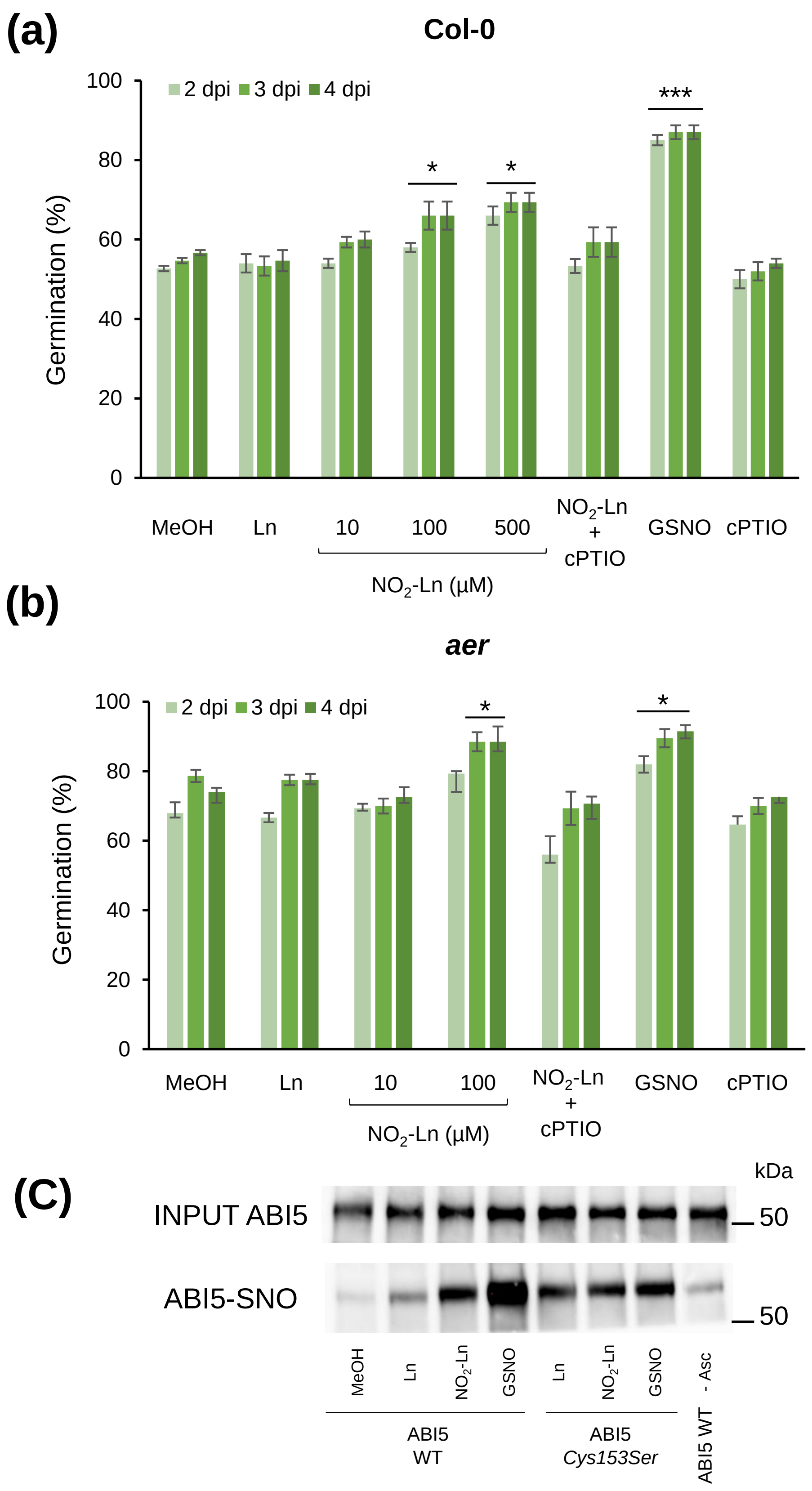

(a)
Col-0
2 dpi 3 dpi 4 dpi
Germination (%)
100
80
60
40
20
0
*
*
***
MeOH
Ln
10
100
500
NO2-Ln + cPTIO
GSNO
cPTIO
NO2-Ln (µM)
(b)
aer
2 dpi 3 dpi 4 dpi
Germination (%)
100
80
60
40
20
0
*
*
MeOH
Ln
10
100
NO2-Ln + cPTIO
GSNO
cPTIO
NO2-Ln (µM)
(C)
kDa
INPUT ABI5
50
ABI5-SNO
50
MeOH
Ln
NO2-Ln
GSNO
Ln
NO2-Ln
GSNO
- Asc
ABI5 WT
ABI5 Cys153Ser
ABI5 WT

Supplementary Figure S2. Nitro-fatty acids play a role in seed dormancy breakage. **A)** and **B)** Germination rate of the Col-0 and *aer* seeds after different treatments. **A)** Germination of the wild-type (Col-0) seeds in media containing methanol (MeOH), 500 µM Ln, 10, 100 and 500 µM NO_2-Ln, a combination of 500 µM NO_2-Ln with 1 mM 2-(4-Carboxyphenyl)-4,4,5,5-tetramethylimidazoline-1-oxyl-3-oxide potassium salt (cPTIO), 1 mM S-Nitrosoglutathione (GSNO), or 1 mM cPTIO. **B)** Germination of the *aer* seeds in media containing methanol (control), 100 µM Ln, 10 and 100 µM NO_2-Ln, a combination of 100 µM NO_2-Ln with 300 µM cPTIO, 1 mM GSNO or 300 µM cPTIO. Percentage of seeds with an emerged radicle (germination) was determined 2, 3 and 4 days after sowing. Each value represents the average germination percentage of 60 seeds with the s.e. of three replicates. Experiments were done at least 3 times. Bonferroni test was performed to determine any statistical differences between treatments ((*$p < 0.05$,***$p < 0.001$. **C)** Biotin switch of the wild type (WT) and Cys153Ser versions of the recombinant ABI5 in the presence of NO_2-Ln. The ABI5 WT was incubated with methanol, 100 µM Ln, NO_2-Ln or GSNO. ABI5 *Cys153Ser* incubated with 100 µM Ln, NO_2-Ln or GSNO. The ABI5 WT was incubated with 100 µM NO_2-Ln in the absence of ascorbate and used as the negative control. Total ABI5 protein ensures equal protein loading. The position of a 50 kDa marker is indicated.

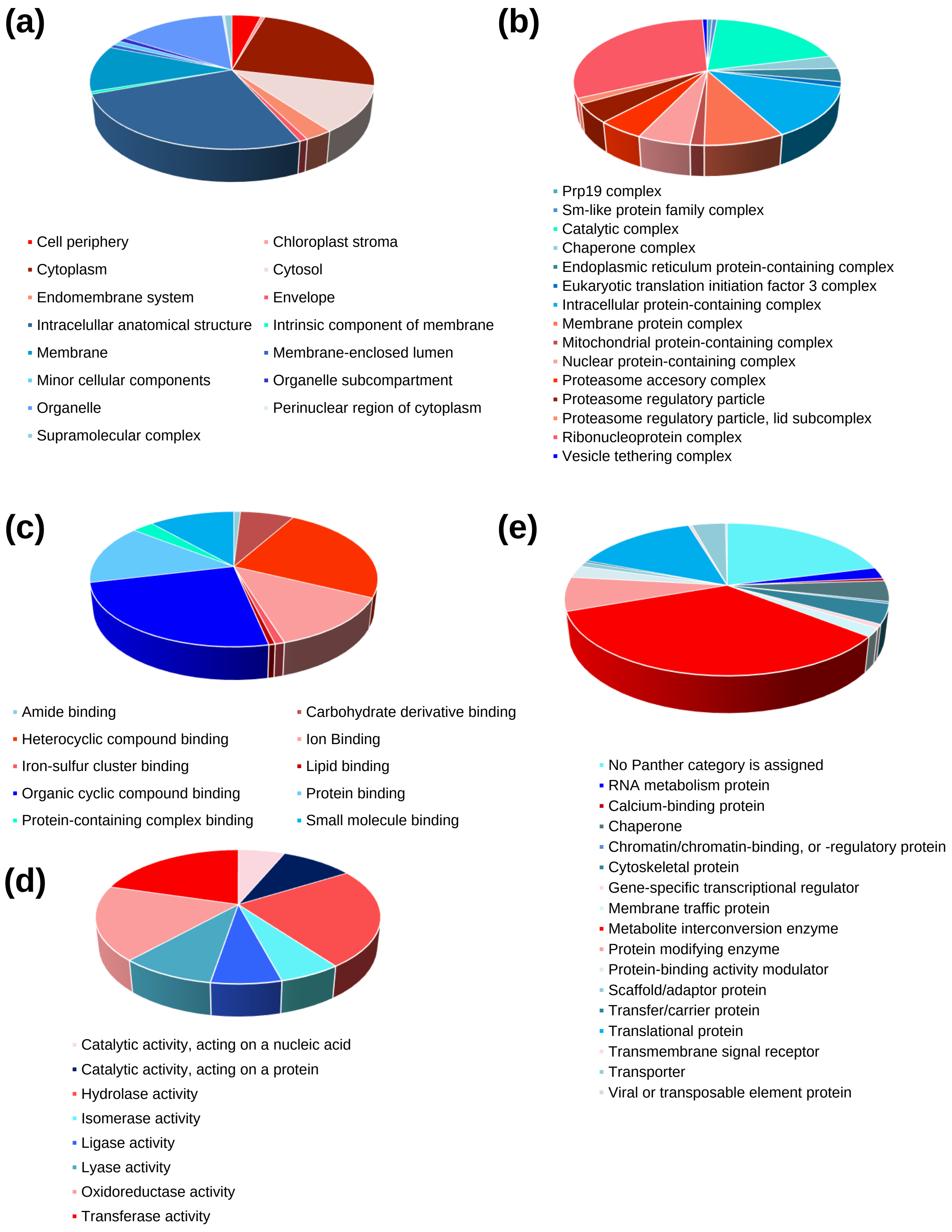

(a)
Cell periphery
Chloroplast stroma
Cytoplasm
Cytosol
Endomembrane system
Envelope
Intracelullar anatomical structure
Intrinsic component of membrane
Membrane
Membrane-enclosed lumen
Minor cellular components
Organelle subcompartment
Organelle
Perinuclear region of cytoplasm
Supramolecular complex
(b)
Prp19 complex
Sm-like protein family complex
Catalytic complex
Chaperone complex
Endoplasmic reticulum protein-containing complex
Eukaryotic translation initiation factor 3 complex
Intracellular protein-containing complex
Membrane protein complex
Mitochondrial protein-containing complex
Nuclear protein-containing complex
Proteasome accesory complex
Proteasome regulatory particle
Proteasome regulatory particle, lid subcomplex
Ribonucleoprotein complex
Vesicle tethering complex
(c)
Amide binding
Carbohydrate derivative binding
Heterocyclic compound binding
Ion Binding
Iron-sulfur cluster binding
Lipid binding
Organic cyclic compound binding
Protein binding
Protein-containing complex binding
Small molecule binding
(d)
Catalytic activity, acting on a nucleic acid
Catalytic activity, acting on a protein
Hydrolase activity
Isomerase activity
Ligase activity
Lyase activity
Oxidoreductase activity
Transferase activity
(e)
No Panther category is assigned
RNA metabolism protein
Calcium-binding protein
Chaperone
Chromatin/chromatin-binding, or -regulatory protein
Cytoskeletal protein
Gene-specific transcriptional regulator
Membrane traffic protein
Metabolite interconversion enzyme
Protein modifying enzyme
Protein-binding activity modulator
Scaffold/adaptor protein
Transfer/carrier protein
Translational protein
Transmembrane signal receptor
Transporter
Viral or transposable element protein

Supplementary Figure S3. Gene ontology (GO) terms. A) Cellular anatomical entity and **B)** protein-containing complex categories in the GO term of cellular components. **C)** binding and the **D)** catalytic activity categories in the GO term of molecular functions. **E)** Protein class classification. All the analyses were carried out with the Panther software.

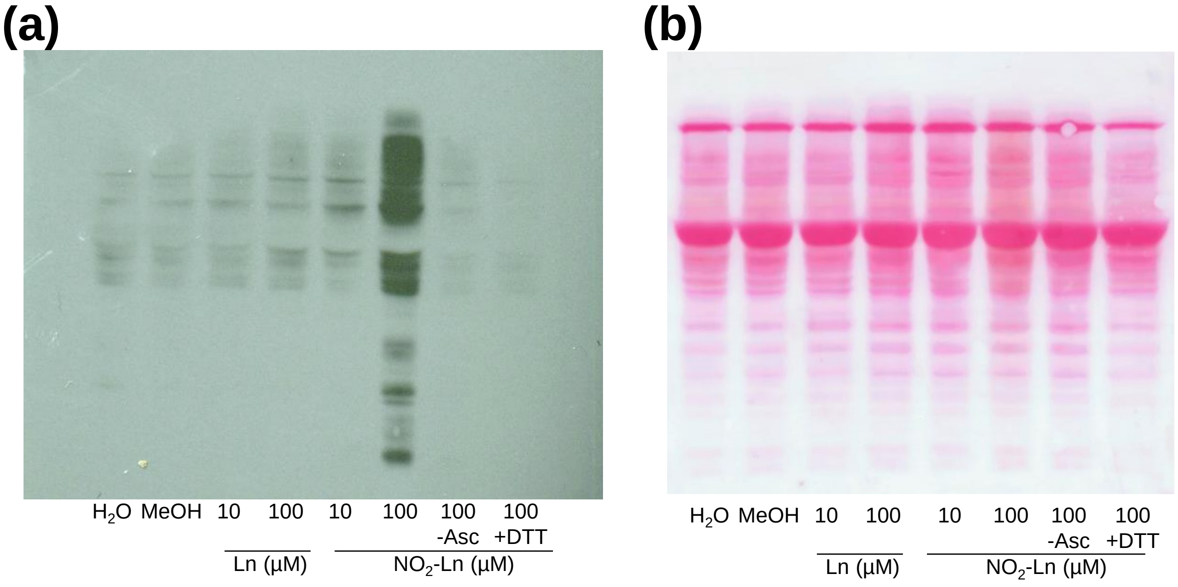

(a)
H_2O MeOH 10 100 10 100 100 -Asc 100 +DTT
Ln (µM)
NO_2-Ln (µM)
(b)
H_2O MeOH 10 100 10 100 100 -Asc 100 +DTT
Ln (µM)
NO_2-Ln (µM)

Supplementary Figure S4. Ascorbate and DTT specificity in the S-nitrosylation mediated by nitro-linolenic acid (NO_2-Ln). A) Protein extracts from the 14-day-old Arabidopsis plants were incubated with distilled water, methanol, 10 and 100 µM Ln and 10 and 100 µM NO_2-Ln. Then samples were subjected to the biotin switch technique (BST). As the negative controls, the extracts treated with 100 µM NO_2-Ln were subjected to the BST without ascorbate or were treated with Dithiothreitol (DTT) immediately after the S-nitrosylation process. **B)** Ponceau staining ensures equal protein loading (20 µg per line).

Supplementary Table S1. List of the protein-SNOs mediated by NO_2-Ln. The list contains the *S*-nitrosylated proteins identified by mass spectrometry approaches after incubating ACSCs with 100 µM NO_2-Ln (see Methods). ID mapping was performed by the UniProt database.

Table 1. List of specific protein-SNO mediated by NO_2-Ln

Protein ID	Entry Name	Protein names	Gene Names	Organism	Length
O03042	RBL_ARATH	Ribulose bisphosphate carboxylase large chain (R	rbcL AtCg00490	Arabidopsis thaliana (Mouse-ear cress)	479
Q9LYG3	MAOP2_ARATH	NADP-dependent malic enzyme 2 (AtNADP-ME2)	NADP-ME2 At5g11670 T22P22.60	Arabidopsis thaliana (Mouse-ear cress)	588
P55737	HS902_ARATH	Heat shock protein 90-2 (AtHSP90.2) (AtHsp90-2)	HSP90-2 ERD8 HSP81-2 LRA2 MUSE12 At5g56030 MD.	Arabidopsis thaliana (Mouse-ear cress)	699
O48646	GPX6_ARATH	Probable phospholipid hydroperoxide glutathione	GPX6 GPX1 At4g11600 T5C23.30	Arabidopsis thaliana (Mouse-ear cress)	232
O65639	CSP1_ARATH	Cold shock protein 1 (AtCSP1) (Cold shock domai	CSP1 CSDP1 At4g36020 T19K4.150	Arabidopsis thaliana (Mouse-ear cress)	299
Q9SAZ5	AHP3_ARATH	Histidine-containing phosphotransfer protein 3	AHP3 ATHP2 At5g39340 MUL8.2	Arabidopsis thaliana (Mouse-ear cress)	155
B3H7J4	B3H7J4_ARATH	Uncharacterized protein	AT3G58877	Arabidopsis thaliana (Mouse-ear cress)	59
Q8VYG2	GALAK_ARATH	Galacturonokinase (EC 2.7.1.44) (D-galacturonic a	GALAK At3g10700 F13M14.1 T7M13.22	Arabidopsis thaliana (Mouse-ear cress)	424
F4HWZ6	F4HWZ6_ARATH	Acyl carrier protein 3	ACP3 acyl carrier protein 3 At1g54630 AT1G54630 T22H	Arabidopsis thaliana (Mouse-ear cress)	107
O64517	MCA4_ARATH	Metacaspase-4 (AtMC4) (EC 3.4.22.-) (Metacaspa	AMC4 AMC7 MCP2D At1g79340 YUP8H12R.4	Arabidopsis thaliana (Mouse-ear cress)	418
Q0WT31	PMTP_ARATH	Probable methyltransferase PMT25 (EC 2.1.1.-)	At2g34300 F13P17.14	Arabidopsis thaliana (Mouse-ear cress)	770
F4IJ44	F4IJ44_ARATH	Calcium-binding EF hand family protein	TCH3 ATCAL4 calmodulin-like 12 CALMODULIN-RELATI	Arabidopsis thaliana (Mouse-ear cress)	289
Q9ZUU1	KAD1_ARATH	Adenylate kinase 1, chloroplastic (AK 1) (AtPADK	ADK At2g37250 F3G5.4	Arabidopsis thaliana (Mouse-ear cress)	284
Q9SEE5	GALK1_ARATH	Galactokinase (EC 2.7.1.6) (Galactose kinase)	GAL1 GALK At3g06580 F5E6.9	Arabidopsis thaliana (Mouse-ear cress)	496
Q42403	TRXH3_ARATH	Thioredoxin H3 (AtTrxh3) (Thioredoxin 3) (AtTRX3	TRX3 At5g42980 MBD2.18	Arabidopsis thaliana (Mouse-ear cress)	118
Q9LF98	ALFC8_ARATH	Fructose-bisphosphate aldolase 8, cytosolic (AtFB	FBA8 At3g52930 F8J2_100	Arabidopsis thaliana (Mouse-ear cress)	358
Q9LJJ7	AATP9_ARATH	AAA-ATPase At3g28580 (EC 3.6.1.-)	At3g28580 MZN14.5	Arabidopsis thaliana (Mouse-ear cress)	500
Q9FKS0	UKL1_ARATH	Uridine/cytidine kinase UKL1, chloroplastic (EC 2.	UKL1 UCK1 UK UPRT1 At5g40870 MHK7.10	Arabidopsis thaliana (Mouse-ear cress)	486
Q94C69	CSP3_ARATH	Cold shock domain-containing protein 3 (AtCSP3)	CSP3 At2g17870 T13L16.11	Arabidopsis thaliana (Mouse-ear cress)	301
Q9LFX8	Q9LFX8_ARATH	Glycine-rich protein (T7N9.15) (Uncharacterized p	AT1G27090 T7N9.15 T7N9_15	Arabidopsis thaliana (Mouse-ear cress)	420
F4I456	F4I456_ARATH	Tetratricopeptide repeat (TPR)-like superfamily pr	TPR2 AtTPR2 tetratricopeptide repeat 2 At1g04130 AT1	Arabidopsis thaliana (Mouse-ear cress)	360
Q93ZG7	RH38_ARATH	DEAD-box ATP-dependent RNA helicase 38 (EC	RH38 LOS4 At3g53110 T4D2.40	Arabidopsis thaliana (Mouse-ear cress)	496
Q39241	TRXH5_ARATH	Thioredoxin H5 (AtTrxh5) (Protein LOCUS OF INS	TRX5 LIV1 At1g45145 F27F5.21	Arabidopsis thaliana (Mouse-ear cress)	118
Q9LG23	PPR82_ARATH	Pentatricopeptide repeat-containing protein At1g5	At1g55890 F14J16.14	Arabidopsis thaliana (Mouse-ear cress)	398
Q8GY96	PGM_ARATH	Phosphoglycerate mutase-like protein (AtPGM)	PGM At2g17280 F5J6	Arabidopsis thaliana (Mouse-ear cress)	271
F4IZC8	F4IZC8_ARATH	Alpha-soluble NSF attachment protein 2	ALPHA-SNAP2 ASNAP At3g56190 AT3G56190	Arabidopsis thaliana (Mouse-ear cress)	240
F4JN17	F4JN17_ARATH	Ankyrin repeat-containing protein 2	AKR2 AFT AKR2A At4g35450 AT4G35450 F15J1.20 F1	Arabidopsis thaliana (Mouse-ear cress)	304
F4IQ61	F4IQ61_ARATH	Aconitase/3-isopropylmalate dehydratase protein	MFL8.15 IPMI SSU1 At2g43090 AT2G43090	Arabidopsis thaliana (Mouse-ear cress)	222
F4I035	F4I035_ARATH	3-phosphoshikimate 1-carboxyvinyltransferase (E	At1g48860 AT1G48860 T24P22.2	Arabidopsis thaliana (Mouse-ear cress)	489
A8MQW5	A8MQW5_ARATH	Cinnamoyl coa reductase 1	CCR1 ATCCR1 CINNAMOYL COA REDUCTASE cinnam	Arabidopsis thaliana (Mouse-ear cress)	337
Q8W457	OTU5_ARATH	OVARIAN TUMOR DOMAIN-containing deubiquiti	OTU5 At3g62940 T20O10.40	Arabidopsis thaliana (Mouse-ear cress)	332
O64650	RS271_ARATH	40S ribosomal protein S27-1	RPS27A ARS27C At2g45710 F17K2.35 F4I18.31	Arabidopsis thaliana (Mouse-ear cress)	84
Q9LHG9	NACA1_ARATH	Nascent polypeptide-associated complex subunit	At3g12390 T2E22.29	Arabidopsis thaliana (Mouse-ear cress)	203
Q9SUT4	Q9SUT4_ARATH	Transducin/WD40 repeat-like superfamily protein	At4g11270 AT4G11270 F8L21.60 F8L21_60	Arabidopsis thaliana (Mouse-ear cress)	1446
P42798	R15A1_ARATH	40S ribosomal protein S15a-1	RPS15AA At1g07770 F24B9.12; RPS15AF At5g59850 M	Arabidopsis thaliana (Mouse-ear cress)	130
Q39255	SKP1A_ARATH	SKP1-like protein 1A (SKP1-like 1) (UFO-binding	SKP1A ASK1 SKP1 UIP1 At1g75950 T4O12.17	Arabidopsis thaliana (Mouse-ear cress)	160
Q8H0X6	CYT6_ARATH	Cysteine proteinase inhibitor 6 (AtCYS-6) (PIP-M)	CYS6 At3g12490 MQC3.31 T2E22.19	Arabidopsis thaliana (Mouse-ear cress)	234
P59232	R27AB_ARATH	Ubiquitin-40S ribosomal protein S27a-2 [Cleaved	RPS27AB UBQ6 At2g47110 F14M4.6	Arabidopsis thaliana (Mouse-ear cress)	157
O82462	SYEC_ARATH	Glutamate--tRNA ligase, cytoplasmic (EC 6.1.1.17	At5g26710 F21E10.12	Arabidopsis thaliana (Mouse-ear cress)	719
P22954	HS702_ARATH	Heat shock 70 kDa protein 2 (Heat shock cognate	HSP70-2 HSC70-2 HSC70-G8 MED37_3 MED37D At5g0	Arabidopsis thaliana (Mouse-ear cress)	653
P49201	RS232_ARATH	40S ribosomal protein S23-2 (S12)	RPS23B At5g02960 F9G14_270	Arabidopsis thaliana (Mouse-ear cress)	142
P46309	GSH1_ARATH	Glutamate--cysteine ligase, chloroplastic (EC 6.3.	GSH1 CAD2 GCL PAD2 RML1 At4g23100 F7H19.290	Arabidopsis thaliana (Mouse-ear cress)	522
F4JK17	F4JK17_ARATH	Cofactor-independent phosphoglycerate mutase	At4g09520 AT4G09520 T15G18.60 T15G18_60	Arabidopsis thaliana (Mouse-ear cress)	492
Q8H1Q2	NBP35_ARATH	Cytosolic Fe-S cluster assembly factor NBP35 (Nu	NBP35 At5g50960 K3K7.12	Arabidopsis thaliana (Mouse-ear cress)	350
F4IAR7	TCPE_ARATH	T-complex protein 1 subunit epsilon (TCP-1-epsilo	CCT5 At1g24510 F21J9.12	Arabidopsis thaliana (Mouse-ear cress)	535
Q8L7N0	TCPZB_ARATH	T-complex protein 1 subunit zeta 2 (TCP-1-zeta 2)	CCT6B At5g16070 F1N13.210	Arabidopsis thaliana (Mouse-ear cress)	535
Q9FVI6	FWA_ARATH	Homeobox-leucine zipper protein HDG6 (HD-ZIP	HDG6 FWA HDGL2-6 At4g25530 M7J2.100	Arabidopsis thaliana (Mouse-ear cress)	686
Q8RXF8	MIRO1_ARATH	Mitochondrial Rho GTPase 1 (AtMIRO1) (EC 3.6.5	MIRO1 At5g27540	Arabidopsis thaliana (Mouse-ear cress)	648
P51818	HS903_ARATH	Heat shock protein 90-3 (AtHSP90.3) (AtHsp90-3)	HSP90-3 HSP81-3 HSP81.2 MUSE10 At5g56010 MDA7.	Arabidopsis thaliana (Mouse-ear cress)	699
P0DH95	CALM1_ARATH	Calmodulin-1 (CaM-1)	CAM1 At5g37780 K22F20.2 T31G3.3	Arabidopsis thaliana (Mouse-ear cress)	149
F4HU93	F4HU93_ARATH	L-ascorbate peroxidase (EC 1.11.1.11)	APX1 ASCORBATE PEROXIDASE ascorbate peroxidase	Arabidopsis thaliana (Mouse-ear cress)	249
F4IWK4	F4IWK4_ARATH	Sucrase/ferredoxin-like family protein	At3g27570 AT3G27570	Arabidopsis thaliana (Mouse-ear cress)	379
Q94K48	Q94K48_ARATH	ARM repeat superfamily protein (Uncharacterized	At3g62530 AT3G62530	Arabidopsis thaliana (Mouse-ear cress)	221
Q9LZ23	ACR12_ARATH	ACT domain-containing protein ACR12 (Protein A	ACR12 At5g04740 MUK11.6 T1E3.100	Arabidopsis thaliana (Mouse-ear cress)	301
P23686	METK1_ARATH	S-adenosylmethionine synthase 1 (AdoMet syntha	SAM1 At1g02500 T14P4.17 T14P4_22	Arabidopsis thaliana (Mouse-ear cress)	393
Q9C829	NU50A_ARATH	Nuclear pore complex protein NUP50A (Nucleopo	NUP50A At1g52380 F19K6.4	Arabidopsis thaliana (Mouse-ear cress)	440
Q9SUT5	SGT1B_ARATH	Protein SGT1 homolog B (AtSGT1b) (Protein ENH	SGT1B EDM1 ETA3 RPR1 At4g11260 F8L21.50	Arabidopsis thaliana (Mouse-ear cress)	358
O22165	RLP24_ARATH	Probable ribosome biogenesis protein RLP24	At2g44860 T13E15.13	Arabidopsis thaliana (Mouse-ear cress)	159
O23722	MVD1_ARATH	Diphosphomevalonate decarboxylase MVD1, pero	MVD1 MDD1 MVD At2g38700	Arabidopsis thaliana (Mouse-ear cress)	412
Q84WW2	6PGL3_ARATH	6-phosphogluconolactonase 3, chloroplastic (6PG	PGL3 EMB2024 At5g24400 K16H17.11	Arabidopsis thaliana (Mouse-ear cress)	325
Q9SRG3	PDI12_ARATH	Protein disulfide isomerase-like 1-2 (AtPDIL1-2) (E	PDIL1-2 PDI6 At1g77510 T5M16.10	Arabidopsis thaliana (Mouse-ear cress)	508
Q39219	AOX1A_ARATH	Ubiquinol oxidase 1a, mitochondrial (EC 1.10.3.11	AOX1A AOX1 HSR3 At3g22370 MCB17.11	Arabidopsis thaliana (Mouse-ear cress)	354
P10896	RCA_ARATH	Ribulose bisphosphate carboxylase/oxygenase ac	RCA At2g39730 T5I7.3	Arabidopsis thaliana (Mouse-ear cress)	474
Q8S4F6	SQD2_ARATH	Sulfoquinovosyl transferase SQD2 (EC 2.4.1.-) (P	SQD2 At5g01220 F7J8.200	Arabidopsis thaliana (Mouse-ear cress)	510
B3H5V4	B3H5V4_ARATH	Ubiquitin carboxyl-terminal hydrolase (EC 3.4.19.1	UBP6 ATUBP6 UBIQUITIN-SPECIFIC PROTEASE 6 ubi	Arabidopsis thaliana (Mouse-ear cress)	443
Q9LD55	EIF3A_ARATH	Eukaryotic translation initiation factor 3 subunit A (	TIF3A1 At4g11420 F25E4.40	Arabidopsis thaliana (Mouse-ear cress)	987
F4JIZ3	F4JIZ3_ARATH	Phospho-2-dehydro-3-deoxyheptonate aldolase (E	DHS2 AtDAHP2 DAHP2 At4g33510 AT4G33510 F17M5.	Arabidopsis thaliana (Mouse-ear cress)	347
Q9LT02	PDR2_ARATH	Probable manganese-transporting ATPase PDR2	PDR2 MIA At5g23630 MQM1.11	Arabidopsis thaliana (Mouse-ear cress)	1179
Q9FGH3	Q9FGH3_ARATH	Cinnamoyl-CoA reductase-like protein (Dihydrofla	MQJ2.6 MQJ2_6 AT5G58490	Arabidopsis thaliana (Mouse-ear cress)	324
Q9SHC8	VAP12_ARATH	Vesicle-associated protein 1-2 (Plant VAP homolo	PVA12 VAP12 At2g45140 T14P1.5	Arabidopsis thaliana (Mouse-ear cress)	239
P41377	IF4A2_ARATH	Eukaryotic initiation factor 4A-2 (eIF-4A-2) (EC 3.	TIF4A-2 RH19 At1g54270 F20D21.9 F20D21_52	Arabidopsis thaliana (Mouse-ear cress)	412
Q9S7C0	HSP7O_ARATH	Heat shock 70 kDa protein 14 (Heat shock protein	HSP70-14 HSP91 At1g79930 F18B13.1 F19K16.11	Arabidopsis thaliana (Mouse-ear cress)	831
Q9FMU6	MPCP3_ARATH	Mitochondrial phosphate carrier protein 3, mitocho	MPT3 AT5 PHT3;1 At5g14040 MUA22_4	Arabidopsis thaliana (Mouse-ear cress)	375
Q8VZ31	Q8VZ31_ARATH	Tetratricopeptide repeat (TPR)-like superfamily pr	TPR4 tetratricopeptide repeat 4 At1g04530 AT1G04530	Arabidopsis thaliana (Mouse-ear cress)	310
Q9LFG2	DAPF_ARATH	Diaminopimelate epimerase, chloroplastic (DAP e	DAPF At3g53580 F4P12.280	Arabidopsis thaliana (Mouse-ear cress)	362
Q9C9M1	Q9C9M1_ARATH	Protein-lysine N-methyltransferase At1g66680 (E	AR401 At1g66680 AT1G66680 F4N21.18 F4N21_18	Arabidopsis thaliana (Mouse-ear cress)	358
Q39054	CNX1_ARATH	Molybdopterin biosynthesis protein CNX1 (Molybd	CNX1 At5g20990 F22D1.6 T10F18.20	Arabidopsis thaliana (Mouse-ear cress)	670
O81148	PSA4A_ARATH	Proteasome subunit alpha type-4-A (20S proteas	PAC1 PRC9 PRS1 At3g22110 MKA23.2	Arabidopsis thaliana (Mouse-ear cress)	250
Q9M214	Q9M214_ARATH	At3g60450 (Phosphoglycerate mutase family prot	T8B10_110 AT3G60450	Arabidopsis thaliana (Mouse-ear cress)	274
O22809	O22809_ARATH	NAD(P)-binding Rossmann-fold superfamily prote	CRL1 AtCRL1 At2g33590 AT2G33590 F4P9.36 F4P9_3	Arabidopsis thaliana (Mouse-ear cress)	321
Q8VZ95	VAP11_ARATH	Vesicle-associated protein 1-1 (Plant VAP homolo	PVA11 VAP27 VAP27-1 At3g60600 T4C21.10	Arabidopsis thaliana (Mouse-ear cress)	256
P11574	VATB1_ARATH	V-type proton ATPase subunit B1 (V-ATPase subu	VHA-B1 AT57 At1g76030 T4O12.24	Arabidopsis thaliana (Mouse-ear cress)	486
Q42064	RL83_ARATH	60S ribosomal protein L8-3	RPL8C At4g36130 F23E13.20 T19K4.260	Arabidopsis thaliana (Mouse-ear cress)	258
Q9M7T0	PRX2F_ARATH	Peroxiredoxin-2F, mitochondrial (EC 1.11.1.25) (G	PRXIIF At3g06050 F24F17.3	Arabidopsis thaliana (Mouse-ear cress)	201
Q94AR8	LEUC_ARATH	3-isopropylmalate dehydratase large subunit, chlo	IIL1 At4g13430 T9E8.170	Arabidopsis thaliana (Mouse-ear cress)	509
Q9FLT0	TSN2_ARATH	Ribonuclease TUDOR 2 (AtTudor2) (TUDOR-SN	TSN2 At5g61780 MAC9.10	Arabidopsis thaliana (Mouse-ear cress)	985
Q93XW5	NSP5_ARATH	Nitrile-specifier protein 5 (AtNSP5)	NSP5 At5g48180 MIF21.7	Arabidopsis thaliana (Mouse-ear cress)	326
A8MRZ7	A8MRZ7_ARATH	Eukaryotic translation initiation factor 4A1	EIF4A1 RH4 TIF4A1 At3g13920 AT3G13920	Arabidopsis thaliana (Mouse-ear cress)	402
Q9LSV0	GLYR1_ARATH	Glyoxylate/succinic semialdehyde reductase 1 (At	GLYR1 GR1 At3g25530 MWL2.18	Arabidopsis thaliana (Mouse-ear cress)	289
Q9SCN8	CD48D_ARATH	Cell division control protein 48 homolog D (AtCDC	CDC48D At3g53230 T4D2.160	Arabidopsis thaliana (Mouse-ear cress)	815
Q9M9W1	RL222_ARATH	60S ribosomal protein L22-2	RPL22B At3g05560 F18C1.17	Arabidopsis thaliana (Mouse-ear cress)	124
Q9LQQ3	Q9LQQ3_ARATH	At1g07750/F24B9_13 (F24B9.13 protein) (Putativ	At1g07750 AT1G07750 F24B9.13 F24B9_13	Arabidopsis thaliana (Mouse-ear cress)	356
Q9SI58	Q9SI58_ARATH	60S ribosomal export protein NMD3	NMD3 AtNMD3 Nonsense-Mediated mRNA Decay 3 At2g	Arabidopsis thaliana (Mouse-ear cress)	516
Q9M2X8	Q9M2X8_ARATH	AT3g49800/T16K5_150 (BSD domain-containing	T16K5.150 At3g49800 AT3G49800	Arabidopsis thaliana (Mouse-ear cress)	428
Q9XES1	ECA4_ARATH	Calcium-transporting ATPase 4, endoplasmic retic	ECA4 At1g07670 F24B9.24	Arabidopsis thaliana (Mouse-ear cress)	1061
A4GNA8	PSD3_ARATH	Phosphatidylserine decarboxylase proenzyme 3 (	PSD3 At4g25970 F20B18.80	Arabidopsis thaliana (Mouse-ear cress)	635
Q8LPL6	AP2A1_ARATH	AP-2 complex subunit alpha-1 (Adaptor protein co	ALPHA-ADR At5g22770 MDJ22.19	Arabidopsis thaliana (Mouse-ear cress)	1012
Q9LUT2	METK4_ARATH	S-adenosylmethionine synthase 4 (AdoMet syntha	METK4 MTO3 SAMS3 At3g17390 MGD8.23	Arabidopsis thaliana (Mouse-ear cress)	393
F4HQD4	HSP7P_ARATH	Heat shock 70 kDa protein 15 (Heat shock protein	HSP70-15 At1g79920 F19K16.12	Arabidopsis thaliana (Mouse-ear cress)	831
Q42586	UMPS_ARATH	Uridine 5'-monophosphate synthase (UMP syntha	PYRE-F UMPS At3g54470 T14E10.40	Arabidopsis thaliana (Mouse-ear cress)	476
Q8H104	SYDC1_ARATH	Aspartate--tRNA ligase 1, cytoplasmic (EC 6.1.1.1	At4g26870 F10M23.210	Arabidopsis thaliana (Mouse-ear cress)	532
Q9LK57	PP226_ARATH	Pentatricopeptide repeat-containing protein At3g1	At3g13160 MJG19.11	Arabidopsis thaliana (Mouse-ear cress)	394
Q9M1W4	HMT2_ARATH	Homocysteine S-methyltransferase 2 (EC 2.1.1.1	HMT-2 At3g63250 F16M2.100	Arabidopsis thaliana (Mouse-ear cress)	333
Q9ZVI9	PECT1_ARATH	Ethanolamine-phosphate cytidylyltransferase (EC	PECT1 At2g38670 T6A23.13	Arabidopsis thaliana (Mouse-ear cress)	421
Q9LK34	UKL2_ARATH	Uridine/cytidine kinase UKL1, chloroplastic (EC 2.	UKL2 UCK2 At3g27190 K17E12.1	Arabidopsis thaliana (Mouse-ear cress)	483
F4J7Z8	F4J7Z8_ARATH	E3 UFM1-protein ligase-like protein	At3g46220 AT3G46220	Arabidopsis thaliana (Mouse-ear cress)	788

Q9SVN5	SYMC_ARATH	Methionine--tRNA ligase, cytoplasmic (EC 6.1.1.1(	At4g13780 F18A5.170	Arabidopsis thaliana (Mouse-ear cress)	797
Q8H0T4	UPL2_ARATH	E3 ubiquitin-protein ligase UPL2 (Ubiquitin-protein	UPL2 At1g70320 F17O7.14/F17O7.15	Arabidopsis thaliana (Mouse-ear cress)	3658
P54609	CD48A_ARATH	Cell division control protein 48 homolog A (AtCDC	CDC48A CDC48 At3g09840 F8A24.11	Arabidopsis thaliana (Mouse-ear cress)	809
O49543	MNIF1_ARATH	Cysteine desulfurase, mitochondrial (EC 2.8.1.7) (	NIFS1 MtNIFS1 NIFS NSF1 At5g65720 F6H11.150 MPA2	Arabidopsis thaliana (Mouse-ear cress)	453
Q8S528	AL2B7_ARATH	Aldehyde dehydrogenase family 2 member B7, mi	ALDH2B7 ALDH3 At1g23800 F5O8.33 F5O8.35	Arabidopsis thaliana (Mouse-ear cress)	534
Q38931	FKB62_ARATH	Peptidyl-prolyl cis-trans isomerase FKBP62 (PPIas	FKBP62 ROF1 At3g25230 MJL12.19	Arabidopsis thaliana (Mouse-ear cress)	551
P53494	ACT4_ARATH	Actin-4 (EC 3.6.4.-)	ACT4 At5g59370 F2O15.3	Arabidopsis thaliana (Mouse-ear cress)	377
Q93V62	Q93V62_ARATH	AT4g27450/F27G19_50 (Aluminum induced prote	AT4G27450 F27G19.50 F27G19_50	Arabidopsis thaliana (Mouse-ear cress)	250
F4I7M5	F4I7M5_ARATH	Spermidine synthase	SPDS1 spermidine synthase 1 At1g23820 AT1G23820 F!	Arabidopsis thaliana (Mouse-ear cress)	327
Q9SIZ2	IF2AH_ARATH	Eukaryotic translation initiation factor 2 subunit alp	At2g40290	Arabidopsis thaliana (Mouse-ear cress)	344
Q9ZSK4	ADF3_ARATH	Actin-depolymerizing factor 3 (ADF-3) (AtADF3)	ADF3 At5g59880 MMN10.12	Arabidopsis thaliana (Mouse-ear cress)	139
P31414	AVP1_ARATH	Pyrophosphate-energized vacuolar membrane pro	AVP1 AVP AVP-3 AVP3 At1g15690 F7H2.3	Arabidopsis thaliana (Mouse-ear cress)	770
O24633	PSB2B_ARATH	Proteasome subunit beta type-2-B (20S proteasor	PBD2 PRCGA At4g14800 dl3440w FCAALL.135	Arabidopsis thaliana (Mouse-ear cress)	199
F4I2A1	F4I2A1_ARATH	Prolyl endopeptidase (EC 3.4.21.-)	At1g76140 AT1G76140 T23E18.8 T23E18_8	Arabidopsis thaliana (Mouse-ear cress)	792
O24466	RAE1A_ARATH	Ras-related protein RABE1a (AtRABE1a) (Ras-rel	RABE1A RAB8 RAB8B At3g53610 F4P12.310	Arabidopsis thaliana (Mouse-ear cress)	216
Q940P8	TCPB_ARATH	T-complex protein 1 subunit beta (TCP-1-beta) (Cc	CCT2 At5g20890 F22D1.60	Arabidopsis thaliana (Mouse-ear cress)	527
Q9FI56	CLPC1_ARATH	Chaperone protein ClpC1, chloroplastic (ATP-depe	CLPC1 DCA1 HSP93-V IRM1 At5g50920 K3K7.7	Arabidopsis thaliana (Mouse-ear cress)	929
Q9SI61	ASE1_ARATH	Amidophosphoribosyltransferase 1, chloroplastic (	ASE1 GPRAT1 At2g16570 F1P15	Arabidopsis thaliana (Mouse-ear cress)	566
Q9ZPY7	XPO2_ARATH	Exportin-2 (Exp2) (Cellular apoptosis susceptibility	CAS At2g46520 F11C10 F13A10.5	Arabidopsis thaliana (Mouse-ear cress)	972
Q8GUN2	HINT1_ARATH	Adenylylsulfatase HINT1 (EC 3.6.2.1) (HIS triad fa	HINT1 HIT3 At3g56490 5P19_140	Arabidopsis thaliana (Mouse-ear cress)	147
P17562	METK2_ARATH	S-adenosylmethionine synthase 2 (AdoMet syntha	SAM2 At4g01850 T7B11.11	Arabidopsis thaliana (Mouse-ear cress)	393
P29511	TBA6_ARATH	Tubulin alpha-6 chain (EC 3.6.5.-)	TUBA6 TUA6 At4g14960 dl3520c	Arabidopsis thaliana (Mouse-ear cress)	450
O81062	SIP_ARATH	Signal peptide peptidase (AtSPP) (EC 3.4.23.-) (Ir	SPP At2g03120 T18E12.21	Arabidopsis thaliana (Mouse-ear cress)	344
Q94K05	TCPQ_ARATH	T-complex protein 1 subunit theta (TCP-1-theta) ((	CCT8 TRIC At3g03960 T11I18.7	Arabidopsis thaliana (Mouse-ear cress)	549
Q9FKI2	Q9FKI2_ARATH	Eukaryotic translation initiation factor 4C	MXH1.2 MXH1_2 At5g35680 AT5G35680	Arabidopsis thaliana (Mouse-ear cress)	145
Q9LMI0	TPS7_ARATH	Probable alpha,alpha-trehalose-phosphate syntha	TPS7 TPSA At1g06410 T2D23.11	Arabidopsis thaliana (Mouse-ear cress)	851
P27323	HS901_ARATH	Heat shock protein 90-1 (AtHSP90.1) (AtHsp90-1)	HSP90-1 HSP81-1 HSP83 At5g52640 F6N7.13	Arabidopsis thaliana (Mouse-ear cress)	700
P93033	FUM1_ARATH	Fumarate hydratase 1, mitochondrial (AtFUM1) (F	FUM1 At2g47510 T30B22.19	Arabidopsis thaliana (Mouse-ear cress)	492
O80565	OEP37_ARATH	Outer envelope pore protein 37, chloroplastic (Chl	OEP37 At2g43950 F6E13.8	Arabidopsis thaliana (Mouse-ear cress)	343
Q84WV1	TCPG_ARATH	T-complex protein 1 subunit gamma (TCP-1-gamn	CCT3 At5g26360 F9D12.18	Arabidopsis thaliana (Mouse-ear cress)	555
Q42601	CARB_ARATH	Carbamoyl-phosphate synthase large chain, chlor	CARB VEN3 At1g29900 F1N18.6	Arabidopsis thaliana (Mouse-ear cress)	1187
Q9SW96	SYNC1_ARATH	Asparagine--tRNA ligase, cytoplasmic 1 (EC 6.1.1	SYNC1 EMB2755 At5g56680 MIK19.13	Arabidopsis thaliana (Mouse-ear cress)	572
P53492	ACT7_ARATH	Actin-7 (EC 3.6.4.-) (Actin-2)	ACT7 At5g09810 MYH9.2	Arabidopsis thaliana (Mouse-ear cress)	377
Q96292	ACT2_ARATH	Actin-2 (EC 3.6.4.-)	ACT2 At3g18780 MVE11.16	Arabidopsis thaliana (Mouse-ear cress)	377
A8MRG9	A8MRG9_ARATH	P-loop containing nucleoside triphosphate hydrola	ABCI21 ATNAP2 ATP-binding cassette A21 ATPOP1 ML	Arabidopsis thaliana (Mouse-ear cress)	223
Q9SGW3	PSD8A_ARATH	26S proteasome non-ATPase regulatory subunit 8	RPN12A At1g64520 F1N19.9	Arabidopsis thaliana (Mouse-ear cress)	267
Q9FY49	LKHA4_ARATH	Leucine aminopeptidase (EC 3.4.11.-) (Epoxide hy	LKHA4 At5g13520 T6I14_50	Arabidopsis thaliana (Mouse-ear cress)	616
Q9LV21	TCPD_ARATH	T-complex protein 1 subunit delta (TCP-1-delta) (C	CCT4 At3g18190 MRC8	Arabidopsis thaliana (Mouse-ear cress)	536
P17094	RL31_ARATH	60S ribosomal protein L3-1 (Protein EMBRYO DEI	ARP1 EMB2207 RPL3A At1g43170 F1I21.1	Arabidopsis thaliana (Mouse-ear cress)	389
Q42406	CP18D_ARATH	Peptidyl-prolyl cis-trans isomerase CYP18-4 (PPIa	CYP18-4 43H1 CYP1 CYPA ROC5 At4g34870 T11I11.11	Arabidopsis thaliana (Mouse-ear cress)	172
Q9LKR8	RAF1_ARATH	Rubisco accumulation factor 1.1, chloroplastic	RAF1.1 At5g28500 T26D3.4	Arabidopsis thaliana (Mouse-ear cress)	434
F4J4J3	EBP1_ARATH	ERBB-3 BINDING PROTEIN 1 (AtEBP1) (Prolifera	EBP1 CAM1 CPR At3g51800	Arabidopsis thaliana (Mouse-ear cress)	392
F4IXW2	BIG5_ARATH	Brefeldin A-inhibited guanine nucleotide-exchange	BIG5 BEN1 MIN7 At3g43300 F7K15_150	Arabidopsis thaliana (Mouse-ear cress)	1739
P42770	GSHRP_ARATH	Glutathione reductase, chloroplastic (GR) (GRase	EMB2360 At3g54660 T5N23_20	Arabidopsis thaliana (Mouse-ear cress)	565
F4HRW5	F4HRW5_ARATH	Ribosomal protein L22p/L17e family protein	At1g67430 AT1G67430 T1F15.11 T1F15_11	Arabidopsis thaliana (Mouse-ear cress)	131
O23712	PSA1B_ARATH	Proteasome subunit alpha type-1-B (20S proteasc	PAF2 PRC2B PRS1 At1g47250 F8G22.3	Arabidopsis thaliana (Mouse-ear cress)	277
Q9S9P1	RS121_ARATH	40S ribosomal protein S12-1	RPS12A At1g15930 T24D18.3	Arabidopsis thaliana (Mouse-ear cress)	144
P17745	EFTU_ARATH	Elongation factor Tu, chloroplastic (EF-Tu) (Ras-re	TUFA RAB8D RABE1B At4g20360 F9F13.10	Arabidopsis thaliana (Mouse-ear cress)	476
O80763	NRX1_ARATH	Probable nucleoredoxin 1 (AtNrx1) (EC 1.8.1.8)	At1g60420 T13D8.29	Arabidopsis thaliana (Mouse-ear cress)	578
Q9SGT7	Q9SGT7_ARATH	At1g56110/T6H22_9 (NOP56-like pre RNA proces	NOP56 NOP56-LIKE PROTEIN AT1G56110 T6H22.10 T(	Arabidopsis thaliana (Mouse-ear cress)	522
O24456	GBLPA_ARATH	Receptor for activated C kinase 1A (Guanine nucl	RACK1A ARCA At1g18080 T10F20.9 T10O22.6	Arabidopsis thaliana (Mouse-ear cress)	327
Q94BQ2	PRS8B_ARATH	26S proteasome regulatory subunit 8 homolog B (	RPT6B SUG1 At5g20000 F28I16.150	Arabidopsis thaliana (Mouse-ear cress)	419
Q9LTF2	RS103_ARATH	40S ribosomal protein S10-3	RPS10C At5g52650 F6N7.14	Arabidopsis thaliana (Mouse-ear cress)	179
B3H4B6	B3H4B6_ARATH	40S ribosomal protein S25	At4g39200 AT4G39200 T22F8.100 T22F8_100	Arabidopsis thaliana (Mouse-ear cress)	107
P93042	RHD3_ARATH	Protein ROOT HAIR DEFECTIVE 3 (EC 3.6.5.-) (F	RHD3 FRA4 At3g13870 MCP4.11	Arabidopsis thaliana (Mouse-ear cress)	802
Q9LF33	UGDH3_ARATH	UDP-glucose 6-dehydrogenase 3 (UDP-Glc dehyc	UGD3 At5g15490 T20K14_100	Arabidopsis thaliana (Mouse-ear cress)	480
Q9SYP2	PFPA1_ARATH	Pyrophosphate--fructose 6-phosphate 1-phosphot	PFP-ALPHA1 At1g20950 F9H16.6	Arabidopsis thaliana (Mouse-ear cress)	614
O04630	SYTM1_ARATH	Threonine--tRNA ligase, mitochondrial 1 (EC 6.1.1	THRRS At5g26830 F2P16.7	Arabidopsis thaliana (Mouse-ear cress)	709
Q9FRL8	DHAR2_ARATH	Glutathione S-transferase DHAR2 (EC 2.5.1.18) ((	DHAR2 DHAR At1g75270 F22H5.1	Arabidopsis thaliana (Mouse-ear cress)	213
Q8VZC7	DRL36_ARATH	Probable disease resistance protein At5g45510	At5g45510 MFC19.18	Arabidopsis thaliana (Mouse-ear cress)	1222
Q94A52	RH2_ARATH	Eukaryotic initiation factor 4A-III homolog (AteIF4A	EIF4A3 RH2 At3g19760 MMB12.25 MMB12.4	Arabidopsis thaliana (Mouse-ear cress)	408
O82647	PDC1_ARATH	Pyruvate decarboxylase 1 (AtPDC1) (EC 4.1.1.1)	PDC1 At4g33070 F4I10.4	Arabidopsis thaliana (Mouse-ear cress)	607
Q8H126	Q8H126_ARATH	Selenoprotein O	AT5G13040 At5g13030 AT5G13030 T19L5.1	Arabidopsis thaliana (Mouse-ear cress)	633
Q9SF85	ADK1_ARATH	Adenosine kinase 1 (AK 1) (EC 2.7.1.20) (Adenos	ADK1 At3g09820 F8A24.13	Arabidopsis thaliana (Mouse-ear cress)	344
Q9CAI3	CADH1_ARATH	Probable cinnamyl alcohol dehydrogenase 1 (AtC	CAD1 CADG At1g72680 F28P22.13	Arabidopsis thaliana (Mouse-ear cress)	355
F4IU89	HS905_ARATH	Heat shock protein 90-5, chloroplastic (AtHSP90.5	HSP90-5 CR88 EMB1956 HSP88-1 At2g04030	Arabidopsis thaliana (Mouse-ear cress)	780
P59230	R10A2_ARATH	60S ribosomal protein L10a-2	RPL10AB At2g27530 F10A12.22 F15K20.37	Arabidopsis thaliana (Mouse-ear cress)	216
Q9LQ04	RMLCD_ARATH	Bifunctional dTDP-4-dehydrorhamnose 3,5-epime	NRS/ER At1g63000 F16P17.17	Arabidopsis thaliana (Mouse-ear cress)	301
F4J447	F4J447_ARATH	Alpha/beta-Hydrolases superfamily protein	At3g23600 AT3G23600	Arabidopsis thaliana (Mouse-ear cress)	236
Q9ASR1	EF2_ARATH	Elongation factor 2 (EF-2) (Protein LOW EXPRES	LOS1 At1g56070/At1g56075 T6H22.13	Arabidopsis thaliana (Mouse-ear cress)	843
Q9SV20	COPB2_ARATH	Coatomer subunit beta-2 (Beta-coat protein 2) (Be	At4g31490 F3L17.60	Arabidopsis thaliana (Mouse-ear cress)	948
Q9SPK5	FTHS_ARATH	Formate--tetrahydrofolate ligase (EC 6.3.4.3) (10-f	THFS At1g50480 F11F12.17 F17J6.2	Arabidopsis thaliana (Mouse-ear cress)	634
F4J7I2	F4J7I2_ARATH	ubiquitinyl hydrolase 1 (EC 3.4.19.12)	UBP13 AtUBP13 ubiquitin-specific protease 13 AT3G119	Arabidopsis thaliana (Mouse-ear cress)	1114
F4K9K4	F4K9K4_ARATH	Syntaxin of plants 132	SYP132 ATSYP132 syntaxin of plants 132 At5g08080 AT	Arabidopsis thaliana (Mouse-ear cress)	223
F4K1Y4	NTF2_ARATH	Nuclear transport factor 2 (AtNTF2)	NTF2 At5g60980 MSL3.12	Arabidopsis thaliana (Mouse-ear cress)	460
P93655	LONM1_ARATH	Lon protease homolog 1, mitochondrial (EC 3.4.21	LON1 At5g26860 F2P16.23	Arabidopsis thaliana (Mouse-ear cress)	940
Q56W28	Q56W28_ARATH	Glutamate decarboxylase (EC 4.1.1.15)	GAD2 GLUTAMATE DECARBOXYLASE 2 glutamate dec	Arabidopsis thaliana (Mouse-ear cress)	365
Q9LSP5	Q9LSP5_ARATH	AT3g17020/K14A17_14 (Adenine nucleotide alph	AT3G17020	Arabidopsis thaliana (Mouse-ear cress)	163
P42794	RL112_ARATH	60S ribosomal protein L11-2 (L16)	RPL11B At3g58700 T20N10_50; RPL11C RPL16B At4g1	Arabidopsis thaliana (Mouse-ear cress)	182
Q8H107	ODO2B_ARATH	Dihydrolipoyllysine-residue succinyltransferase co	At4g26910 F10M23.250	Arabidopsis thaliana (Mouse-ear cress)	464
O65902	ACAP1_ARATH	Cyclase-associated protein 1 (AtCAP1) (Adenylyl (	CAP1 At4g34490 T4L20.70	Arabidopsis thaliana (Mouse-ear cress)	476
Q42262	RS3A2_ARATH	40S ribosomal protein S3a-2	RPS3AB CYC07 At4g34670 T4L20.250	Arabidopsis thaliana (Mouse-ear cress)	262
P42036	RS143_ARATH	40S ribosomal protein S14-3	RPS14C At3g52580 F22O6_40 F3C22.6	Arabidopsis thaliana (Mouse-ear cress)	150
P29514	TBB6_ARATH	Tubulin beta-6 chain (Beta-6-tubulin)	TUBB6 TUB6 At5g12250 MXC9.21	Arabidopsis thaliana (Mouse-ear cress)	449
B5X564	CDC2C_ARATH	Cyclin-dependent kinase C-2 C (CDC2CAt) (Prote	CDC2C CKL1 At5g39420 MUL8.100	Arabidopsis thaliana (Mouse-ear cress)	644
Q9FFP6	Q9FFP6_ARATH	Pyruvate kinase (EC 2.7.1.40)	MBK5.16 MBK5_16 At5g63680 AT5G63680	Arabidopsis thaliana (Mouse-ear cress)	510
Q9LIA8	UGDH2_ARATH	UDP-glucose 6-dehydrogenase 2 (UDP-Glc dehyc	UGD2 UGD At3g29360 MUO10.18 MUO10_6	Arabidopsis thaliana (Mouse-ear cress)	480
Q03251	RBG8_ARATH	Glycine-rich RNA-binding protein 8 (AtGR-RBP8) (	RBG8 CCR1 GR-RBP8 GRP8 SRBP2 At4g39260 T22F8.	Arabidopsis thaliana (Mouse-ear cress)	169
B3H778	B3H778_ARATH	argininosuccinate synthase (EC 6.3.4.5) (Citrulline	At4g24830 AT4G24830 F6I7.40 F6I7_40	Arabidopsis thaliana (Mouse-ear cress)	450
Q9LIL3	Q9LIL3_ARATH	Aluminum induced protein with YGL and LRDR mo	At3g22850 AT3G22850	Arabidopsis thaliana (Mouse-ear cress)	248
Q9M339	RS32_ARATH	40S ribosomal protein S3-2	RPS3B At3g53870 F5K20_170	Arabidopsis thaliana (Mouse-ear cress)	249
F4I4Z2	F4I4Z2_ARATH	Alanine--tRNA ligase (EC 6.1.1.7) (Alanyl-tRNA sy	ALATS ACD Alanyl-tRNA synthetase ALARS At1g50200	Arabidopsis thaliana (Mouse-ear cress)	1011
Q9LR75	HEM61_ARATH	Coproporphyrinogen-III oxidase 1, chloroplastic (A	CPX1 CPO HEMF HEMF1 LIN2 At1g03475 F21B7.10	Arabidopsis thaliana (Mouse-ear cress)	386
P11139	TBA1_ARATH	Tubulin alpha-1 chain (EC 3.6.5.-)	TUBA1 TUA1 At1g64740 F13O11.5	Arabidopsis thaliana (Mouse-ear cress)	450
F4I7I0	ALAT1_ARATH	Alanine aminotransferase 1, mitochondrial (AtAlaA	ALAAT1 AOAT4 At1g17290 T13M22.3	Arabidopsis thaliana (Mouse-ear cress)	543
A8MR47	A8MR47_ARATH	Sulfurtransferase	MST1 ATMST1 ATRDH1 ST1 STR1 SULFURTRANSFER	Arabidopsis thaliana (Mouse-ear cress)	282
P0CJ46	ACT1_ARATH	Actin-1 (EC 3.6.4.-)	ACT1 AAC1 At2g37620 F13M22.12	Arabidopsis thaliana (Mouse-ear cress)	377
Q9S7W4	Q9S7W4_ARATH	AT3g07720/F17A17_6 (F17A17.6 protein) (Galact	F17A17.6 MLP3.17 At3g07720 AT3G07720	Arabidopsis thaliana (Mouse-ear cress)	329
F4I454	F4I454_ARATH	ABC-type xenobiotic transporter (EC 7.6.2.2)	ABCC5 ATABCC5 ATMRP5 ATP-binding cassette C5 ML	Arabidopsis thaliana (Mouse-ear cress)	1509
Q8H1Y0	ODPA2_ARATH	Pyruvate dehydrogenase E1 component subunit a	IAR4 At1g24180 F3I6.11	Arabidopsis thaliana (Mouse-ear cress)	393
Q38845	2AAA_ARATH	Serine/threonine-protein phosphatase 2A 65 kDa	PP2AA1 EER1 RCN1 REGA At1g25490 F2J7.19	Arabidopsis thaliana (Mouse-ear cress)	588
Q96303	PHT14_ARATH	Inorganic phosphate transporter 1-4 (AtPht1;4) (H	PHT1-4 PHT4 PT2 At2g38940 T7F6.11	Arabidopsis thaliana (Mouse-ear cress)	534
Q9SJL8	METK3_ARATH	S-adenosylmethionine synthase 3 (AdoMet syntha	METK3 At2g36880 T1J8.6	Arabidopsis thaliana (Mouse-ear cress)	390
Q8LC99	Q8LC99_ARATH	Adenine nucleotide alpha hydrolases-like superfar	At3g53990 AT3G53990	Arabidopsis thaliana (Mouse-ear cress)	126
Q8W4E6	HPAT1_ARATH	Hydroxyproline O-arabinosyltransferase 1 (EC 2.4	HPAT1 At5g25265	Arabidopsis thaliana (Mouse-ear cress)	366
Q56WD9	THIK2_ARATH	3-ketoacyl-CoA thiolase 2, peroxisomal (EC 2.3.1.	PED1 KAT2 At2g33150 F25I18.11	Arabidopsis thaliana (Mouse-ear cress)	462
F4IVR2	F4IVR2_ARATH	Heat shock protein 60-2	HSP60-2 heat shock protein 60-2 At2g33210 AT2G33210	Arabidopsis thaliana (Mouse-ear cress)	580
F4HYF3	DCYD1_ARATH	Bifunctional D-cysteine desulfhydrase/1-aminocyc	DCD ACD1 At1g48420 F11A17.2 T1N15.3	Arabidopsis thaliana (Mouse-ear cress)	401
Q84MD8	FHYRK_ARATH	Bifunctional riboflavin kinase/FMN phosphatase [I	FHY FMN At4g21470 F18E5.90	Arabidopsis thaliana (Mouse-ear cress)	379
A8MR27	A8MR27_ARATH	Polypyrimidine tract-binding protein 3	PTB3 At1g43190 AT1G43190 F1I21.14 F1I21_14	Arabidopsis thaliana (Mouse-ear cress)	430
P51414	RL261_ARATH	60S ribosomal protein L26-1	RPL26A At3g49910 F3A4.4 T16K5.260	Arabidopsis thaliana (Mouse-ear cress)	146

B3H749	B3H749_ARATH	Inositol-1-monophosphatase (EC 3.1.3.25)	VTC4 At3g02870 AT3G02870 F13E7.19 F13E7_19	Arabidopsis thaliana (Mouse-ear cress)	268
Q9FLV0	DMR6_ARATH	Protein DOWNY MILDEW RESISTANCE 6 (AtDM	DMR6 At5g24530 K18P6.6	Arabidopsis thaliana (Mouse-ear cress)	341
Q8VZ19	RL302_ARATH	60S ribosomal protein L30-2	RPL30B At1g77940 F28K19.15	Arabidopsis thaliana (Mouse-ear cress)	112
O49299	PGMC1_ARATH	Probable phosphoglucomutase, cytoplasmic 1 (P(	At1g23190 F26F24.1 T26J12.5	Arabidopsis thaliana (Mouse-ear cress)	583
Q94A40	COPA1_ARATH	Coatomer subunit alpha-1 (Alpha-coat protein 1) (	At1g62020 F8K4.21	Arabidopsis thaliana (Mouse-ear cress)	1216
F4IW47	TKTC2_ARATH	Transketolase-2, chloroplastic (TK) (EC 2.2.1.1)	TKL-2 At2g45290 F4L23.20	Arabidopsis thaliana (Mouse-ear cress)	741
A8MRV1	A8MRV1_ARATH	Histone H4	At1g07660 AT1G07660 F24B9.25	Arabidopsis thaliana (Mouse-ear cress)	86
P49209	RL91_ARATH	60S ribosomal protein L9-1	RPL9B At1g33120 T9L6.2; RPL9C At1g33140 T9L6.5	Arabidopsis thaliana (Mouse-ear cress)	194
F4HUJ2	RL101_ARATH	60S ribosomal protein L10-1 (Ribosomal protein R	RPL10A SAC52 At1g14320 F14L17.9	Arabidopsis thaliana (Mouse-ear cress)	220
Q9SRZ4	PRX2C_ARATH	Peroxiredoxin-2C (EC 1.11.1.25) (Glutaredoxin-de	PRXIIC TPX2 At1g65970 F12P19.13	Arabidopsis thaliana (Mouse-ear cress)	162
F4J5S0	F4J5S0_ARATH	Clustered mitochondria protein homolog	NOXY38 FMT FRIENDLY FRIENDLY MITOCHONDRIA n	Arabidopsis thaliana (Mouse-ear cress)	1396
Q38900	CP19A_ARATH	Peptidyl-prolyl cis-trans isomerase CYP19-1 (PPIa	CYP19-1 ROC3 At2g16600 T24I21.1	Arabidopsis thaliana (Mouse-ear cress)	173
Q38814	THI4_ARATH	Thiamine thiazole synthase, chloroplastic (EC 2.4.	THI1 ARA6 THI4 At5g54770 MBG8.3	Arabidopsis thaliana (Mouse-ear cress)	349
F4IL52	F4IL52_ARATH	Thioredoxin family protein	UNE5 ATPDI11 ATPDIL2-1 MEE30 PDI-LIKE 2-1 PDI11 F	Arabidopsis thaliana (Mouse-ear cress)	323
Q9C9W5	HPR1_ARATH	Glycerate dehydrogenase HPR, peroxisomal (GDH	HPR At1g68010 T23K23.14	Arabidopsis thaliana (Mouse-ear cress)	386
F4I3L1	F4I3L1_ARATH	Phosphoglycerate kinase (EC 2.7.2.3)	At1g56190 AT1G56190 F14G9.19 F14G9_19	Arabidopsis thaliana (Mouse-ear cress)	405
Q8VYM2	PHT11_ARATH	Inorganic phosphate transporter 1-1 (AtPht1;1) (H	PHT1-1 APT2 PHT1 PT1 At5g43350 MWF20.4	Arabidopsis thaliana (Mouse-ear cress)	524
P16181	RS111_ARATH	40S ribosomal protein S11-1 (Protein EMBRYO DI	RPS11A EMB1080 RPS11 At3g48930 T2J13.230	Arabidopsis thaliana (Mouse-ear cress)	160
O23247	SYRM_ARATH	Arginine--tRNA ligase, chloroplastic/mitochondrial	EMB1027 At4g26300 T25K17.110	Arabidopsis thaliana (Mouse-ear cress)	642
F4J1V2	F4J1V2_ARATH	DNAJ homologue 3	J3 ATJ ATJ3 DNAJ homologue 3 At3g44110 AT3G44110	Arabidopsis thaliana (Mouse-ear cress)	343
F4HZQ7	WTR5_ARATH	WAT1-related protein At1g21890	At1g21890 T26F17.11	Arabidopsis thaliana (Mouse-ear cress)	389
Q9ZUX4	UMP2_ARATH	Uncharacterized protein At2g27730, mitochondria	At2g27730 F15K20.17	Arabidopsis thaliana (Mouse-ear cress)	113
F4J7H2	TCPH_ARATH	T-complex protein 1 subunit eta (TCP-1-eta) (CCT	CCT7 At3g11830 F26K24.12	Arabidopsis thaliana (Mouse-ear cress)	557
Q43727	G6PD1_ARATH	Glucose-6-phosphate 1-dehydrogenase 1, chlorop	G6PD1 APG1 At5g35790 MIK22.2 MWP19.3	Arabidopsis thaliana (Mouse-ear cress)	576
Q9SJQ0	Q9SJQ0_ARATH	Pyruvate kinase (EC 2.7.1.40)	At2g36580 AT2G36580 F1O11.21 F1O11_21	Arabidopsis thaliana (Mouse-ear cress)	527
Q9LET1	CAMK7_ARATH	CDPK-related kinase 7 (AtCRK7) (EC 2.7.11.1) (C	CRK7 At3g56760 T8M16.90	Arabidopsis thaliana (Mouse-ear cress)	577
O80674	BH106_ARATH	Transcription factor bHLH106 (Basic helix-loop-he	BHLH106 EN56 At2g41130 T3K9.10	Arabidopsis thaliana (Mouse-ear cress)	253
Q946J2	SUVR1_ARATH	Probable inactive histone-lysine N-methyltransfera	SUVR1 SDG13 SET13 At1g04050 F20D22.16 F21M11.1	Arabidopsis thaliana (Mouse-ear cress)	734
Q5XVJ4	FAN1_ARATH	Fanconi-associated nuclease 1 homolog (AtFAN1	FAN1 At1g48360 F11A17.9	Arabidopsis thaliana (Mouse-ear cress)	891
Q9STF4	Q9STF4_ARATH	Cysteine/Histidine-rich C1 domain family protein (L	T6H20.170 At3g46800 AT3G46800	Arabidopsis thaliana (Mouse-ear cress)	682
F4IBH5	F4IBH5_ARATH	Mitochondrial proton/calcium exchanger protein (L	LETM2 AtLETM2 At1g65540 AT1G65540 F5I14.7 F5I14_	Arabidopsis thaliana (Mouse-ear cress)	736
F4ISG1	F4ISG1_ARATH	RING/FYVE/PHD zinc finger superfamily protein	At2g19260 AT2G19260 F27F23.6 F27F23_6	Arabidopsis thaliana (Mouse-ear cress)	631
F4IXI9	F4IXI9_ARATH	Dentin sialophosphoprotein-like protein	MXL8.16 AT3G21290	Arabidopsis thaliana (Mouse-ear cress)	1192
Q8GUG1	Q8GUG1_ARATH	At1g20030 (Calreticulin, putative) (Pathogenesis-r	At1g20030 AT1G20030 T20H2.19 T20H2_19	Arabidopsis thaliana (Mouse-ear cress)	299
Q9SYD8	DABB1_ARATH	Stress-response A/B barrel domain-containing pro	DABB1 At1g51360 F11M15.22	Arabidopsis thaliana (Mouse-ear cress)	210
Q84R08	Q84R08_ARATH	Putative CHP-rich zinc finger protein (Uncharacter	AT3G21210	Arabidopsis thaliana (Mouse-ear cress)	804
Q8LEV3	Y2060_ARATH	BTB/POZ domain-containing protein At2g30600	At2g30600/At2g30610 T6B20.5/T6B20.4	Arabidopsis thaliana (Mouse-ear cress)	809
F4HXV4	F4HXV4_ARATH	Microtubule-associated proteins 70-4	MAP70-4 ATMAP70-4 At1g14840 AT1G14840 F10B6.24	Arabidopsis thaliana (Mouse-ear cress)	589
O22812	AHL10_ARATH	AT-hook motif nuclear-localized protein 10 (AT-ho	AHL10 AHP1 At2g33620 F4P9.39	Arabidopsis thaliana (Mouse-ear cress)	351
Q94FN2	MOR1_ARATH	Protein MOR1 (Protein GEM1) (Protein GEMINI P(	MOR1 GEM1 RID5 At2g35630 T20F21.17	Arabidopsis thaliana (Mouse-ear cress)	1978
Q9FLW9	PKP2_ARATH	Plastidial pyruvate kinase 2 (PKp2) (EC 2.7.1.40)	PKP2 PKP1 At5g52920 MXC20.15	Arabidopsis thaliana (Mouse-ear cress)	579
Q9LVD3	PP434_ARATH	Pentatricopeptide repeat-containing protein At5g5	At5g57250 MJB24.6	Arabidopsis thaliana (Mouse-ear cress)	971
Q9SKT7	NDB4_ARATH	External alternative NAD(P)H-ubiquinone oxidored	NDB4 At2g20800 F5H14	Arabidopsis thaliana (Mouse-ear cress)	582
Q9M1H1	Q9M1H1_ARATH	Calmodulin-binding protein-related (Plant calmodu	T14E10_140 At3g54570 AT3G54570	Arabidopsis thaliana (Mouse-ear cress)	417
Q0WSH6	PP312_ARATH	Pentatricopeptide repeat-containing protein At4g1	LOI1 PCMP-H4 At4g14850 dl3465w FCAALL.335	Arabidopsis thaliana (Mouse-ear cress)	684
Q67XP8	Q67XP8_ARATH	Calcium-dependent lipid-binding (CaLB domain) fa	NTMC2T6.2 ntmc2T6.2 NTMC2TYPE6.2 At3g14590 AT3	Arabidopsis thaliana (Mouse-ear cress)	692
Q9SHK9	Q9SHK9_ARATH	Cysteine/Histidine-rich C1 domain family protein (U	MJB20.16 MJB20_16 At2g17600 AT2G17600	Arabidopsis thaliana (Mouse-ear cress)	580
Q42479	CDPK3_ARATH	Calcium-dependent protein kinase 3 (EC 2.7.11.1)	CPK3 CDPK6 At4g23650 F9D16.120	Arabidopsis thaliana (Mouse-ear cress)	529
Q9LNZ0	Q9LNZ0_ARATH	Cysteine/Histidine-rich C1 domain family protein (F	At1g44050 AT1G44050 F9C16.28 F9C16_28	Arabidopsis thaliana (Mouse-ear cress)	734
A8MS41	CCR4D_ARATH	Carbon catabolite repressor protein 4 homolog 4 (	CCR4-4 HESP At1g31500 F27M3_27 T8E3.7	Arabidopsis thaliana (Mouse-ear cress)	417
B7ZWR6	OEP61_ARATH	Outer envelope protein 61 (Tetratricopeptide repe	OEP61 TPR7 At5g21990 T6G21.100	Arabidopsis thaliana (Mouse-ear cress)	554
F4I0L1	F4I0L1_ARATH	Nuclear transport factor 2 (NTF2) family protein wi	At1g69250 AT1G69250 F4N2.20	Arabidopsis thaliana (Mouse-ear cress)	389
Q38821	2ABA_ARATH	Serine/threonine protein phosphatase 2A 55 kDa r	PP2AB1 At1g51690 F19C24.10	Arabidopsis thaliana (Mouse-ear cress)	513
Q9C5Z3	EIF3E_ARATH	Eukaryotic translation initiation factor 3 subunit E	TIF3E1 INT6 At3g57290 F28O9.140	Arabidopsis thaliana (Mouse-ear cress)	441
Q9FM82	GGLO4_ARATH	Probable L-gulonolactone oxidase 4 (AtGulLO4) (I	GULLO4 At5g56490 MCD7.26	Arabidopsis thaliana (Mouse-ear cress)	577
Q9FVC8	DAPA2_ARATH	4-hydroxy-tetrahydrodipicolinate synthase 2, chlor	DHDPS2 At2g45440 F4L23.5	Arabidopsis thaliana (Mouse-ear cress)	365
Q9LFE4	Y5673_ARATH	WEB family protein At5g16730, chloroplastic	At5g16730 F5E19_70	Arabidopsis thaliana (Mouse-ear cress)	853
Q9LFY0	PTST2_ARATH	Protein PTST homolog 2, chloroplastic (PROTEIN	PTST2 At1g27070 T7N9.13	Arabidopsis thaliana (Mouse-ear cress)	532
Q9LYA5	Q9LYA5_ARATH	Thioredoxin superfamily protein (Uncharacterized	AT5G14240 F18O22.30 F18O22_30	Arabidopsis thaliana (Mouse-ear cress)	256
P93819	MDHC1_ARATH	Malate dehydrogenase 1, cytoplasmic (EC 1.1.1.3	MDH1 At1g04410 F19P19.13	Arabidopsis thaliana (Mouse-ear cress)	332
Q9SMX3	VDAC3_ARATH	Mitochondrial outer membrane protein porin 3 (Pro	VDAC3 HSR2 At5g15090 F2G14.210	Arabidopsis thaliana (Mouse-ear cress)	274
Q9SJQ9	ALFC6_ARATH	Fructose-bisphosphate aldolase 6, cytosolic (AtFB	FBA6 At2g36460	Arabidopsis thaliana (Mouse-ear cress)	358
P48641	GSHRC_ARATH	Glutathione reductase, cytosolic (GR) (GRase) (E(	At3g24170 MUJ8.7	Arabidopsis thaliana (Mouse-ear cress)	499
Q8VZ74	ERA_ARATH	GTPase ERA-like, chloroplastic (GTP-binding prot	At5g66470 K1F13.13	Arabidopsis thaliana (Mouse-ear cress)	427
Q56YW9	TBB2_ARATH	Tubulin beta-2 chain	TUBB2 TUB2 At5g62690 MRG21.11	Arabidopsis thaliana (Mouse-ear cress)	450
F4I3P5	F4I3P5_ARATH	GMP synthase (glutamine-hydrolyzing) (EC 6.3.5.	At1g63660 AT1G63660 F24D7.15 F24D7_15	Arabidopsis thaliana (Mouse-ear cress)	434
Q8LAS8	SFGH_ARATH	S-formylglutathione hydrolase (AtSFGH) (EC 3.1.2	SFGH At2g41530 T32G6.5	Arabidopsis thaliana (Mouse-ear cress)	284
Q9C5M0	DTC_ARATH	Mitochondrial dicarboxylate/tricarboxylate transpor	DTC At5g19760 T29J13.180	Arabidopsis thaliana (Mouse-ear cress)	298
Q9FIL6	Q9FIL6_ARATH	At5g58950 (Protein kinase superfamily protein) (P	At5g58950 AT5G58950 K19M22.20 K19M22_20	Arabidopsis thaliana (Mouse-ear cress)	525
Q9SRH5	VDAC1_ARATH	Mitochondrial outer membrane protein porin 1 (Vo	VDAC1 At3g01280 T22N4.9	Arabidopsis thaliana (Mouse-ear cress)	276
F4J3P1	F4J3P1_ARATH	Ribosomal protein L14p/L23e family protein	emb2171 embryo defective 2171 At2g33370 At3g04400 /	Arabidopsis thaliana (Mouse-ear cress)	125
Q9LP45	PSD11_ARATH	26S proteasome non-ATPase regulatory subunit 1	RPN6 ATS9 RPN6A At1g29150 F28N24.15	Arabidopsis thaliana (Mouse-ear cress)	419
Q9SKP6	TPIC_ARATH	Triosephosphate isomerase, chloroplastic (TIM) (T	TIM At2g21170 F26H11.7	Arabidopsis thaliana (Mouse-ear cress)	315
F4JHW1	F4JHW1_ARATH	Phenazine biosynthesis PhzC/PhzF protein	At4g02860 AT4G02860 T5J8.18 T5J8_18	Arabidopsis thaliana (Mouse-ear cress)	313
O23569	O23569_ARATH	L-lactate dehydrogenase (EC 1.1.1.27)	dl4665w DL4665W At4g17260 AT4G17260 FCAALL.172	Arabidopsis thaliana (Mouse-ear cress)	353
Q9SA06	Q9SA06_ARATH	F28K20.6 protein (Polyadenylate-binding protein 1	At1g31130 AT1G31130 F28K20.6 F28K20_6	Arabidopsis thaliana (Mouse-ear cress)	321
Q9FK88	INVE_ARATH	Alkaline/neutral invertase E, chloroplastic (A/N-IN\	INVE At5g22510 MQJ16.5	Arabidopsis thaliana (Mouse-ear cress)	617
Q8LCL3	RL272_ARATH	60S ribosomal protein L27-2	RPL27B At3g22230 MKA23.13	Arabidopsis thaliana (Mouse-ear cress)	135
Q42347	RL241_ARATH	60S ribosomal protein L24-1	RPL24A At2g36620 F13K3.2 F1O11.25	Arabidopsis thaliana (Mouse-ear cress)	164
P29516	TBB8_ARATH	Tubulin beta-8 chain (Beta-8-tubulin)	TUBB8 TUB8 At5g23860 MRO11.10	Arabidopsis thaliana (Mouse-ear cress)	449
Q9LFA3	MDAR1_ARATH	Monodehydroascorbate reductase 1, peroxisomal	MDAR1 At3g52880 F8J2.50	Arabidopsis thaliana (Mouse-ear cress)	434
Q9SF47	PAH1_ARATH	Phosphatidate phosphatase PAH1 (EC 3.1.3.4) (F	PAH1 At3g09560 F11F8.14	Arabidopsis thaliana (Mouse-ear cress)	904
Q9ZRE2	RABD1_ARATH	Ras-related protein RABD1 (AtRABD1) (Ras-relat	RABD1 ATFP8 At3g11730 F26K24.2	Arabidopsis thaliana (Mouse-ear cress)	205
Q42521	DCE1_ARATH	Glutamate decarboxylase 1 (GAD 1) (EC 4.1.1.15	GAD1 GAD GDH1 At5g17330 MKP11.30 MKP11_18	Arabidopsis thaliana (Mouse-ear cress)	502
Q9C869	MCC02_ARATH	MATH domain and coiled-coil domain-containing p	At1g31400 T19E23.18 T8E3.21	Arabidopsis thaliana (Mouse-ear cress)	278
O23034	UBA5_ARATH	Ubiquitin-like modifier-activating enzyme 5 (Ubiqu	At1g05350 YUP8H12.3	Arabidopsis thaliana (Mouse-ear cress)	431
O82204	RL281_ARATH	60S ribosomal protein L28-1 (Protein ASYMMETR	RPL28A AE5 At2g19730 F6F22.24	Arabidopsis thaliana (Mouse-ear cress)	143
Q9SR73	RS281_ARATH	40S ribosomal protein S28-1	RPS28A At3g10090 T22K18.8; RPS28B At5g03850 F8F(	Arabidopsis thaliana (Mouse-ear cress)	64
Q56WN1	GLN11_ARATH	Glutamine synthetase cytosolic isozyme 1-1 (EC 6	GLN1-1 At5g37600 K12B20.50	Arabidopsis thaliana (Mouse-ear cress)	356
Q9C4Z6	GPLPB_ARATH	Receptor for activated C kinase 1B (Guanine nucl	RACK1B At1g48630 F11I4.18 F9P7.2	Arabidopsis thaliana (Mouse-ear cress)	326
P25697	KPPR_ARATH	Phosphoribulokinase, chloroplastic (PRK) (PRKas	At1g32060 T12O21.4	Arabidopsis thaliana (Mouse-ear cress)	395
Q8GUM2	HSP7I_ARATH	Heat shock 70 kDa protein 9, mitochondrial (Chap	HSP70-9 HSCA1 MTHSC70-1 At4g37910 F20D10.30	Arabidopsis thaliana (Mouse-ear cress)	682
Q0WWQ1	ATG3_ARATH	Autophagy-related protein 3 (Autophagy-related E	ATG3 APG3 At5g61500 K11J9.3	Arabidopsis thaliana (Mouse-ear cress)	313
P06525	ADH1_ARATH	Alcohol dehydrogenase class-P (AtADH) (EC 1.1.	ADH1 ADH At1g77120 F22K20.19	Arabidopsis thaliana (Mouse-ear cress)	379
Q43725	CYSKM_ARATH	Cysteine synthase, mitochondrial (EC 2.5.1.47) (B	OASC ACS 1 At3g59760 F24G16.30	Arabidopsis thaliana (Mouse-ear cress)	430
Q9FJY6	Q9FJY6_ARATH	glucose-6-phosphate 1-epimerase (EC 5.1.3.15)	At5g66530 AT5G66530 K1F13.19 K1F13_19	Arabidopsis thaliana (Mouse-ear cress)	307
Q9ZT91	EFTM_ARATH	Elongation factor Tu, mitochondrial	TUFA At4g02930 T4I9.19 T5J8.25	Arabidopsis thaliana (Mouse-ear cress)	454
Q96255	SERB1_ARATH	Phosphoserine aminotransferase 1, chloroplastic (	PSAT1 At4g35630 F8D20.140	Arabidopsis thaliana (Mouse-ear cress)	430
P57106	MDHC2_ARATH	Malate dehydrogenase 2, cytoplasmic (EC 1.1.1.3	MDH2 At5g43330 MWF20.2	Arabidopsis thaliana (Mouse-ear cress)	332
F4J912	F4J912_ARATH	Ribosomal protein L5	ATL5 OLI5 OLIGOCELLULA 5 PGY3 PIGGYBACK3 RIB(	Arabidopsis thaliana (Mouse-ear cress)	190
F4K5C7	F4K5C7_ARATH	40S ribosomal protein S4	At5g07090 AT5G07090 T28J14.30	Arabidopsis thaliana (Mouse-ear cress)	244
Q9XF89	CB5_ARATH	Chlorophyll a-b binding protein CP26, chloroplasti	LHCB5 At4g10340 F24G24.140	Arabidopsis thaliana (Mouse-ear cress)	280
O04499	PMG1_ARATH	2,3-bisphosphoglycerate-independent phosphogly	PGM1 At1g09780 F21M12.16	Arabidopsis thaliana (Mouse-ear cress)	557
Q9FR44	PEAM1_ARATH	Phosphoethanolamine N-methyltransferase 1 (AtN	NMT1 PEAMT XPL1 At3g18000 MEB5.22	Arabidopsis thaliana (Mouse-ear cress)	491
P51427	RS52_ARATH	40S ribosomal protein S5-2 (AtRPS5A) (Protein A	RPS5B AML1 At3g11940 F26K24.23 MEC18.4	Arabidopsis thaliana (Mouse-ear cress)	207
P34788	RS18_ARATH	40S ribosomal protein S18	RPS18A PFL At1g22780 T22J18.5; RPS18B At1g34030	Arabidopsis thaliana (Mouse-ear cress)	152
P31167	ADT1_ARATH	ADP,ATP carrier protein 1, mitochondrial (ADP/AT	AAC1 ANT1 At3g08580 F17O14.5	Arabidopsis thaliana (Mouse-ear cress)	381
O48917	SQD1_ARATH	UDP-sulfoquinovose synthase, chloroplastic (EC 3	SQD1 At4g33030 F26P21.150	Arabidopsis thaliana (Mouse-ear cress)	477
P41916	RAN1_ARATH	GTP-binding nuclear protein Ran-1 (Ras-related n	RAN1 At5g20010 F28I16.160	Arabidopsis thaliana (Mouse-ear cress)	221
Q9FEF8	FBRL1_ARATH	rRNA 2'-O-methyltransferase fibrillarin 1 (AtFbr1) (	FIB1 FBR1 MED36_2 MED36B SKIP7 At5g52470 K24M7	Arabidopsis thaliana (Mouse-ear cress)	308
O80840	PMM_ARATH	Phosphomannomutase (AtPMM) (EC 5.4.2.8)	PMM At2g45790 F4I18.23	Arabidopsis thaliana (Mouse-ear cress)	246

Q9FJF1	Q9FJF1_ARATH	Putative GTP-binding protein ara-3 (Rab-type sma	MMN10.12 MMN10_12 At5g59840 AT5G59840	Arabidopsis thaliana (Mouse-ear cress)	216
Q9SRH6	HIR3_ARATH	Hypersensitive-induced response protein 3 (AtHIR	HIR3 P31 At3g01290 T22N4.8 T4P13.3	Arabidopsis thaliana (Mouse-ear cress)	285
Q93VG5	RS81_ARATH	40S ribosomal protein S8-1	RPS8A At5g20290 F5O24_180	Arabidopsis thaliana (Mouse-ear cress)	222
Q9LF46	HACL_ARATH	2-hydroxyacyl-CoA lyase (EC 4.1.-.-) (2-hydroxyph	HACL HPCL OCD At5g17380 T10B6.40	Arabidopsis thaliana (Mouse-ear cress)	572
Q9LK96	Q9LK96_ARATH	AT3g15090/K15M2_24 (GroES-like zinc-binding a	AT3G15090	Arabidopsis thaliana (Mouse-ear cress)	366
B9DFQ9	B9DFQ9_ARATH	AT5G37510 protein (NADH-ubiquinone dehydroge	EMB1467 CI76 embryo defective 1467 MPA22.5 MPA22_	Arabidopsis thaliana (Mouse-ear cress)	745
Q8W4M5	PFPB1_ARATH	Pyrophosphate--fructose 6-phosphate 1-phosphot	PFP-BETA1 At1g12000 F12F1.13	Arabidopsis thaliana (Mouse-ear cress)	566
Q9SRI1	SC13A_ARATH	Protein transport protein SEC13 homolog A (SEC	SEC13A At3g01340 T22N4.3	Arabidopsis thaliana (Mouse-ear cress)	302
Q94EG6	Y5224_ARATH	Uncharacterized protein At5g02240	At5g02240 T7H20_290	Arabidopsis thaliana (Mouse-ear cress)	253
P32962	NRL2_ARATH	Nitrilase 2 (EC 3.5.5.1)	NIT2 At3g44300 T10D17_90	Arabidopsis thaliana (Mouse-ear cress)	339
F4JYE1	F4JYE1_ARATH	Biotin carboxylase (EC 6.3.4.14) (Acetyl-coenzym	CAC2 ACETYL-COA CARBOXYLASE At5g35360 AT5G3	Arabidopsis thaliana (Mouse-ear cress)	499
P50883	RL121_ARATH	60S ribosomal protein L12-1	RPL12A At2g37190 T2N18.5	Arabidopsis thaliana (Mouse-ear cress)	166
Q39251	ADF2_ARATH	Actin-depolymerizing factor 2 (ADF-2) (AtADF2)	ADF2 At3g46000 F16L2_210	Arabidopsis thaliana (Mouse-ear cress)	137
Q9SE60	MTHR1_ARATH	Methylenetetrahydrofolate reductase (NADH) 1 (A	MTHFR1 At3g59970 F24G16.240	Arabidopsis thaliana (Mouse-ear cress)	592
O81149	PSA5A_ARATH	Proteasome subunit alpha type-5-A (20S proteaso	PAE1 At1g53850 T18A20.8	Arabidopsis thaliana (Mouse-ear cress)	237
F4JWM1	F4JWM1_ARATH	Ribosomal protein S5 domain 2-like superfamily p	At5g18380 AT5G18380 F20L16.100 F20L16_100	Arabidopsis thaliana (Mouse-ear cress)	139
O23715	PSA3_ARATH	Proteasome subunit alpha type-3 (20S proteasom	PAG1 PRC8 At2g27020 T20P8.7	Arabidopsis thaliana (Mouse-ear cress)	249
O23627	SYGM1_ARATH	Glycine--tRNA ligase, mitochondrial 1 (EC 6.1.1.1	At1g29880 F1N18.8	Arabidopsis thaliana (Mouse-ear cress)	729
Q9M9P3	UGPA2_ARATH	UTP--glucose-1-phosphate uridylyltransferase 2 (	UGP2 At3g03250 T17B22.6	Arabidopsis thaliana (Mouse-ear cress)	469
P12411	TBB1_ARATH	Tubulin beta-1 chain (Beta-1-tubulin)	TUBB1 TUB1 At1g75780 F10A5.3 T4O12.1	Arabidopsis thaliana (Mouse-ear cress)	447
Q93VR3	GME_ARATH	GDP-mannose 3,5-epimerase (GDP-Man 3,5-epin	At5g28840 F7P1.20	Arabidopsis thaliana (Mouse-ear cress)	377
Q944G9	ALFP2_ARATH	Fructose-bisphosphate aldolase 2, chloroplastic (	FBA2 At4g38970 F19H22.70	Arabidopsis thaliana (Mouse-ear cress)	398
Q9T043	RL142_ARATH	60S ribosomal protein L14-2	RPL14B At4g27090 T24A18.40	Arabidopsis thaliana (Mouse-ear cress)	134
Q8GYE0	PHF1_ARATH	SEC12-like protein 1 (Protein PHOSPHATE TRAN	PHF1 At3g52190 F4F15.300	Arabidopsis thaliana (Mouse-ear cress)	398
Q0WW26	COPG_ARATH	Coatomer subunit gamma (Gamma-coat protein) (	At4g34450 T4L20.30	Arabidopsis thaliana (Mouse-ear cress)	886
P60039	RL73_ARATH	60S ribosomal protein L7-3	RPL7C At2g44120 F6E13.25	Arabidopsis thaliana (Mouse-ear cress)	242
P92549	ATPAM_ARATH	ATP synthase subunit alpha, mitochondrial	ATPA ATP1 AtMg01190	Arabidopsis thaliana (Mouse-ear cress)	507
F4JBC7	F4JBC7_ARATH	HXXXD-type acyl-transferase family protein	At3g26040 AT3G26040	Arabidopsis thaliana (Mouse-ear cress)	442
Q1JPL7	PME18_ARATH	Pectinesterase/pectinesterase inhibitor 18 (AtPME	PME18 ARATH4 At1g11580 T23J18.23 T23J18.24 T23J	Arabidopsis thaliana (Mouse-ear cress)	557
Q96266	GSTF8_ARATH	Glutathione S-transferase F8, chloroplastic (AtGS	GSTF8 GST6 GSTF5 At2g47730 F17A22.12	Arabidopsis thaliana (Mouse-ear cress)	263
O23255	SAHH1_ARATH	Adenosylhomocysteinase 1 (AdoHcyase 1) (EC 3.	SAHH1 EMB1395 HOG1 At4g13940 dl3010w	Arabidopsis thaliana (Mouse-ear cress)	485
P49211	RL321_ARATH	60S ribosomal protein L32-1	RPL32A At4g18100 F15J5.70	Arabidopsis thaliana (Mouse-ear cress)	133
P93031	GMD2_ARATH	GDP-mannose 4,6 dehydratase 2 (EC 4.2.1.47) (	MUR1 GMD2 At3g51160 F24M12.200	Arabidopsis thaliana (Mouse-ear cress)	373
Q5E924	G3PP2_ARATH	Glyceraldehyde-3-phosphate dehydrogenase GAF	GAPCP2 At1g16300 F3O9.10	Arabidopsis thaliana (Mouse-ear cress)	420
P25851	F16P1_ARATH	Fructose-1,6-bisphosphatase 1, chloroplastic (FBF	CFBP1 FBP HCEF1 At3g54050 F24B22.10	Arabidopsis thaliana (Mouse-ear cress)	417
F4J378	F4J378_ARATH	Histone deacetylase 3	HDA3 ATHD2A HD2A HDT1 HISTONE DEACETYLASE 2	Arabidopsis thaliana (Mouse-ear cress)	242
Q9M084	SYDC2_ARATH	Aspartate--tRNA ligase 2, cytoplasmic (EC 6.1.1.1	IBI1 At4g31180	Arabidopsis thaliana (Mouse-ear cress)	558
Q93Y22	COPD_ARATH	Coatomer subunit delta (Delta-coat protein) (Delta	At5g05010 MUG13.13	Arabidopsis thaliana (Mouse-ear cress)	527
Q945K7	IDH5_ARATH	Isocitrate dehydrogenase [NAD] catalytic subunit 5	IDH5 At5g03290 F12E4_20 MOK16.20	Arabidopsis thaliana (Mouse-ear cress)	374
P10795	RBS1A_ARATH	Ribulose bisphosphate carboxylase small subunit	RBCS-1A ATS1A At1g67090 F1O19.14 F5A8.1	Arabidopsis thaliana (Mouse-ear cress)	180
Q38882	PLDA1_ARATH	Phospholipase D alpha 1 (AtPLDalpha1) (PLD alp	PLDALPHA1 PLD1 At3g15730 MSJ11.13	Arabidopsis thaliana (Mouse-ear cress)	810
F4IVQ2	F4IVQ2_ARATH	Synaptobrevin-related protein 1	SAR1 ATVAMP722 VAMP722 At2g33120 AT2G33120 F2	Arabidopsis thaliana (Mouse-ear cress)	139
Q9M5K3	DLDH1_ARATH	Dihydrolipoyl dehydrogenase 1, mitochondrial (Atr	LPD1 At1g48030 F21D18.28 T2J15.6	Arabidopsis thaliana (Mouse-ear cress)	507
Q9LV35	AIP12_ARATH	Actin-interacting protein 1-2	AIP1-2 At3g18060 MRC8.4	Arabidopsis thaliana (Mouse-ear cress)	609
Q9T0P4	GLTB2_ARATH	Ferredoxin-dependent glutamate synthase 2, chlo	GLU2 At2g41220 F13H10.23	Arabidopsis thaliana (Mouse-ear cress)	1629
Q56WH1	TBA3_ARATH	Tubulin alpha-3 chain (EC 3.6.5.-)	TUBA3 TUA3 At5g19770 T29J13.190	Arabidopsis thaliana (Mouse-ear cress)	450
Q9FVQ1	NUCL1_ARATH	Nucleolin 1 (Protein NUCLEOLIN LIKE 1) (AtNUC	NUCL1 NUC1 PARL1 At1g48920 F27K7.6	Arabidopsis thaliana (Mouse-ear cress)	557
Q9M1R2	SYPC_ARATH	Proline--tRNA ligase, cytoplasmic (EC 6.1.1.15) (F	At3g62120 T17J13.80	Arabidopsis thaliana (Mouse-ear cress)	530
Q8LE63	Q8LE63_ARATH	FAM63A-like protein (DUF544) (Uncharacterized p	AT4G11860 T26M18.70 T26M18_70	Arabidopsis thaliana (Mouse-ear cress)	682
Q9FHX6	Q9FHX6_ARATH	Dbj|BAA84809.1 (Lung seven transmembrane rec	MJC20.20 MJC20_20 At5g42090 At5g42090/MJC20.20 A	Arabidopsis thaliana (Mouse-ear cress)	439
P0DH99	EF1A1_ARATH	Elongation factor 1-alpha 1 (EF-1-alpha 1) (eEF-1	A1 At1g07940 T6D22.3	Arabidopsis thaliana (Mouse-ear cress)	449
Q9C9C4	ENO1_ARATH	Enolase 1, chloroplastic (EC 4.2.1.11) (2-phospho	ENO1 At1g74030 F2P9.10	Arabidopsis thaliana (Mouse-ear cress)	477
P30186	PSA7A_ARATH	Proteasome subunit alpha type-7-A (20S proteaso	PAD1 PRC6A At3g51260 F24M12.300	Arabidopsis thaliana (Mouse-ear cress)	250
P46645	AAT2_ARATH	Aspartate aminotransferase, cytoplasmic isozyme	ASP2 AAT2 At5g19550 T20D1.70	Arabidopsis thaliana (Mouse-ear cress)	405
O64777	Y1643_ARATH	G-type lectin S-receptor-like serine/threonine-prot	At1g61430 T1F9.8	Arabidopsis thaliana (Mouse-ear cress)	806
P20115	CISY4_ARATH	Citrate synthase 4, mitochondrial (EC 2.3.3.16)	CSY4 At2g44350 F4I1.16	Arabidopsis thaliana (Mouse-ear cress)	474
P22953	HS701_ARATH	Heat shock 70 kDa protein 1 (Heat shock cognate	HSP70-1 ERD2 HSC70-1 MED37_4 MED37E At5g02500	Arabidopsis thaliana (Mouse-ear cress)	651
O49006	PME3_ARATH	Pectinesterase/pectinesterase inhibitor 3 [Includes	PME3 ARATH27 At3g14310 MLN21.10	Arabidopsis thaliana (Mouse-ear cress)	592
Q8RXU5	R37A2_ARATH	60S ribosomal protein L37a-2	RPL37AC At3g60245 F27H5.3	Arabidopsis thaliana (Mouse-ear cress)	92
O49485	SERA1_ARATH	D-3-phosphoglycerate dehydrogenase 1, chloropla	PGDH1 EDA9 At4g34200 F10M10.7	Arabidopsis thaliana (Mouse-ear cress)	603
O82663	SDHA1_ARATH	Succinate dehydrogenase [ubiquinone] flavoprote	SDH1-1 At5g66760 MSN2.16	Arabidopsis thaliana (Mouse-ear cress)	634
Q9SIV2	PSD2A_ARATH	26S proteasome non-ATPase regulatory subunit 2	RPN1A At2g20580 F23N11.10	Arabidopsis thaliana (Mouse-ear cress)	891
P48491	TPIS_ARATH	Triosephosphate isomerase, cytosolic (TIM) (Trios	CTIMC At3g55440 T22E16.100	Arabidopsis thaliana (Mouse-ear cress)	254
A1L4Y4	A1L4Y4_ARATH	At5g61510 (GroES-like zinc-binding alcohol dehyc	AT5G61510 K11J9.5 K11J9_5	Arabidopsis thaliana (Mouse-ear cress)	406
A8MQE5	A8MQE5_ARATH	Insulinase (Peptidase family M16) protein	At1g51980 AT1G51980 F5F19.4 F5F19_4	Arabidopsis thaliana (Mouse-ear cress)	451
F4HUL6	F4HUL6_ARATH	Catalase 3	CAT3 ATCAT3 catalase 3 SEN2 SENESCENCE 2 At1g2	Arabidopsis thaliana (Mouse-ear cress)	427
P93028	UBE11_ARATH	Ubiquitin-activating enzyme E1 1 (AtUBA1) (EC 6.	UBA1 MOS5 At2g30110 T27E13.15	Arabidopsis thaliana (Mouse-ear cress)	1080
P51430	RS62_ARATH	40S ribosomal protein S6-2 (Protein EMBRYO DE	RPS6B EMB3010 RPS6 At5g10360 F12B17_290	Arabidopsis thaliana (Mouse-ear cress)	249
Q9M1H3	AB4F_ARATH	ABC transporter F family member 4 (ABC transpo	ABCF4 GCN4 At3g54540 T14E10.110	Arabidopsis thaliana (Mouse-ear cress)	723
F4HZG6	IMPA4_ARATH	Importin subunit alpha-4 (IMPa-4)	IMPA4 At1g09270 T12M4.2	Arabidopsis thaliana (Mouse-ear cress)	538
Q9STY6	RS202_ARATH	40S ribosomal protein S20-2	RPS20B At3g47370 T21L8.120	Arabidopsis thaliana (Mouse-ear cress)	122
Q94BT0	SPSA1_ARATH	Sucrose-phosphate synthase 1 (EC 2.4.1.14) (Suc	SPS1 SPSA1 At5g20280 F5O24.170	Arabidopsis thaliana (Mouse-ear cress)	1043
Q9C550	LEU12_ARATH	2-isopropylmalate synthase 2, chloroplastic (EC 2.	IPMS2 IMS1 MAML-3 At1g74040 F2P9.9	Arabidopsis thaliana (Mouse-ear cress)	631
B9DG17	B9DG17_ARATH	40S ribosomal protein SA	P40 40s ribosomal protein SA AP40 RP40 RPSAA At1g7	Arabidopsis thaliana (Mouse-ear cress)	294
Q9LFW1	RGP2_ARATH	UDP-arabinopyranose mutase 2 (EC 5.4.99.30) (F	RGP2 At5g15650 F14F8_30	Arabidopsis thaliana (Mouse-ear cress)	360
Q9S757	CYSC1_ARATH	Bifunctional L-3-cyanoalanine synthase/cysteine s	CYSC1 OAS5 At3g61440 F2A19.40	Arabidopsis thaliana (Mouse-ear cress)	368
Q38799	ODPB1_ARATH	Pyruvate dehydrogenase E1 component subunit b	PDH2 MAB1 At5g50850 K16E14.1	Arabidopsis thaliana (Mouse-ear cress)	363
P49040	SUS1_ARATH	Sucrose synthase 1 (AtSUS1) (EC 2.4.1.13) (Sucr	SUS1 At5g20830 T1M15.230	Arabidopsis thaliana (Mouse-ear cress)	808
Q9FJH0	RAA1F_ARATH	Ras-related protein RABA1f (AtRABA1f)	RABA1F At5g60860 MAE1.9	Arabidopsis thaliana (Mouse-ear cress)	217
Q1WIQ6	GAPN_ARATH	NADP-dependent glyceraldehyde-3-phosphate de	ALDH11A3 GAPN At2g24270 F27D4.18	Arabidopsis thaliana (Mouse-ear cress)	496
O80501	RAH1B_ARATH	Ras-related protein RABH1b (AtRABH1b) (Ras-rel	RABH1B RAB6A At2g44610 F16B22.10	Arabidopsis thaliana (Mouse-ear cress)	208
Q8RWN9	ODP22_ARATH	Dihydrolipoyllysine-residue acetyltransferase com	At3g13930 MDC16.5	Arabidopsis thaliana (Mouse-ear cress)	539
Q93YU5	SEC8_ARATH	Exocyst complex component SEC8 (AtSec8) (Exo	SEC8 At3g10380 F14P13.2	Arabidopsis thaliana (Mouse-ear cress)	1053
Q9XEE2	ANXD2_ARATH	Annexin D2 (AnnAt2)	ANN2 ANNAT2 At5g65020 MXK3.27	Arabidopsis thaliana (Mouse-ear cress)	317
Q9FZ76	RL61_ARATH	60S ribosomal protein L6-1	RPL6A At1g18540 F25I16.12	Arabidopsis thaliana (Mouse-ear cress)	233
F4IP53	F4IP53_ARATH	General regulatory factor 9	GRF9 general regulatory factor 9 GF14 MU At2g42590 A	Arabidopsis thaliana (Mouse-ear cress)	262
P29197	CH60A_ARATH	Chaperonin CPN60, mitochondrial (HSP60)	CPN60 At3g23990 F14O13.18	Arabidopsis thaliana (Mouse-ear cress)	577
P42643	14331_ARATH	14-3-3-like protein GF14 chi (General regulatory f	GRF1 At4g09000 F23J3.30	Arabidopsis thaliana (Mouse-ear cress)	267
Q9SVY0	NRPA1_ARATH	DNA-directed RNA polymerase I subunit 1 (DNA-d	NRPA1 RPA1 At3g57660 F15B8.150	Arabidopsis thaliana (Mouse-ear cress)	1670
P17597	ILVB_ARATH	Acetolactate synthase, chloroplastic (AtALS) (EC	ALS AHAS CSR1 TZP5 At3g48560 T8P19.70	Arabidopsis thaliana (Mouse-ear cress)	670
Q42134	PSA5B_ARATH	Proteasome subunit alpha type-5-B (20S proteaso	PAE2 PRCZ At3g14290 MLN21.8	Arabidopsis thaliana (Mouse-ear cress)	237
Q9SRZ6	ICDHC_ARATH	Cytosolic isocitrate dehydrogenase [NADP] (EC 1.	CICDH At1g65930 F12P19.10	Arabidopsis thaliana (Mouse-ear cress)	410
Q9ZPI1	SYKC_ARATH	Lysine--tRNA ligase, cytoplasmic (EC 6.1.1.6) (Lys	At3g11710 T19F11.11	Arabidopsis thaliana (Mouse-ear cress)	626
Q9SA73	OLA1_ARATH	Obg-like ATPase 1 (Ribosome-binding ATPase Yc	YchF1 At1g30580 T5I8.3	Arabidopsis thaliana (Mouse-ear cress)	394
P42791	RL182_ARATH	60S ribosomal protein L18-2	RPL18B At3g05590 F18C1.14	Arabidopsis thaliana (Mouse-ear cress)	187
F4JA10	F4JA10_ARATH	Presequence protease 1	PREP1 ATPREP1 ATZNMP presequence protease 1 At3	Arabidopsis thaliana (Mouse-ear cress)	1069
F4KE21	F4KE21_ARATH	Biotin carboxyl carrier protein of acetyl-CoA carbo	CAC1 BCCP BCCP-1 BCCP1 CAC1-A CAC1A MQK4.12	Arabidopsis thaliana (Mouse-ear cress)	254
P19456	PMA2_ARATH	ATPase 2, plasma membrane-type (EC 7.1.2.1) (F	AHA2 At4g30190 F9N11.40	Arabidopsis thaliana (Mouse-ear cress)	948
P32961	NRL1_ARATH	Nitrilase 1 (EC 3.5.5.1)	NIT1 At3g44310 T10D17_100	Arabidopsis thaliana (Mouse-ear cress)	346
Q42449	PRF1_ARATH	Profilin-1 (AtPROF1) (AthPRF1) (allergen Ara t 8)	PRF1 PFN1 PRO1 At2g19760 F6F22.21	Arabidopsis thaliana (Mouse-ear cress)	131
P25857	G3PB_ARATH	Glyceraldehyde-3-phosphate dehydrogenase GAF	GAPB At1g42970 F13A11.3	Arabidopsis thaliana (Mouse-ear cress)	447
F4I577	F4I577_ARATH	Monodehydroascorbate reductase 6	MDAR6 At1g63940 AT1G63940	Arabidopsis thaliana (Mouse-ear cress)	416
P42644	14333_ARATH	14-3-3-like protein GF14 psi (General regulatory f	GRF3 RCI1A At5g38480 MXI10.21	Arabidopsis thaliana (Mouse-ear cress)	255
Q9SR33	ORP3B_ARATH	Oxysterol-binding protein-related protein 3B (OSB	ORP3B At3g09300 F3L24.17	Arabidopsis thaliana (Mouse-ear cress)	458
Q9C9C5	RL63_ARATH	60S ribosomal protein L6-3	RPL6C At1g74050 F2P9.8	Arabidopsis thaliana (Mouse-ear cress)	233
O65398	GLX1_ARATH	Lactoylglutathione lyase GLX1 (EC 4.4.1.5) (Glyo	GLX1 At1g11840 F12F1.32	Arabidopsis thaliana (Mouse-ear cress)	283
O23654	VATA_ARATH	V-type proton ATPase catalytic subunit A (V-ATPa	VHA-A At1g78900 F9K20.5	Arabidopsis thaliana (Mouse-ear cress)	623
Q0WNJ6	CLAH1_ARATH	Clathrin heavy chain 1	CHC1 At3g11130 F11B9.30 F9F8.6	Arabidopsis thaliana (Mouse-ear cress)	1705
P92963	RAB1C_ARATH	Ras-related protein RABB1c (AtRABB1c) (Ras-rel	RABB1C RAB2 RAB2A At4g17170 dl4620c FCAALL.365	Arabidopsis thaliana (Mouse-ear cress)	211
Q39141	Q39141_ARATH	Chlorophyll a-b binding protein, chloroplastic	Lhb1B2 LHB1B2 LHCB1.5 At2g34420 At2g34420/T31E1	Arabidopsis thaliana (Mouse-ear cress)	265
A8MS83	A8MS83_ARATH	Ribosomal protein L23AB	RPL23AB RIBOSOMAL PROTEIN L23A2 ribosomal prote	Arabidopsis thaliana (Mouse-ear cress)	148

F4JVN6	TPPII_ARATH	Tripeptidyl-peptidase 2 (EC 3.4.14.10) (Tripeptidyl	TPP2 At4g20850 T13K14.10	Arabidopsis thaliana (Mouse-ear cress)	1380
Q93ZN2	ALKR4_ARATH	Probable aldo-keto reductase 4 (EC 1.1.1.-)	At1g60710 F8A5.23	Arabidopsis thaliana (Mouse-ear cress)	345
Q01525	14332_ARATH	14-3-3-like protein GF14 omega (General regulato	GRF2 GF14 At1g78300 F3F9.16	Arabidopsis thaliana (Mouse-ear cress)	259
P30184	AMPL1_ARATH	Leucine aminopeptidase 1 (EC 3.4.11.1) (Leucyl a	LAP1 PM25 At2g24200 F27D4.11	Arabidopsis thaliana (Mouse-ear cress)	520
F4I529	F4I529_ARATH	Calreticulin	CRT1a AtCRT1a CALRETICULIN 1 calreticulin 1 calreticu	Arabidopsis thaliana (Mouse-ear cress)	424
O24653	GDI2_ARATH	Guanosine nucleotide diphosphate dissociation in	GDI2 At3g59920 F24G16.190	Arabidopsis thaliana (Mouse-ear cress)	444
Q9FIX1	AIGLB_ARATH	AIG2-like protein B (EC 2.3.2.-) (Avirulence-induce	AIG2LB At5g39730 MKM21.4	Arabidopsis thaliana (Mouse-ear cress)	172
B3H632	B3H632_ARATH	Succinate--CoA ligase [ADP-forming] subunit alph	MKD15.11 MKD15_11 At5g23250 AT5G23250	Arabidopsis thaliana (Mouse-ear cress)	297
F4KGY8	F4KGY8_ARATH	UTP--glucose-1-phosphate uridylyltransferase (EC	UGP2 AtUGP2 MKP11.26 MKP11_26 At5g17310 AT5G1	Arabidopsis thaliana (Mouse-ear cress)	390
Q9SIV9	PPA10_ARATH	Purple acid phosphatase 10 (EC 3.1.3.2)	PAP10 AT11 At2g16430 F16F14.7	Arabidopsis thaliana (Mouse-ear cress)	468
Q08770	RL102_ARATH	60S ribosomal protein L10-2 (Wilms tumor suppre	RPL10B At1g26910 T2P11.10	Arabidopsis thaliana (Mouse-ear cress)	221
Q9M5K2	DLDH2_ARATH	Dihydrolipoyl dehydrogenase 2, mitochondrial (Atr	LPD2 At3g17240 MGD8.7	Arabidopsis thaliana (Mouse-ear cress)	507
F4JM86	F4JM86_ARATH	Catalase (EC 1.11.1.6)	CAT2 CATALASE catalase 2 At4g35090 AT4G35090 T12	Arabidopsis thaliana (Mouse-ear cress)	474
Q8VZH2	APM1_ARATH	Aminopeptidase M1 (EC 3.4.11.2) (Alpha-aminoac	APM1 At4g33090 F4I10_20	Arabidopsis thaliana (Mouse-ear cress)	879
Q9SGE0	AXS2_ARATH	UDP-D-apiose/UDP-D-xylose synthase 2	AXS2 At1g08200 T23G18.6	Arabidopsis thaliana (Mouse-ear cress)	389
Q84Y18	CXIP4_ARATH	CAX-interacting protein 4	CXIP4 At2g28910 F8N16.20	Arabidopsis thaliana (Mouse-ear cress)	332
Q9FM55	FDL41_ARATH	Putative F-box/FBD/LRR-repeat protein At5g6297	At5g62970 MJH22.2	Arabidopsis thaliana (Mouse-ear cress)	449
O82662	SUCB_ARATH	Succinate--CoA ligase [ADP-forming] subunit beta	At2g20420 F11A3.3	Arabidopsis thaliana (Mouse-ear cress)	421
Q9LJE4	CPNB2_ARATH	Chaperonin 60 subunit beta 2, chloroplastic (CPN-	CPN60B2 Cpn60-B(2) At3g13470 MRP15.11	Arabidopsis thaliana (Mouse-ear cress)	596
Q9LD43	ACCA_ARATH	Acetyl-coenzyme A carboxylase carboxyl transfera	CAC3 At2g38040 T8P21.5	Arabidopsis thaliana (Mouse-ear cress)	769
A8MQA1	A8MQA1_ARATH	60S ribosomal protein L13	BBC1 40S RIBOSOMAL PROTEIN ATBBC1 breast basic	Arabidopsis thaliana (Mouse-ear cress)	204
Q9M9K1	PMG2_ARATH	Probable 2,3-bisphosphoglycerate-independent pl	PGM2 At3g08590 F17O14.6	Arabidopsis thaliana (Mouse-ear cress)	560
F4JQ55	F4JQ55_ARATH	Chaperone protein htpG family protein	SHD AtHsp90-7 AtHsp90.7 HEAT SHOCK PROTEIN 90-7	Arabidopsis thaliana (Mouse-ear cress)	823
O04090	FER1_ARATH	Ferredoxin-1, chloroplastic (AtFd1)	FD1 PETF2 At1g10960 T19D16.12	Arabidopsis thaliana (Mouse-ear cress)	148
Q8LPJ5	ICDHP_ARATH	Isocitrate dehydrogenase [NADP], chloroplastic/m	At5g14590 T15N1.80	Arabidopsis thaliana (Mouse-ear cress)	485
Q9SS17	RS241_ARATH	40S ribosomal protein S24-1	RPS24A At3g04920 T9J14.13	Arabidopsis thaliana (Mouse-ear cress)	133
Q9SIB9	ACO3M_ARATH	Aconitate hydratase 3, mitochondrial (Aconitase 3	ACO3 At2g05710 T3P4.5	Arabidopsis thaliana (Mouse-ear cress)	990
Q5GM68	CAPP2_ARATH	Phosphoenolpyruvate carboxylase 2 (AtPPC2) (PE	PPC2 At2g42600 F14N22.13	Arabidopsis thaliana (Mouse-ear cress)	963
Q96254	GDI1_ARATH	Guanosine nucleotide diphosphate dissociation in	GDI1 At2g44100 F6E13.23	Arabidopsis thaliana (Mouse-ear cress)	445
Q9S7B5	THRC1_ARATH	Threonine synthase 1, chloroplastic (EC 4.2.3.1) (	TS1 MTO2 At4g29840 F27B13.80	Arabidopsis thaliana (Mouse-ear cress)	526
Q9C522	ACLB1_ARATH	ATP-citrate synthase beta chain protein 1 (ATP-cit	ACLB-1 At3g06650 F5E6.2 T8E24.7	Arabidopsis thaliana (Mouse-ear cress)	608
F4IGK5	F4IGK5_ARATH	NAD(P)-linked oxidoreductase superfamily protein	At2g21250 AT2G21250 F3K23.1 F3K23_1	Arabidopsis thaliana (Mouse-ear cress)	238
O04487	EF1G1_ARATH	Probable elongation factor 1-gamma 1 (EF-1-gam	At1g09640 F21M12.3	Arabidopsis thaliana (Mouse-ear cress)	414
Q9SRV5	METE2_ARATH	5-methyltetrahydropteroyltriglutamate--homocystei	MS2 At3g03780 F20H23.19	Arabidopsis thaliana (Mouse-ear cress)	765
Q9M1X0	RRFC_ARATH	Ribosome-recycling factor, chloroplastic (RRF) (Cp	RRF At3g63190 F16M2_40	Arabidopsis thaliana (Mouse-ear cress)	275
Q9LXG1	RS91_ARATH	40S ribosomal protein S9-1	RPS9B At5g15200 F8M21_90	Arabidopsis thaliana (Mouse-ear cress)	198
Q9LK23	G6PD5_ARATH	Glucose-6-phosphate 1-dehydrogenase 5, cytopla	G6PD5 ACG9 At3g27300 K17E12.12	Arabidopsis thaliana (Mouse-ear cress)	516
F4JC86	F4JC86_ARATH	Ubiquitin family protein	DDI1 DNA-damage inducible 1 At3g13235 AT3G13235	Arabidopsis thaliana (Mouse-ear cress)	413
Q9LYR4	Q9LYR4_ARATH	transaldolase (EC 2.2.1.2)	TRA2 transaldolase 2 At5g13420 AT5G13420 T22N19.70	Arabidopsis thaliana (Mouse-ear cress)	438
Q9FFR3	6PGD3_ARATH	6-phosphogluconate dehydrogenase, decarboxyla	PGD3 At5g41670 MBK23.20	Arabidopsis thaliana (Mouse-ear cress)	487
P29517	TBB9_ARATH	Tubulin beta-9 chain (Beta-9-tubulin)	TUBB9 TUB9 At4g20890 T13K14.50	Arabidopsis thaliana (Mouse-ear cress)	444
P39207	NDK1_ARATH	Nucleoside diphosphate kinase 1 (EC 2.7.4.6) (Nu	NDK1 NDPK1 At4g09320 T30A10.80	Arabidopsis thaliana (Mouse-ear cress)	149
F4IDD6	F4IDD6_ARATH	phenylalanine--tRNA ligase (EC 6.1.1.20) (Phenyla	At1g72550 AT1G72550 F28P22.26 F28P22_26	Arabidopsis thaliana (Mouse-ear cress)	584
Q9MAH0	CAPP1_ARATH	Phosphoenolpyruvate carboxylase 1 (AtPPC1) (PE	PPC1 p107 At1g53310 F12M16.21	Arabidopsis thaliana (Mouse-ear cress)	967
P93736	SYVM1_ARATH	Valine--tRNA ligase, mitochondrial 1 (EC 6.1.1.9) (	TWN2 At1g14610 T5E21.11	Arabidopsis thaliana (Mouse-ear cress)	1108
Q9LZ66	SIR_ARATH	Assimilatory sulfite reductase (ferredoxin), chlorop	SIR At5g04590 T32M21.190	Arabidopsis thaliana (Mouse-ear cress)	642
Q84TI2	Q84TI2_ARATH	phosphoribosylaminoimidazole carboxylase (EC 4	At2g37690 AT2G37690 F13M22.19 F13M22_19	Arabidopsis thaliana (Mouse-ear cress)	642
O80585	MTHR2_ARATH	Methylenetetrahydrofolate reductase (NADH) 2 (A	MTHFR2 At2g44160 F6E13.29	Arabidopsis thaliana (Mouse-ear cress)	594
Q9FNN5	NDUV1_ARATH	NADH dehydrogenase [ubiquinone] flavoprotein 1	At5g08530 MAH20.9	Arabidopsis thaliana (Mouse-ear cress)	486
Q9SL67	PRS4B_ARATH	26S proteasome regulatory subunit 4 homolog B (	RPT2B At2g20140 T2G17.6	Arabidopsis thaliana (Mouse-ear cress)	443
P24636	TBB4_ARATH	Tubulin beta-4 chain (Beta-4-tubulin)	TUBB4 TUB4 At5g44340 K9L2.12	Arabidopsis thaliana (Mouse-ear cress)	444
Q9C5I1	USP_ARATH	UDP-sugar pyrophosphorylase (AtUSP) (EC 2.7.7	USP At5g52560 F6N7.4	Arabidopsis thaliana (Mouse-ear cress)	614
O23254	GLYC4_ARATH	Serine hydroxymethyltransferase 4 (AtSHMT4) (E	SHM4 SHMT4 At4g13930 dl3005c FCAALL.160	Arabidopsis thaliana (Mouse-ear cress)	471
P83483	ATPBM_ARATH	ATP synthase subunit beta-1, mitochondrial (EC 7	At5g08670 T2K12.11	Arabidopsis thaliana (Mouse-ear cress)	556
Q9SRT9	RGP1_ARATH	UDP-arabinopyranose mutase 1 (EC 5.4.99.30) (	RGP1 At3g02230 F14P3.12	Arabidopsis thaliana (Mouse-ear cress)	357
O80576	O80576_ARATH	At2g44060 (Late embryogenesis abundant protein	late embryogenesis abundant 26 LEA26 At2g44060 AT2G	Arabidopsis thaliana (Mouse-ear cress)	325
Q9LIR4	ILVD_ARATH	Dihydroxy-acid dehydratase, chloroplastic (AthDH	DHAD At3g23940 F14O13.13	Arabidopsis thaliana (Mouse-ear cress)	608
Q9ZPS7	TMN3_ARATH	Transmembrane 9 superfamily member 3 (Endom	TMN3 EMP9 At2g01970 F14H20.4	Arabidopsis thaliana (Mouse-ear cress)	592
Q9M352	RL362_ARATH	60S ribosomal protein L36-2	RPL36B At3g53740 F5K20_40	Arabidopsis thaliana (Mouse-ear cress)	112
F4JBY2	F4JBY2_ARATH	transketolase (EC 2.2.1.1)	AtTKL1 TKL1 transketolase 1 At3g60750 AT3G60750	Arabidopsis thaliana (Mouse-ear cress)	740
P55034	PSMD4_ARATH	26S proteasome non-ATPase regulatory subunit 4	RPN10 MBP1 MCB1 At4g38630 F20M13.190 T9A14.7	Arabidopsis thaliana (Mouse-ear cress)	386
Q9SI20	EF1D2_ARATH	Elongation factor 1-delta 2 (EF-1-delta 2) (Elongat	At2g18110 F8D23.11 T27K22.2	Arabidopsis thaliana (Mouse-ear cress)	231
Q9FIX2	AIGLA_ARATH	AIG2-like protein A (EC 2.3.2.-) (AIG2-like protein)	AIG2LA At5g39720 MIJ24.180 MKM21.1 MKM21.3 MKM2	Arabidopsis thaliana (Mouse-ear cress)	165
F4II65	F4II65_ARATH	TGF-beta receptor interacting protein 1	TRIP-1 TIF3I1 TRIP1 At2g46280 AT2G46280 T3F17.7	Arabidopsis thaliana (Mouse-ear cress)	254
O23290	RL36A_ARATH	60S ribosomal protein L36a	RPL36AA At3g23390 MLM24.22; RPL36AB At4g14320 d	Arabidopsis thaliana (Mouse-ear cress)	105
F4IJF0	F4IJF0_ARATH	Uncharacterized protein	At2g16015 AT2G16015	Arabidopsis thaliana (Mouse-ear cress)	111
Q94B78	GCSP1_ARATH	Glycine dehydrogenase (decarboxylating) 1, mitoc	GLDP1 GDP1 At4g33010 F26P21.130	Arabidopsis thaliana (Mouse-ear cress)	1037
F4HZV5	F4HZV5_ARATH	Uncharacterized protein	AT1G29041	Arabidopsis thaliana (Mouse-ear cress)	178
F4JSK5	F4JSK5_ARATH	peptidylprolyl isomerase (EC 5.2.1.8)	FKBP53 ATFKBP53 FK506 BINDING PROTEIN 53 At4g	Arabidopsis thaliana (Mouse-ear cress)	444
P21238	CPNA1_ARATH	Chaperonin 60 subunit alpha 1, chloroplastic (CPN	CPN60A1 Cpn60-A(2) SLP At2g28000 T1E2.8	Arabidopsis thaliana (Mouse-ear cress)	586
O04834	SAR1A_ARATH	GTP-binding protein SAR1A	SAR1A At4g02080 AGAA.4 T10M13.9	Arabidopsis thaliana (Mouse-ear cress)	193
Q9SU56	GLDH_ARATH	L-galactono-1,4-lactone dehydrogenase, mitochor	GLDH At3g47930 T17F15.200	Arabidopsis thaliana (Mouse-ear cress)	610
Q9SRX2	RL191_ARATH	60S ribosomal protein L19-1 (Protein EMBRYO DE	RPL19A EMB2386 At1g02780 F22D16.23	Arabidopsis thaliana (Mouse-ear cress)	214
P25696	ENO2_ARATH	Bifunctional enolase 2/transcriptional activator (E	ENO2 LOS2 At2g36530 F1O11.16	Arabidopsis thaliana (Mouse-ear cress)	444
P25856	G3PA1_ARATH	Glyceraldehyde-3-phosphate dehydrogenase GAF	GAPA1 GAPA At3g26650 MLJ15.4 MLJ15_5	Arabidopsis thaliana (Mouse-ear cress)	396
P49688	RS23_ARATH	40S ribosomal protein S2-3 (AtRPS2C)	RPS2C At2g41840 T11A07.6	Arabidopsis thaliana (Mouse-ear cress)	285
O04331	PHB3_ARATH	Prohibitin-3, mitochondrial (Atphb3) (Protein ENHA	PHB3 EER3 At5g40770 K1B16.2	Arabidopsis thaliana (Mouse-ear cress)	277
Q6IDC7	Q6IDC7_ARATH	At1g77550 (Tubulin-tyrosine ligase) (Uncharacteri	At1g77550 AT1G77550 T5M16.14 T5M16_14	Arabidopsis thaliana (Mouse-ear cress)	855
Q43291	RL211_ARATH	60S ribosomal protein L21-1	RPL21A At1g09590 F14J9.25; RPL21C At1g09690 F21M	Arabidopsis thaliana (Mouse-ear cress)	164
Q9LZL9	Q9LZL9_ARATH	At5g02130 (Tetratricopeptide repeat (TPR)-like su	NDP1 AT5G02130 T7H20.180 T7H20_180	Arabidopsis thaliana (Mouse-ear cress)	420
P48006	EF1D1_ARATH	Elongation factor 1-delta 1 (EF-1-delta 1) (Elongat	At1g30230 F12P21.12	Arabidopsis thaliana (Mouse-ear cress)	231
P29515	TBB7_ARATH	Tubulin beta-7 chain (Beta-7-tubulin)	TUBB7 TUB7 At2g29550 F16P2.7	Arabidopsis thaliana (Mouse-ear cress)	449
P46286	RL81_ARATH	60S ribosomal protein L8-1 (60S ribosomal proteir	RPL8A EMB2296 RPL2 At2g18020 T27K22.11	Arabidopsis thaliana (Mouse-ear cress)	258
O04130	SERA2_ARATH	D-3-phosphoglycerate dehydrogenase 2, chloropla	PGDH2 3-PGDH PGDH At1g17745 F11A6.8	Arabidopsis thaliana (Mouse-ear cress)	624
Q9SN21	Y3997_ARATH	Putative BTB/POZ domain-containing protein At3g	At3g49970 F3A4.50	Arabidopsis thaliana (Mouse-ear cress)	515
Q0WUK0	Q0WUK0_ARATH	Dentin sialophosphoprotein, putative (DUF1296) (	At3g13990 AT3G13990	Arabidopsis thaliana (Mouse-ear cress)	847
O24457	ODPA3_ARATH	Pyruvate dehydrogenase E1 component subunit a	PDH-E1 ALPHA At1g01090 T25K16.8	Arabidopsis thaliana (Mouse-ear cress)	428
Q9SJA6	RZ22A_ARATH	Serine/arginine-rich splicing factor RSZ22A (RS-co	RSZ22A RSZP22A At2g24590 F25P17.11	Arabidopsis thaliana (Mouse-ear cress)	196
F4KDU5	F4KDU5_ARATH	Ribosomal protein L4/L1 family	At5g02870 AT5G02870 F9G14.180 F9G14_180	Arabidopsis thaliana (Mouse-ear cress)	406
Q9XEX2	PRX2B_ARATH	Peroxiredoxin-2B (EC 1.11.1.25) (Glutaredoxin-de	PRXIIB TPX1 At1g65980 F12P19.14	Arabidopsis thaliana (Mouse-ear cress)	162
O50008	METE1_ARATH	5-methyltetrahydropteroyltriglutamate--homocystei	MS1 CIMS At5g17920 MPI7.9	Arabidopsis thaliana (Mouse-ear cress)	765
Q9SMT7	4CLLA_ARATH	Oxalate--CoA ligase (EC 6.2.1.8) (4-coumarate--C	AAE3 4CLL10 AMPBP3 At3g48990 T2J13.170	Arabidopsis thaliana (Mouse-ear cress)	514
P52577	IFRH_ARATH	Isoflavone reductase homolog P3 (EC 1.3.1.-)	At1g75280 F22H5.17	Arabidopsis thaliana (Mouse-ear cress)	310
Q56WH4	HDT2_ARATH	Histone deacetylase HDT2 (HD-tuins protein 2) (H	HDT2 HD2 HD2B HDA4 At5g22650 MDJ22.7	Arabidopsis thaliana (Mouse-ear cress)	306
Q05758	ILV5_ARATH	Ketol-acid reductoisomerase, chloroplastic (EC 1.1	At3g58610 F14P22.200	Arabidopsis thaliana (Mouse-ear cress)	591
O48844	PSD1A_ARATH	26S proteasome non-ATPase regulatory subunit 1	RPN2A At2g32730 F24L7.13	Arabidopsis thaliana (Mouse-ear cress)	1004
Q9LD57	PGKH1_ARATH	Phosphoglycerate kinase 1, chloroplastic (EC 2.7.	PGK1 At3g12780 MBK21.15	Arabidopsis thaliana (Mouse-ear cress)	481
F4KGV2	F4KGV2_ARATH	G-box regulating factor 6	GRF6 14-3-3LAMBDA 14-3-3lambda AFT1 G-box regulat	Arabidopsis thaliana (Mouse-ear cress)	246
Q9FFT4	PDC2_ARATH	Pyruvate decarboxylase 2 (AtPDC2) (EC 4.1.1.1)	PDC2 At5g54960 MBG8.23	Arabidopsis thaliana (Mouse-ear cress)	607
Q8H0V3	LGUL_ARATH	Lactoylglutathione lyase (EC 4.4.1.5) (Aldoketomu	At1g08110 T6D22.20	Arabidopsis thaliana (Mouse-ear cress)	185
Q9SYT0	ANXD1_ARATH	Annexin D1 (AnnAt1) (Annexin A1)	ANN1 ANNAT1 ANX23-ATH ATOXY5 OXY5 At1g35720 F	Arabidopsis thaliana (Mouse-ear cress)	317
Q949P2	COPDA_ARATH	Probable cytosolic oligopeptidase A (EC 3.4.24.70	CYOP TOP2 At5g10540 F12B17.110	Arabidopsis thaliana (Mouse-ear cress)	701
Q94JX9	NACA2_ARATH	Nascent polypeptide-associated complex subunit a	At3g49470 T9C5.70	Arabidopsis thaliana (Mouse-ear cress)	217
Q93ZM7	CH60C_ARATH	Chaperonin CPN60-like 2, mitochondrial (HSP60-l	At3g13860 MCP4.9	Arabidopsis thaliana (Mouse-ear cress)	572
Q42290	MPPB_ARATH	Probable mitochondrial-processing peptidase sub	MPPbeta At3g02090 F1C9.12	Arabidopsis thaliana (Mouse-ear cress)	531
Q9FWA3	6PGD2_ARATH	6-phosphogluconate dehydrogenase, decarboxyla	PGD2 At3g02360 F11A12.5	Arabidopsis thaliana (Mouse-ear cress)	486
Q9LYE9	Q9LYE9_ARATH	S-protein homolog	T22P22_210 At5g11820	Arabidopsis thaliana (Mouse-ear cress)	175
A8MS28	A8MS28_ARATH	Ribosomal L27e protein family	DL3545W AT4G15000 FCAALL.99	Arabidopsis thaliana (Mouse-ear cress)	131
A8MQR4	A8MQR4_ARATH	60S acidic ribosomal protein P0	At3g09200 AT3G09200	Arabidopsis thaliana (Mouse-ear cress)	287
Q9MAB3	NOP5B_ARATH	Probable nucleolar protein 5-2 (MAR-binding NOP	NOP5-2 NOP58-2 At3g05060 T12H1.2	Arabidopsis thaliana (Mouse-ear cress)	533
Q9C7M2	Q9C7M2_ARATH	Uncharacterized protein F14C21.55 (Zinc finger (F	At1g55040 AT1G55040 F14C21.55 F14C21_55	Arabidopsis thaliana (Mouse-ear cress)	849

A8MQK3	A8MQK3_ARATH	Malate dehydrogenase (EC 1.1.1.37)	mMDH2 At3g15020 AT3G15020	Arabidopsis thaliana (Mouse-ear cress)	316
Q42560	ACO1_ARATH	Aconitate hydratase 1 (Aconitase 1) (EC 4.2.1.3) (	ACO1 ACO At4g35830 F4B14.100	Arabidopsis thaliana (Mouse-ear cress)	898
Q9LDE3	FBK9_ARATH	F-box/kelch-repeat protein At1g23390	At1g23390 F26F24.26 F28C11.3	Arabidopsis thaliana (Mouse-ear cress)	394
Q9SAJ4	PGKY3_ARATH	Phosphoglycerate kinase 3, cytosolic (EC 2.7.2.3)	PGK3 At1g79550 T8K14.3	Arabidopsis thaliana (Mouse-ear cress)	401
P49689	RS30_ARATH	40S ribosomal protein S30	RPS30A At2g19750 F6F22.22; RPS30B At4g29390 F17/	Arabidopsis thaliana (Mouse-ear cress)	62
Q39043	BIP2_ARATH	Heat shock 70 kDa protein BIP2 (Heat shock 70 k	BIP2 BIP HSP70-12 MED37_6 MED37F At5g42020 MJC:	Arabidopsis thaliana (Mouse-ear cress)	668
Q9LZH9	RL7A2_ARATH	60S ribosomal protein L7a-2	RPL7AB At3g62870 F26K9_300	Arabidopsis thaliana (Mouse-ear cress)	256
Q2V3X4	Q2V3X4_ARATH	Ribosomal protein L4/L1 family	At3g09630 AT3G09630	Arabidopsis thaliana (Mouse-ear cress)	405
P47999	CYSKP_ARATH	Cysteine synthase, chloroplastic/chromoplastic (E	OASB At2g43750 F18O19.14	Arabidopsis thaliana (Mouse-ear cress)	392
Q9ZRW8	GSTUJ_ARATH	Glutathione S-transferase U19 (AtGSTU19) (EC 2	GSTU19 GST8 At1g78380 F3F9.11	Arabidopsis thaliana (Mouse-ear cress)	219
O80852	GSTF9_ARATH	Glutathione S-transferase F9 (AtGSTF9) (EC 2.5.:	GSTF9 GLUTTR GSTF7 Phi9 At2g30860 F7F1.7	Arabidopsis thaliana (Mouse-ear cress)	215
Q9FFC0	H2B10_ARATH	Histone H2B.10 (HTB2)	At5g22880 MRN17.11	Arabidopsis thaliana (Mouse-ear cress)	145
Q9M8N2	RTL1_ARATH	Ribonuclease 3-like protein 1 (Ribonuclease III-like	RTL1 At1g80650 T21F11.2	Arabidopsis thaliana (Mouse-ear cress)	198
Q9LDZ0	HSP7J_ARATH	Heat shock 70 kDa protein 10, mitochondrial (Cha	HSP70-10 HSC70-5 HSCA2 MTHSC70-2 At5g09590 F17	Arabidopsis thaliana (Mouse-ear cress)	682
F4JDC2	F4JDC2_ARATH	YTH domain-containing family protein	ECT2 At3g13460 AT3G13460	Arabidopsis thaliana (Mouse-ear cress)	664
P25853	BAM5_ARATH	Beta-amylase 5 (AtBeta-Amy) (EC 3.2.1.2) (1,4-al	BAM5 BMY1 RAM1 At4g15210 dl3650c FCAALL.97	Arabidopsis thaliana (Mouse-ear cress)	498
Q9SU89	Q9SU89_ARATH	Fanconi anemia group E protein FANCE protein (U	At4g29560 AT4G29560 T16L4.70 T16L4_70	Arabidopsis thaliana (Mouse-ear cress)	493
Q9LK36	SAHH2_ARATH	Adenosylhomocysteinase 2 (AdoHcyase 2) (EC 3.	SAHH2 At3g23810 MYM9.16 MYM9_15	Arabidopsis thaliana (Mouse-ear cress)	485
Q9LYK9	RS263_ARATH	40S ribosomal protein S26-3	RPS26C At3g56340 F18O21_300	Arabidopsis thaliana (Mouse-ear cress)	130
P19171	CHIB_ARATH	Basic endochitinase B (EC 3.2.1.14) (Pathogenes	CHI-B PR3 At3g12500 MQC3.32 T2E22.18 T2E22_119	Arabidopsis thaliana (Mouse-ear cress)	335
Q944H0	PEAM2_ARATH	Phosphomethylethanolamine N-methyltransferase	NMT2 PMEAMT At1g48600 T1N15.22/T1N15.23 T1N15_	Arabidopsis thaliana (Mouse-ear cress)	491
Q84VW9	CAPP3_ARATH	Phosphoenolpyruvate carboxylase 3 (AtPPC3) (PI	PPC3 PEPC PPC At3g14940 K15M2.8	Arabidopsis thaliana (Mouse-ear cress)	968
B3H700	B3H700_ARATH	E3 ubiquitin-protein ligase (EC 2.3.2.26)	KAK AT4G38610 KAKTUS UBIQUITIN-PROTEIN LIGASE	Arabidopsis thaliana (Mouse-ear cress)	1794
Q9SJ36	RS172_ARATH	40S ribosomal protein S17-2	RPS17B At2g05220 F5G3.12	Arabidopsis thaliana (Mouse-ear cress)	140
Q9FY65	RS153_ARATH	40S ribosomal protein S15-3	RPS15C At5g09500 T5E8_300	Arabidopsis thaliana (Mouse-ear cress)	150
F4J5H1	F4J5H1_ARATH	Alpha/beta-Hydrolases superfamily protein	AT3G30380	Arabidopsis thaliana (Mouse-ear cress)	377
Q9SZJ5	GLYM1_ARATH	Serine hydroxymethyltransferase 1, mitochondrial	SHM1 SHMT1 STM At4g37930 F20D10.50	Arabidopsis thaliana (Mouse-ear cress)	517
Q9FNA9	PDAT1_ARATH	Phospholipid:diacylglycerol acyltransferase 1 (AtP	PDAT1 PDAT At5g13640 MSH12.10 T6I14.2	Arabidopsis thaliana (Mouse-ear cress)	671
F4HPW9	F4HPW9_ARATH	Stress response protein	At1g60640 AT1G60640 F8A5.16 F8A5_16	Arabidopsis thaliana (Mouse-ear cress)	298
Q944K2	OST48_ARATH	Dolichyl-diphosphooligosaccharide--protein glycos	OST48 DGL1 At5g66680 MSN2.7	Arabidopsis thaliana (Mouse-ear cress)	437
Q93ZN9	DAPAT_ARATH	LL-diaminopimelate aminotransferase, chloroplast	DAP AGD2 At4g33680 T16L1.170	Arabidopsis thaliana (Mouse-ear cress)	461
P25858	G3PC1_ARATH	Glyceraldehyde-3-phosphate dehydrogenase GAF	GAPC1 GAPC GAPDH At3g04120 T6K12.26	Arabidopsis thaliana (Mouse-ear cress)	338
O22256	PME20_ARATH	Probable pectinesterase/pectinesterase inhibitor 2	PME20 ARATH20 At2g47550 T30B22.15	Arabidopsis thaliana (Mouse-ear cress)	560
O80949	OTU8_ARATH	Putative OVARIAN TUMOR DOMAIN-containing d	OTU8 At2g39320 T16B24.4	Arabidopsis thaliana (Mouse-ear cress)	189
Q9FX54	G3PC2_ARATH	Glyceraldehyde-3-phosphate dehydrogenase GAF	GAPC2 GAPDH At1g13440 T6J4.17	Arabidopsis thaliana (Mouse-ear cress)	338
O23515	RL151_ARATH	60S ribosomal protein L15-1	RPL15A At4g16720 dl4385c FCAALL.416	Arabidopsis thaliana (Mouse-ear cress)	204
O64743	BBE15_ARATH	Berberine bridge enzyme-like 15 (AtBBE-like 15) (	MEE23 EDA28 At2g34790 F19I3.2	Arabidopsis thaliana (Mouse-ear cress)	532
Q9LKR3	BIP1_ARATH	Heat shock 70 kDa protein BIP1 (Heat shock 70 k	BIP1 HSP70-11 MED37_5 MED37A At5g28540 T26D3.1	Arabidopsis thaliana (Mouse-ear cress)	669
Q9S9N1	HSP7E_ARATH	Heat shock 70 kDa protein 5 (Heat shock protein 7	HSP70-5 HSP70B At1g16030 T24D18.14	Arabidopsis thaliana (Mouse-ear cress)	646
Q9FKA5	Y5957_ARATH	Uncharacterized protein At5g39570	At5g39570 MIJ24_40	Arabidopsis thaliana (Mouse-ear cress)	381
Q9LMX7	C78A5_ARATH	Cytochrome P450 78A5 (EC 1.14.-.-) (Protein KLU	CYP78A5 KLU At1g13710 F21F23.15	Arabidopsis thaliana (Mouse-ear cress)	517
O23653	AK2_ARATH	Aspartokinase 2, chloroplastic (EC 2.7.2.4) (Aspar	AK2 AK-LYS2 CARAB-AK-LYS At5g14060 MUA22.6	Arabidopsis thaliana (Mouse-ear cress)	544

Supplemental Table 2. Selected candidates of identified *S*-nitrosylated proteins in plant extracts treated with several NO donors

Protein	Acc. No	Organism	Condition	*S*-nitrosylome
**Stress- and Redox-related proteins**				
Glutathione S-transferase U19 (GSTU19)	Q9ZRW8	*Arabidopsis thaliana*	GSNO/NO<br>NO_2-Ln	Lindermayr *et al*., 2005<br>This study
Heat shock protein 90-3 (HSP90-3)	P51818	*Arabidopsis thaliana*	GSNO/NO<br>NO_2-Ln	Lindermayr *et al*., 2005<br>This study
Glutathione peroxidase (GPX)	O48646	*Arabidopsis thaliana*	GSNO/NO<br>NO_2-Ln	Lindermayr *et al*., 2005<br>This study
Peroxiredoxin-2F (PRXIIF)	Q9M7T0	*Arabidopsis thaliana*	GSNO/NO<br>NO_2-Ln	Lindermayr *et al*., 2005<br>This study
Peroxiredoxin-2B (PRXIIB)	Q9XEX2	*Arabidopsis thaliana*	GSNO/NO<br>NO_2-Ln	Lindermayr *et al*., 2005<br>This study
Ascorbate peroxidase (APX)	Q8H9F0<br>Q09Y78	*Solanum tuberosum*	GSNO	Kato *et al*., 2012
	F4HU93	*Arabidopsis thaliana*	NO_2-Ln	This study
Catalase (CAT)	P55312	*Solanum tuberosum*	GSNO	Kato *et al*., 2012
	F4JM86	*Arabidopsis thaliana*	NO_2-Ln	This study
Ferredoxin (Fed)	93XJ9	*Solanum tuberosum*	GSNO	Kato *et al*., 2012
	O04090	*Arabidopsis thaliana*	NO_2-Ln	This study
Thioredoxin (TRXh)	P29449	*Solanum tuberosum*	GSNO	Kato *et al*., 2012
	Q42403<br>Q39241<br>F4IL52<br>Q9LYA5	*Arabidopsis thaliana*	NO_2-Ln	This study

Supplemental Table 2. Selected candidates of identified *S*-nitrosylated proteins in plant extracts treated with several NO donors

Dehydroascorbate reductase (DHAR)	A2ICR9<br>Q3HVN5	*Solanum tuberosum*	GSNO	Kato *et al*., 2012
	Q4VDN8<br>Q9FRL8	*Arabidopsis thaliana*	NO_2-Ln	This study
Malate dehydrogenase (MDH)	Q9XQP4	*Nicotiana tabacum*	GSNO	Kato *et al*., 2012
	P93819<br>P57106<br>A8MQK3	*Arabidopsis thaliana*	NO_2-Ln	This study
Catalase (CAT)	F4HUL6	*Arabidopsis thaliana*	GSNO<br>NO_2-Ln	Palmieri *et al*., 2010<br>This study
	F4JM86	*Arabidopsis thaliana*	NO_2-Ln	This study
**Signalling/Regulating proteins**				
Elongation factors (EFs)	Q8GTY0<br>Q9ASR1<br>Q9SCX3<br>Q9FVT2	*Arabidopsis thaliana*	GSNO/NO	Lindermayr *et al*., 2005
	Q8H9C0<br>Q2VCK4<br>Q6L4C2	*Solanum tuberosum*	GSNO	Kato *et al*., 2012
	P17745<br>Q9ASR1<br>Q9ZT91<br>P0DH99<br>O04487<br>Q9SI20<br>P48006	*Arabidopsis thaliana*	NO_2-Ln	This study
Initiation factors (IFs)	P41376<br>Q93VP3	*Arabidopsis thaliana*	GSNO/NO	Lindermayr *et al*., 2005
	P56335	*Sonalum tuberosum*	GSNO	Kato *et al*., 2012

Supplemental Table 2. Selected candidates of identified *S*-nitrosylated proteins in plant extracts treated with several NO donors

Initiation factors (IFs)	Q9LD55<br>P41377<br>A8MRZ7<br>Q9SIZ2<br>Q9FKI2<br>Q94A52<br>Q9C5Z3	*Arabidopsis thaliana*	NO_2-Ln	This study
**Cytoskeleton proteins**				
Actin (ACT)	P53492	*Arabidopsis thaliana*	GSNO/NO<br>NO_2-Ln	Lindermayr *et al*., 2005<br>This study
	C7F8M9	*Sonalum tuberosum*	GSNO	Kato *et al*., 2012
	P53494<br>Q96292<br>P0CJ46<br>Q9LV35	*Arabidopsis thaliana*	NO_2-Ln	This study
	A0A0A0KJ21	*Cucumis sativus*	GSNO	Niu *et al*., 2019
Actin depolymerizing factor (ADF)	Q9ZSK4	*Arabidopsis thaliana*	GSNO/NO<br>NO_2-Ln	Lindermayr *et al*., 2005<br>This study
	Q39251	*Arabidopsis thaliana*	NO_2-Ln	This study
Tubulin (TUB)	P29511	*Arabidopsis thaliana*	GSNO/NO<br>NO_2-Ln	Lindermayr *et al*., 2005<br>This study
	P24636		GSNO/NO<br>NO_2-Ln	Lindermayr *et al*., 2005<br>This study

Supplemental Table 2. Selected candidates of identified *S*-nitrosylated proteins in plant extracts treated with several NO donors

Tubulin (TUB)	P29514<br>P11139<br>Q56YW9<br>P29516<br>P12411<br>Q56WH1<br>P29517<br>P29515		NO_2-Ln	This study
	A0A0A0K6A8<br>A0A0A0LCY8	*Cucumis sativus*	GSNO	Niu *et al*., 2019
Annexin (ANN)	Q9SYT0	*Arabidopsis thaliana*	GSNO/NO<br>NO_2-Ln	Lindermayr *et al*., 2005<br>This study
	Q9XEE2		NO_2-Ln	This study
**Metabolic enzymes**				
Fructose 1,6-biphosphate aldolase (FBA)	Q9LF98	*Arabidopsis thaliana*	GSNO/NO<br>NO_2-Ln	Lindermayr *et al*., 2005<br>This study
	Q2PYX3<br>B0FPD8	*Solanum tuberosum*	GSNO	Kato *et al*., 2012
	Q9SJQ9<br>Q944G9	*Arabidopsis thaliana*	NO_2-Ln	This study
	A0A0A0KKE4	*Cucumis sativus*	GSNO	Niu *et al*., 2019
Triosephosphate isomerase (TPI)	P48491	*Arabidopsis thaliana*	GSNO/NO<br>NO_2-Ln	Lindermayr *et al*., 2005<br>This study
	Q38JI4	*Solanum tuberosum*	GSNO	Kato *et al*., 2012
	Q9SKP6	*Arabidopsis thaliana*	NO_2-Ln	This study
	A0A0A0LJ13	*Cucumis sativus*	GSNO	Niu *et al*., 2019

Supplemental Table 2. Selected candidates of identified *S*-nitrosylated proteins in plant extracts treated with several NO donors

Glyceraldehyde 3-phosphate dehydrogenase (GAPDH)	P25858<br>P25856	*Arabidopsis thaliana*	GSNO/NO<br>NO_2-Ln	Lindermayr *et al*., 2005<br>This study
	Q43833	*Solanum tuberosum*	GSNO	Kato *et al*., 2012
	Q5E924<br>Q5E924<br>P25857<br>Q9FX54	*Arabidopsis thaliana*	NO_2-Ln	This study
	A0A0A0K8C1	*Cucumis sativus*	GSNO	Niu *et al*., 2019
Enolase (ENO)	P25696	*Arabidopsis thaliana*	GSNO/NO<br>NO_2-Ln	Lindermayr *et al*., 2005<br>This study
	Q9C9C4		NO_2-Ln	This study
Phosphoglycerate kinase (PGK)	Q9SAJ4	*Arabidopsis thaliana*	GSNO/NO<br>NO_2-Ln	Lindermayr *et al*., 2005<br>This study
	O81394	*Solanum tuberosum*	GSNO	Kato *et al*., 2012
	A0A0A0KEF3	*Cucumis sativus*		Niu *et al*., 2019
Aconitase (ACO)	Q9SIB9	*Arabidopsis thaliana*	GSNO/NO<br>NO_2-Ln	Lindermayr *et al*., 2005<br>This study
	F4IQ61<br>Q42560		NO_2-Ln	This study
	A0A0A0KHD6	*Cucumis sativus*	GSNO	Niu *et al*., 2019
S-adenosylmethionine synthase (SAM)	Q9LUT2	*Arabidopsis thaliana*	GSNO/NO<br>NO_2-Ln	Lindermayr *et al*., 2005<br>This study
	P23686<br>P17562<br>Q9SJL8		NO_2-Ln	This study
Adenosylhomocysteinase (SAHH)	O23255	*Arabidopsis thaliana*	GSNO/NO<br>NO_2-Ln	Lindermayr *et al*., 2005<br>This study

Supplemental Table 2. Selected candidates of identified *S*-nitrosylated proteins in plant extracts treated with several NO donors

Adenosylhomocysteinase (SAHH)	Q9LK36		NO_2-Ln	This study
	A0A0A0LNE3	*Cucumis sativus*	GSNO	Niu *et al*., 2019
Methionine synthase (MS)	O50008	*Arabidopsis thaliana*	GSNO/NO<br>NO_2-Ln	Lindermayr *et al*., 2005<br>This study
	Q9SRV5		NO_2-Ln	This study
	A0A0A0LEZ3	*Cucumis sativus*	GSNO	Niu *et al*., 2019
Cysteine synthase (CYS)	P47998	*Arabidopsis thaliana*	GSNO/NO	Lindermayr *et al*., 2005
	Q9FS26	*Solanum tuberosum*	GSNO	Kato *et al*., 2012
	Q43725<br>Q9S757<br>P47999	*Arabidopsis thaliana*	NO_2-Ln	This study
ATP synthase (ATP)	P56757<br>P19366	*Arabidopsis thaliana*	GSNO/NO	Lindermayr *et al*., 2005
	Q27S65	*Solanum tuberosum*	GSNO	Kato *et al*., 2012
	P83483	*Arabidopsis thaliana*	NO_2-Ln	This study
	P92549	*Arabidopsis thaliana*	GSNO<br>NO_2-Ln	Palmieri *et al*., 2010<br>This study
	G3EIZ8	*Cucumis sativus*	GSNO	Niu *et al*., 2019
Transaldolase	O04894	*Solanum tuberosum*	GSNO	Kato *et al*., 2012
	Q9LYR4	*Arabidopsis thaliana*	NO_2-Ln	This study
Fructokinase (PPK)	P37829	*Solanum tuberosum*	GSNO	Kato *et al*., 2012
	Q9SYP2<br>Q8W4M5	*Arabidopsis thaliana*	NO_2-Ln	This study
Alcohol dehydrogenase (ADH)	P14673	*Solanum tuberosum*	GSNO	Kato *et al*., 2012

Supplemental Table 2. Selected candidates of identified *S*-nitrosylated proteins in plant extracts treated with several NO donors

Alcohol dehydrogenase (ADH)	Q9CAI3<br>P06525<br>Q9LK96<br>A1L4Y4	*Arabidopsis thaliana*	NO_2-Ln	This study
Glycine dehydrogenase (decarboxylating)	O80988<br>Q94B78	*Arabidopsis thaliana*	GSNO<br>NO_2-Ln	Palmieri *et al*., 2010<br>This study
Serine hydroxymetyltransferase	Q9SZJ5	*Arabidopsis thaliana*	GSNO<br>NO_2-Ln	Palmieri *et al*., 2010<br>This study
Dihydrolipoyl dehydrogenase	Q9M5K3<br>Q9M5K2	*Arabidopsis thaliana*	GSNO<br>NO_2-Ln	Palmieri *et al*., 2010<br>This study
	A0A0A0KG56	*Cucumis sativus*	GSNO	Niu *et al*., 2019
**Photosynthetic-involved proteins**				
Rubisco large chain (RBCL)	O03042	*Arabidopsis thaliana*	GSNO/NO<br>NO_2-Ln	Lindermayr *et al*., 2005<br>This study
	P25079	*Solanum tuberosum*	GSNO	Kato *et al*., 2012
	A0A0A0KAU8	*Cucumis sativus*		Niu *et al*., 2019
Rubisco small chain (RBCS)	P10795	*Arabidopsis thaliana*	GSNO/NO<br>NO_2-Ln	Lindermayr *et al*., 2005<br>This study
	P26576	*Solanum tuberosum*	GSNO	Kato *et al*., 2012
Rubisco activase (RCA)	P10896	*Arabidopsis thaliana*	GSNO/NO<br>NO_2-Ln	Lindermayr *et al*., 2005<br>This study
	O49074	*Solanum lycopersicum*	GSNO	Kato *et al*., 2012
Phosphoribulokinase (PRK)	P25697	*Arabidopsis thaliana*	GSNO<br>NO_2-Ln	Kato *et al*., 2012<br>This study
	A0A0A0L987	*Cucumis sativus*	GSNO	Niu *et al*., 2019

Supplemental Table 2. Selected candidates of identified *S*-nitrosylated proteins in plant extracts treated with several NO donors

**Defense-related proteins**				
Chitinase/ Pathogenesis-related proteins	O81144<br>Q8LRU6	*Solanum tuberosum*	GSNO	Kato *et al*., 2012
	P19171<br>Q8GUG1	*Arabidopsis thaliana*	NO_2-Ln	This study
Metacaspase	Q8H272	*Solanum tuberosum*	GSNO	Kato *et al*., 2012
	O64517	*Arabidopsis thaliana*	NO_2-Ln	This study

References

Kato H, Takemoto D, Kawakita K. 2012. Proteomic analysis of *S*-nitrosylated proteins in potato plant. *Physiologia Plantarum* **148**(3):371-86.

Lindermayr C, Saalbach G, Durner J. 2005. Proteomic identification of S-nitrosylated proteins in Arabidopsis. *Plant Physiology* **137**(3): 921-930.

Niu L, Yu J, Liao W, Xie J, Yu J, Lv J, Xiao X, Hu L, Wu Y. 2019. Proteomic investigation of *S*-nitrosylated proteins during NO-induced adventitious rooting of cucumber. *Int J Mol Sci* **20**(21): 5363

Palmieri MC, Lindermayr C, Bauwe H, Steinhauser C, Durner J. 2010. Regulation of plant glycine decarboxylase by *S*-nitrosylation and glutathionylation. *Plant Physiology* **152**(3):1514-28.

Supplementary Table S3. List of primers for RT-qPCR.

Primer	Sequence (5’-3’)
GSNOR1 FW	TGACTATATGGGTCCTCTCTGCTTTAATCTATGTG
GSNOR1 RV	AAACGAGACACACAAGAGTAGAGTAGTCTGTTGC
