# Supplementary material for: Nitro-fatty acids modulate germination onset through S-nitrosothiol metabolism
Source: Plant Physiol. 2025 Jan 25;197(2):kiaf038. doi: 10.1093/plphys/kiaf038 (PMC11831805; doi:10.1093/plphys/kiaf038)
Supplement: kiaf038_Supplementary_Data [file pp.02192.2024-s06.pdf › pp.02192.2024-s06.pdf]

Plant Physiology®

Nitro-fatty acids as modulators of germination onset through S-nitrosothiol metabolism

Capilla Pérez, Juan Begara-Morales, Maria de las Nieves Padilla-Serrano, Mounira Chaki, Beatriz Calvo, Alfonso Carreras, Lorena Aranda-Caño, Manuel Melguizo, Raquel Valderrama, Maria Inmaculada Sánchez Vicente, Oscar Lorenzo, and Juan Barroso

Review Timeline:

Submission Date:	17-Oct-2023
Editorial Decision:	23-Nov-2023
Resubmission Received:	29-Jul-2024
Editorial Decision:	25-Aug-2024
Revision Received:	06-Sep-2024
Editorial Decision:	27-Sep-2024
Revision Received:	26-Nov-2024
Accepted:	27-Nov-2024

23-Nov-2023

Prof. Dr. Juan B Barroso
Universidad de Jaen Departamento de Biologia Experimental
Experimental Biology
Jaen, Jaen 23071
Spain

RE: Nitro-fatty acids as modulators of the germination onset through the S-nitrosothiol metabolism

Dear Dr. Juan Barroso:

Thank you for giving us the opportunity to review your manuscript for publication in the Plant Physiology. The reviewers have found the manuscript of interest, but have raised a number of concerns that will require your attention. As these concerns are of some gravity and will entail substantial changes to the manuscript, you are likely to need additional time to address them. I must therefore reject the manuscript in its present form. Nonetheless, if you are able to address the points raised in review, I would welcome your resubmission of a manuscript that incorporates your responses.

Both reviewers and the editor fund that the manuscript requires attention regarding structure, detailing the methods, and data interpretation. In particular, I direct your attention to the following comments for consideration in a resubmitted manuscript:

1. Given that Ln-NO functions as NO donor, if Ln-NO S-nitrosylates GSNOR1, provide explanation about how Ln-NO activities (donor vs trans-nitrosylating agent) are regulated, as raised by reviewer #1.
2. Detail the methods, particularly regarding: (i) proteomics experiments (i.e. biological replicates, threshold used), (ii) approaches to identify proteins whose S-nitrosylation reduced by Ln-NO2 treatment, and (iii) effectiveness of thiol blocking, as indicated in the comments from reviewers #1 and #2.
3. Provide the necessary controls for western blots to support the claims, as detailed in the comments of both reviewers.
4. Compare increases in SNO levels induced by Ln-NO treatment in WT, gsnor1 mutant to investigate the relevance of SNO-GSNOR1 formation induced by Ln-NO.
5. Comparing the list of proteins in Table 1 with published s-nitrosylation proteomes, especially from studies looking at known nitrosylation targets, , as indicated in the comments of reviewer #2.
6. For proteins considered in the validation experiments, determine their nitrosylation states in the aer mutant as well as in gnsor1 and GSNOR1 overexpressing mutants, as indicated in the comments of reviewer #2.
7. Improve and streamline the Methods section and figures, as mentioned in the comments by both reviewers.
Please note that any resubmission will be treated as a new entry to the system and is likely to go out for review.

If you do have any queries or concerns, you are welcome to contact me in the first instance.

Thank you again for allowing Plant Physiology to review your work. We look forward to hearing from you soon.

Sincerely,

Editor
Plant Physiology

-- Reviewer comments:
Reviewer #1 (Comments for the Author):

The manuscript by Perez et al is aimed at uncovering mechanisms of S-nitrosylation, a protein post-translational modification mediated by the redox active molecule nitric oxide (NO). Previous reports have established nitro-linoleic acid (Ln-NO) as an endogenous nitric oxide (NO) donor. However, little is known about the targets of Ln-NO and its biological function. Here, the authors add to a body of evidence indicating that Ln-NO functions as a NO donor in cells and controls seed germination. Based on protein and proteomics analyses, it is claimed that Ln-NO targets NO turnover to affect stability of ABI5, a master regulator of seed germination.

The manuscript attempts to widen the understanding of SNO signalling in plants, particularly of nitro-fatty acids. In general, the manuscript requires some attention on structure and data interpretation - as described in more details below in the point-by-point assessment. Due to the experimental strategy used, it is difficult at times to establish causal links between Ln-NO, seed germination and the mechanism proposed.

Specific comments

Line 55-57: the manuscript aims to address how Ln-NO regulates GSNO bioavailability and SNO formation, and not GSNO biosynthesis as stated. The text should be corrected to reflect that.

Line 65-66: given that Ln-NO functions as NO donor, if Ln-NO S-nitrosylates GSNOR1 (see comments below on evidence for this claim) how are Ln-NO activities (donor vs trans-nitrosylating agent) regulated? In addition, the authors seem to fluctuate between these two non-mutually exclusive scenarios throughout the manuscript. A careful revision of the text would help clarify the message.

Throughout, details of the methodological approach are missing. This hinders data analysis and interpretation. For instance, how cells were treated in Fig1A is unclear. Were cells exposed to DTPA and NEM before addition of or along with Ln-NO? Thiol alkylation by NEM is often performed at 50C, so it is unclear how effective thiol blocking was in the assay. I am also unsure about the faint signal in the '-fluorophore' sample. If 'fluorophore' refers to the fluorescent probe DAF used in the assay, what is the difference between Fig1C and E?

The authors claim that GSNOR1 is repressed at transcriptional, protein and activity levels by Ln-NO. Although some genetic evidence is provided by using aer mutant plants, no direct evidence to support these claims is presented. Importantly, in Fig2 input controls for western blots are necessary to support the claim that levels of GSNOR1 are lower in aer mutant compared to WT or stable in GSNOR1 overexpressing line among treatments.

Based on enzymatic activity measurements, the authors claim that Ln-NO regulates GSNOR1. Suppression of GSNOR1 activity, although significantly different, is marginal compared to increases in SNO levels. Regarding this, it would be informative to compare increases in SNO levels induced by Ln-NO treatment in WT, gsnor1 mutant to investigate the relevance of SNO-GSNOR1 formation induced by Ln-NO. Based on data presented in Fig2C-D, it might be argued that GSNOR1 does not compensate for the increase in SNO levels induced by Ln-NO treatment, but it does very little to support the claim that it mediates accumulation of SNO.

It is unclear to me how treatment in Fig3A was performed. Were samples desalted after exposure to Ln-NO and cPTIO? The fact that cPTIO abolishes Ln-NO inhibitory effect on GSNOR1 activity suggests that NO is released to the medium/solution before it S-nitrosylates GSNOR1 (opposing to transnytroliation). If correct, this data appears to show that SNO-GSNOR1 formation is not directly mediated by Ln, thus contradicting the model presented by the authors (e.g. lines 262, 416...).

The authors present evidence that seed treatment with Ln-NO promotes seed germination. This is expected, as NO donors have been shown (including seminal work from the authors) to break seed dormancy. As presented, it is not clear how Ln-NO role in germination links to modulation of GSNOR1 activity. If GSNOR1-SNO formation represents the mechanism by which Ln-NO controls germination, above points on how to discriminate direct and indirect effects of Ln-NO treatment should be addressed. Here, again, the use of gsnor1 mutants and GSNOR1 overexpressor lines could prove handy.

Some more details on the rationale for the proteomics analyses could be provided. For instance, are the entities subjected to GO analysis the differentially S-nitrosylated proteins when mock, Ln and Ln-NO are compared (treatments mentioned in methods, line 679) ? If so, representation of the logical relationships between the data sets would be informative and should be presented. As the Biotin Switch Assay was used to label SNOs, minus ascorbate control could have been used to ensure specific labelling. A more detailed data mining with published SNO proteomics could also shed light on the unique roles of Ln-NO as a NO donor.

In light of the above comments, I would have expected a discussion on why GSNOR1 was not identified in the proteomics analysis.

Reviewer #2 (Comments for the Author):

This study by Mata-Pérez et al. seeks to elucidate the mechanistic link between Ln-NO2, global control of protein-SNO and their roles in seed germination.
While there is some evidence to support their proposed model that Ln-NO2 modulates protein-SNO through NO-dependent (NO release) modification of GSNOR1, the study appeared preliminary. This study also showed that Ln-NO2 increases seed germination but the proposed mechanistic link between seed germination and Ln-NO2 -mediated protein nitrosylation is weak. Additionally, the study may want to address the roles of (NO-independent) protein nitro-alkylation, which may be significant at the high Ln-NO2 concentrations used in this study.

Major Revisions/Comments

1) To demonstrate global modulation of protein-SNO by Ln-NO2, the authors performed proteomics and identified 593 proteins that are nitrosylated with Ln-NO2 treatment. Nonetheless, the proteomics part of the study is quite preliminary.
a. Key experimental details are left out and should be included.
i. How many biological repeats were performed?
ii. What were the filter criteria (or cutoffs) used?
iii. How were the proteins whose S-nitrosylation reduced by Ln-NO2 treatment (e.g. NDAP-ME2, RBCL), picked out by the proteomics? This seems counterintuitive.
b. The authors should strongly consider comparing their list of proteins in Table 1 with published s-nitrosylation proteomes, especially from studies looking at known nitrosylation targets (e.g. WT vs GSNOR1 knockout) or GSNO-induced s-nitrosylated proteins. This will help increase confidence in their data set and their conclusion that Ln-NO2 modulates global protein nitrosylation through GSNOR1.
c. Based on their earlier data, we would expect GSNOR1 and ABI5 to be identified in this proteomics experiment. Are they?
d. For the validation experiments by Western blot, key controls such as input samples (to ensure total protein levels are unaffected by the different treatments) and LA treatment (specificity) were not included. The authors should also include PTIO (NO-dependence) and GSNO in these Western blot validation experiments.
e. For "validated proteins", it should be worthwhile to also determine their nitrosylation states in the aer mutant (where there is higher protein-SNO and Ln-NO2),as well as in gnsor1 and GSNOR1 overexpressing mutants (to pick out gnsor1-dependent nitrosylation).

2) Since the role of GSNOR1 in global protein nitrosylation has previously been published, the novelty of this paper lies in proposal that Ln-NO2 inhibits GSNOR1 through an NO-dependent S-nitrosylation (Figure 3) but the data appears preliminary.
a. Figure 3 will greatly benefit from the inclusion of key controls to support the above conclusion.
i. Figure 3a, recombinant GSNOR1, lacking Ln, and GSNO, GSNO+PTIO, PTIO - specificity controls. Head-to-head comparison of GSNO vs Ln-NO2 may provide insights into S-nitrosylation vs nitro-alkylation.
ii. Figure 3b, recombinant GSNOR1, lacking Ln-NO2 + PTIO, GSNO, GSNO+PTIO- PTIO is needed to show NO-dependence. Also lacks protein load control.
iii. Figure 3c: The slight increase in GSNOR1-SNO for the aer mutant appears subjective. Also, based on figure 2a, there was an overall decrease in total GSNOR1 levels, which is not the case in Figure 3c.
b. The authors propose the need for high (100 μM) Ln-NO2 concentrations due to complex plant cell walls that limit lipid access. However, based on in vitro assay in figure 3a, the IC50 for GSNOR1 also appears to be in the high micromolar range. This may not correspond to the Ln-NO2 concentrations in vivo. The authors may want to address this.
c. The authors should consider the roles of protein nitro-alkylation (NO-independent) in their observations, which may or may not be the dominant modification of GSNOR1 compared to nitrosylation. Is it possible to perform intact MS or MS/MS on recombinant GSNOR1 or immunopurified FLAG-tagged GSNOR1 to identify modifications after Ln-NO2 and GSNO treatments?

3) Figure 4a showed that both Ln-NO2 and NO are important in modulating seed germination but it is insufficient to show Ln-NO2 modulates seed germination through NO release. This puts additional importance on Figure 4d, which lacks PTIO controls to show that the "increased BST signal" on ABI5 seen with Ln-NO2 is NO-dependent. Additionally, given the availability of recombinant protein, the authors may want to consider the possibility of protein nitro-alkylation (NO-independent) by mass spectrometry.

4) References to figures need to be checked, some examples below.
i. Page 9, line 246 - figure 1b seems to be wrongly cited
ii. Page 9, line 257 - figure 4d seems to be wrongly cited
iii. Page 11, Line 318: No Figure S4b

5) Methods section and figures can be improved in general, some examples below.
i. For example, nitro-linolenic synthesis (in methods), "with minor modifications" was not elaborated.
ii. Page 20, line 628, "GSNOR1 enzymatic assay" offered absolutely no details with the exception of two citations and "measured spectrophotometrically". It will greatly help readers if more details can be offered on how the assay was carried out.
iii. Figure 1a, what does the controls mean? It's challenging to interpret the data when the assays and reagents/workflow used is not even described in the paper. What does the scale bar mean? Method details on the confocal microscopy and image processing are absent.
iv. Figure 4d, what is asc?

Minor Revisions/Comments

6) Minor comments
a. Page 6, line 175: please change the word "underlined"
b. Page 11, line 347, main residues for nitro-alkylation are cysteines and histidines and to a smaller extent lysines
c. Page 14: line 433: Please edit "We helped us..."

Thank you for giving us the opportunity to review your manuscript for publication in the Plant Physiology. The reviewers have found the manuscript of interest but have raised a number of concerns that will require your attention. As these concerns are of some gravity and will entail substantial changes to the manuscript, you are likely to need additional time to address them. I must therefore reject the manuscript in its present form. Nonetheless, if you are able to address the points raised in review, I would welcome your resubmission of a manuscript that incorporates your responses.

Both reviewers and the editor found that the manuscript requires attention regarding structure, detailing the methods, and data interpretation. In particular, **I direct your attention to the following comments** for consideration in a resubmitted manuscript:

1. Given that Ln-NO functions as NO donor, if Ln-NO S-nitrosylates GSNOR1, provide explanation about how Ln-NO activities (donor vs trans-nitrosylating agent) are regulated, as raised by reviewer #1.

Comment:

See answer to comment 2 reviewer 1

2. Detail the methods, particularly regarding: (i) proteomics experiments (i.e. biological replicates, threshold used), (ii) approaches to identify proteins whose S-nitrosylation reduced by Ln-NO2 treatment, and (iii) effectiveness of thiol blocking, as indicated in the comments from reviewers #1 and #2.

Comment:

See answer to the comment 8 reviewer 1 and comments 1a, 1d, 1e of reviewer 2.

3. Provide the necessary controls for western blots to support the claims, as detailed in the comments of both reviewers.

Comment:

See the response to the comment 4 of reviewer 1 and comment 1d of reviewer 2.

4. Compare increases in SNO levels induced by Ln-NO treatment in WT, gsnor1 mutant to investigate the relevance of SNO-GSNOR1 formation induced by Ln-NO.

Comment:

See answer to the comment 5 of reviewer 1.

5. Comparing the list of proteins in Table 1 with published s-nitrosylation proteomes, especially from studies looking at known nitrosylation targets, as indicated in the comments of reviewer #2.

Comment:

According to the suggestion of both reviewers (comment 8 reviewer 1 and comment 1b of reviewer 2), we have built a new Table (**Supplemental Table 1**).

6. For proteins considered in the validation experiments, determine their nitrosylation states in the aer mutant as well as in gnsor1 and GSNOR1 overexpressing mutants, as indicated in the comments of reviewer #2

Comment:

See the response to the comment 1e of reviewer 2 (**Figure 5d** has changed accordingly)

7. Improve and streamline the Methods section and figures, as mentioned in the comments by both reviewers.

Comment:

We have highlighted in red colour all the modifications along the manuscript according to the suggestions of both reviewers.

Please note that any resubmission will be treated as a new entry to the system and is likely to go out for review.

If you do have any queries or concerns, you are welcome to contact me in the first instance.

Thank you again for allowing Plant Physiology to review your work. We look forward to hearing from you soon.

Reviewer #1 (Comments for the Author):

The manuscript by Perez et al is aimed at uncovering mechanisms of *S*-nitrosylation, a protein post-translational modification mediated by the redox active molecule nitric oxide (NO). Previous reports have established nitro-linoleic acid (Ln-NO_2) as an endogenous nitric oxide (NO) donor. However, little is known about the targets of Ln-NO_2 and its biological function. Here, the authors add to a body of evidence indicating that Ln-NO_2 functions as a NO donor in cells and controls seed germination. Based on protein and proteomics analyses, it is claimed that Ln-NO_2 targets NO turnover to affect stability of ABI5, a master regulator of seed germination.

The manuscript attempts to widen the understanding of SNO signalling in plants, particularly of nitro-fatty acids. In general, the manuscript requires some attention on structure and data interpretation - as described in more details below in the point-by-point assessment. Due to the experimental strategy used, it is difficult at times to establish causal links between Ln-NO_2, seed germination and the mechanism proposed.

Specific comments:

1. Line 55-57: the manuscript aims to address how Ln-NO_2 regulates GSNO bioavailability and SNO formation, and not GSNO biosynthesis as stated. The text should be corrected to reflect that.

Comment:

We apologised for the misunderstanding about the comment raised by the reviewer 1. We have previously addressed that NO_2-Ln is able to modulate GSNO biosynthesis in **Mata-Pérez *et al*., 2020**. In this first paragraph of the summary, we just frame some of the previous results about NO_2-FAs such as the capacity of NO_2-Ln to modulate the GSNO formation and how this might be pointing to a modulation of the SNO metabolism through NO_2-FAs.

2. Line 65-66: given that Ln-NO_2 functions as NO donor, if Ln-NO_2 S-nitrosylates GSNOR1 (see comments below on evidence for this claim) how are Ln-NO_2 activities (donor vs trans-nitrosylating agent) regulated? In addition, the authors seem to fluctuate between these two non-mutually exclusive scenarios throughout the manuscript. A careful revision of the text would help clarify the message.

Comment:

We apologize about this misunderstanding. The basis of this manuscript relies on the already proven capacity of NO_2-FAs to release NO in animal and plant systems (see references cited throughout the text). On this basis, we show throughout this manuscript that NO coming from NO_2-Ln can mediate the *S*-nitrosylation of a set of proteins among which we found already described SNO-targets but also other novel targets of this post-translational modification **(see Table 1 and Supplemental Table 1)**.

As stated for other NO donors such as GSNO, we cannot rule out that NO_2-Ln could also act as a trans-nitrosylating agent. Indeed, as described in Mata-Pérez *et al*., 2016, both NO_2-Ln and GSNO have similar behaviours in releasing NO to the media **(see Table 1, Mata-Pérez *et al*., 2016)**. However, what we mostly observe throughout the experiments is an opposite behaviour when adding NO_2-Ln *vs* cPTIO (a NO scavenger). This is the

reason why we assume that the results observed with NO_2-Ln are mostly due to its capacity to release NO.

3. Throughout, details of the methodological approach are missing. This hinders data analysis and interpretation. For instance, how cells were treated in Fig1A is unclear. Were cells exposed to DTPA and NEM before addition of or along with Ln-NO_2? Thiol alkylation by NEM is often performed at 50C, so it is unclear how effective thiol blocking was in the assay. I am also unsure about the faint signal in the '-fluorophore' sample. If 'fluorophore' refers to the fluorescent probe DAF used in the assay, what is the difference between Fig1C and E?

Comment:

We apologize for not give more details about the SNO detection by confocal microscopy. We just detailed the reference of **Chaki *et al.*, 2011b** because we followed the same approach described in that manuscript.

First, we would like to highlight that **the fluorophore** used for -SNO detection by CLSM is **Alexa Fluor 288 Hg-link phenylmercury** which can be used for direct *S*-nitrosothiol detection in plant tissues when samples are preincubated with N-ethylmaleimide (NEM) to block free sulfhydryl groups. Under these conditions, the fluorescent Hg-Link reagent labels R-SNO- modified proteins and peptides according to the following equation:

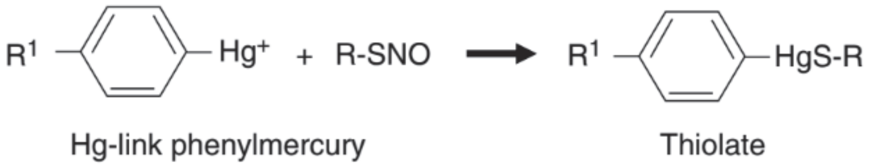

With more detail and in all the treatments, cell cultures were incubated with methanol (vehicle, Fig. 1A), 100 µM linolenic acid (Fig. 1B) or 100 µM NO_2-Ln for 1h (Figs. 1C-1E). In Figs 1A-1C, samples were incubated with 100 µM DTPA and 10 mM NEM for 2h at 25ºC to block free sulfhydryl groups. Regarding the temperature of blocking, it has been shown in many studies that 25ºC is a right temperature for blocking -SH groups **(Chaki et al., 2011b; Valderrama et al., 2007; Airaki et al., 2011) and the method *per se* has been already published in "Plant Nitric Oxide: Methods and Protocols" (2016)**. After DTPA and NEM incubation, samples were washed three times with 10 mM PBS for 15 min each. Afterwards, these samples were incubated with 10 µM Alexa fluor 488 Hg-link phenylmercury (Molecular Probes, Eugene, OR, USA, cat.no H30462) for 1 h at 25°C, in darkness and washed three times in PBS for 15 min each.

Regarding the controls shown in Figs 1D-E, after cell cultures were incubated with 100 µM NO_2-Ln and washed three times with 10 mM PBS, one sample was incubated with DTPA and NEM as described above (Fig. 1D), followed by three washes with 10 mM PBS, but the Alexa fluor 488 Hg-link phenylmercury was not added to the sample as a negative control. In a similar way, in Fig. 1E, samples were not prior incubated with DTPA and NEM and, after the following washes with PBS, the fluorophore was added to the sample and used as a negative control too.

In this regard, we have included a wider explanation about the protocol of SNO detection by CLSM in the material and methods section (lines **720-734**).

4. The authors claim that GSNOR1 is repressed at transcriptional, protein and activity levels by Ln-NO_2. Although some genetic evidence is provided by using

aer mutant plants, no direct evidence to support these claims is presented. Importantly, in Fig2 input controls for western blots are necessary to support the claim that levels of GSNOR1 are lower in aer mutant compared to WT or stable in GSNOR1 overexpressing line among treatments.

Comment:

Regarding this comment, we do not understand exactly what the reviewer is asking for. In Figure 2, we show that GSNOR1 transcript levels are diminished in the *aer* mutant (Fig. 2A) as well both protein and enzymatic activities (Fig. 2B). Furthermore, in Fig. 3A, we incubated recombinant GSNOR1 with NO_2-Ln and we observed a similar trend in the GSNOR1 bioactivity. Together, these results reinforce the role of NO_2-Ln as a modulator of GSNOR1 enzymatic activity.

Regarding the input controls, we apologise for the missing data, and we have included them in Figs. 2B and 2D. Furthermore, we have added in the corresponding figure legends indications about the controls showed.

5. Based on enzymatic activity measurements, the authors claim that Ln-NO_2 regulates GSNOR1. Suppression of GSNOR1 activity, although significantly different, is marginal compared to increases in SNO levels. Regarding this, it would be informative to compare increases in SNO levels induced by Ln-NO_2 treatment in WT, *gsnor1* mutant to investigate the relevance of SNO-GSNOR1 formation induced by Ln-NO_2. Based on data presented in Fig2C-D, it might be argued that GSNOR1 does not compensate for the increase in SNO levels induced by Ln-NO_2 treatment, but it does very little to support the claim that it mediates accumulation of SNO.

Comment:

We agree with the reviewer this is a good appreciation. However, we used different techniques: ozone chemiluminescence to determine SNOs and a spectrophotometric approach to determine GSNOR activity by evaluating the NADH consumption along time. In this regard, we agree that what we observe are tendencies: a significant decrease in the GSNOR1 activity after NO_2-Ln application correlates with a significant increase in the SNO levels. In fact, a decrease in GSNOR1 activity leads to an increase in the GSNO content, however with the ozone chemiluminescence approach what we mostly quantify are the high molecular weight SNOs, namely, protein-SNO. Even thought we are not directly quantifying GSNO *per se*, we are able to observe a significant increase in the total SNO levels.

6. It is unclear to me how treatment in Fig3A was performed. Were samples desalted after exposure to Ln-NO_2 and cPTIO? The fact that cPTIO abolishes Ln-NO_2 inhibitory effect on GSNOR1 activity suggests that NO is released to the medium/solution before it *S*-nitrosylates GSNOR1 (opposing to transnitrosylation). If correct, this data appears to show that SNO-GSNOR1 formation is not directly mediated by Ln, thus contradicting the model presented by the authors (e.g. lines 262, 416...).

Comment:

We apologize for any misunderstanding about the cPTIO treatment on the GSNOR1 recombinant protein. We have included additional information in this regard into the material and methods section (lines **700-706**). Specifically, cPTIO treatment was

concomitant to the NO_2-Ln application with the aim of scavenging the NO that comes from the nitro-fatty acid just when added into the solution. We carried out the approaches in the same way for all the treatments. Results show that NO removal from the assay (cPTIO treatment) and -SNO break (with DTT) leads to the recovery of GSNOR1 activity suggesting that the capacity of NO_2-Ln to release NO in an aqueous solution leads to the inhibition of GSNOR1 activity.

When working with NO, the cPTIO treatment is a classical approach to attribute a certain behaviour to NO. With this approach, we showed that NO coming from NO_2-Ln is released into the solution and scavenged by cPTIO thus leading to the recovery of GSNOR1 activity.

Also, as suggested by reviewer 2, we have repeated this assay including the controls we have been asked for as shown in **Figure 3A**.

7. The authors present evidence that seeds treatment with Ln-NO_2 promotes seed germination. This is expected, as NO donors have been shown (including seminal work from the authors) to break seed dormancy. As presented, it is not clear how Ln-NO_2 role in germination links to modulation of GSNOR1 activity. If GSNOR1-SNO formation represents the mechanism by which Ln-NO_2 controls germination, above points on how to discriminate direct and indirect effects of Ln-NO_2 treatment should be addressed. Here, again, the use of gsnor1 mutants and GSNOR1 overexpressor lines could prove handy.

Comment:

This is a good appreciation. However, we are not attributing to NO_2-Ln the capacity to modulate the germination onset through the *S*-nitrosylation of GSNOR1 but focusing on the already proven capacity of NO_2-Ln to release NO and to modulate specific protein targets involved in plant germination such as the ABI5 transcription factor.

By using Col-0 seeds treated with NO_2-Ln and the *aer* mutant (high NO_2-Ln levels), we are focusing the attention on the NO_2-Ln and its capacity to modulate the SNO metabolism as stated above. As we discuss in the manuscript, the role of NO_2-Ln in seed dormancy break might be related to the contribution of the NO coming from this nitro-fatty acid to the pool of NO needed to break dormancy but we cannot rule out that other mechanisms might be activated upon NO_2-Ln addition.

Furthermore, this NO within the plant cell can lead to the *S*-nitrosylation of a set of proteins including ABI5, a central regulator of seed germination (**Albertos *et al.*, 2015**). As shown in Figure 4c, ABI5 levels are lower in the *aer* mutant suggesting a role of NO_2-Ln in ABI5 accumulation. Due to the technical difficulties associated to study the *S*-nitrosylation of ABI5 *in vivo*, the *in vitro* biotin-switch technique shows that NO_2-Ln can *S*-nitrosylate this transcription factor (and that this behaviour is reversed when cPTIO is added) pointing to a role for this nitro-fatty acid in modulating the stability of ABI5 through a *S*-nitrosylation mechanism.

8. Some more details on the rationale for the proteomics analyses could be provided. For instance, are the entities subjected to GO analysis the differentially S-nitrosylated proteins when mock, Ln and Ln-NO are compared (treatments mentioned in methods, line 679)? If so, representation of the logical relationships between the data sets would be informative and should be presented. As the Biotin Switch Assay was used to label SNOs, minus ascorbate control could have been used to ensure specific labelling. A more detailed data mining with published

SNO proteomics could also shed light on the unique roles of Ln-NO as a NO donor.

Comment:

The GO terms depicted in Figure 5 correspond to the set of proteins *S*-nitrosylated by NO_2-Ln after comparing to the controls with methanol (vehicle) and Ln. As performed in other published proteomic approaches focused on studying protein-SNO after the biotin switch technique (**Lindermayr *et al*., 2005; Romero-Puertas *et al*., 2008; Fares *et al*., 2011; Kato *et al*., 2012; Niu *et al*., 2019**), only biotinylated proteins are detected through mass spectrometry. Therefore, the best controls of the mass spectrometry approach are the vehicle (MeOH)- and Ln-treatments to discard off-targets of NO_2-Ln.

As it can be shown in Figures 1b, 1d or 3c, the minus ascorbate o DTT-treated samples during the biotin switch technique work perfectly as negative controls of the technique.

According to the reviewer suggestion, we have built a table comparing the available *S*-nitrosylomes employing other NO donors (GSNO, NO gas…) and NO_2-Ln, which reinforces the *S*-nitrosylating capacity of this NO_2-FA. This can be found in **Supplemental Table 1**. Text has been included in lines **421-429** and **550-561**.

9. In light of the above comments, I would have expected a discussion on why GSNOR1 was not identified in the proteomics analysis.

Comment:

We agree with the reviewer this is a very good appreciation. For the proteomics analysis, we employed Arabidopsis cell suspension cultures and, we selected this system because we could apply in liquid media the molecule of interest in this study, the NO_2-Ln. We believe GSNOR1 is in a very low abundance in cell cultures due to two main reasons:

- It has been already described that *S*-nitrosylation of GSNOR1 could lead to its degradation by selective autophagy (**Zhan *et al*., 2018**). We cannot rule out in which extent the NO_2-Ln might be interfering in GSNOR1 stability. Indeed, GSNOR1 has not been previously detected in other protein-SNO studies (**Lindermayr *et al*., 2005; Romero-Puertas *et al*., 2008; Fares *et al*., 2011; Kato *et al*., 2012**) even though it has been described as a target of *S*-nitrosylation (**Frungillo *et al*., 2014**).
- GSNOR1 protein has been described as an important player during stress responses highlighting plant immunity (**Feechan *et al*., 2005; Kneeshaw *et al*., 2014**), the response to mechanical wounding (**Chaki *et al*., 2011a**) or to salinity (**Valderrama *et al*., 2007; Begara-Morales *et al*., 2014**), among others. Given the application of NO_2-Ln does not trigger a stressful situation (as described in **Mata-Pérez *et al*., 2016**), this might be the reason why we are not able to find GSNOR1-SNO among the identified SNO-proteins in our proteomic study.

We have included some text related to this aspect in the discussion section (lines **577-596**).

Reviewer #2 (Comments for the Author):

This study by Mata-Pérez et al. seeks to elucidate the mechanistic link between Ln-NO2, global control of protein-SNO and their roles in seed germination. While there is some evidence to support their proposed model that Ln-NO2 modulates protein-SNO through NO-dependent (NO release) modification of GSNOR1, the study appeared preliminary. This study also showed that Ln-NO2 increases seed germination but the proposed mechanistic link between seed germination and Ln-NO2 -mediated protein nitrosylation is weak. Additionally, the study may want to address the roles of (NO-independent) protein nitro-alkylation, which may be significant at the high Ln-NO2 concentrations used in this study.

Major Revisions/Comments:

1. To demonstrate global modulation of protein-SNO by Ln-NO_2, the authors performed proteomics and identified 593 proteins that are nitrosylated with Ln-NO_2 treatment. Nonetheless, the proteomics part of the study is quite preliminary.

a. Key experimental details are left out and should be included.

i. How many biological repeats were performed?

Comment:

We used three biological replicates from each condition. Text has been included in lines **768-770**.

ii. What were the filter criteria (or cutoffs) used?

Comment:

The parameters used to stablish a threshold have been detailed in the Material and Methods section (lines **806-810**).

Specifically, a first comparison between the protein-SNO detected in methanol and Ln-treated cell suspension cultures was performed to discard proteins that might be *S*-nitrosylated by the fatty acid alone (Ln). This list of protein-SNO was after compared to NO_2-Ln-treated cell cultures to know the identity of these *S*-nitrosylated protein targets. In this sense, peptides identified as unique (peptide sequences that can only belong to one protein where at least two peptides were detected) were considered for the mass spectrometric analysis resulting in 1520 unique peptide spectra defining 593 proteins.

iii. How were the proteins whose S-nitrosylation reduced by Ln-NO2 treatment (e.g. NDAP-ME2, RBCL), picked out by the proteomics? This seems counterintuitive.

Comment:

The reviewer is totally right, and this might seem counterintuitive. For this reason, we have made a new figure checking the *S*-nitrosylation pattern of some of the targets identified in the proteomics and for which there is an available antibody. The reviewer can check it on Figure 5d. Moreover, we have included some other controls to get a solid response attributed to NO_2-Ln.

b. The authors should strongly consider comparing their list of proteins in Table 1 with published s-nitrosylation proteomes, especially from studies looking at known nitrosylation targets (e.g. WT vs GSNOR1 knockout) or GSNO-induced s-

nitrosylated proteins. This will help increase confidence in their data set and their conclusion that Ln-NO_2 modulates global protein nitrosylation through GSNOR1.

Comment:

We totally agree that a comparison with other *S*-nitrosylomes could be of great interest here. According to the suggestions of both reviewers, we have built a table with the available information on protein-SNO in plants after the application of different NO donors (**Supplemental Table 1**). Text has been included in lines **421-429** and **550-561**.

c. Based on their earlier data, we would expect GSNOR1 and ABI5 to be identified in this proteomics experiment. Are they?

Comment:

We agree with the reviewer this is a very good appreciation. For the proteomics analysis, we employed Arabidopsis cell suspension cultures and, we selected this system because we could apply in liquid media the molecule of interest in this study, the NO_2-Ln.

We believe GSNOR1 is in a very low abundance in cell cultures due to two main reasons:

- It has been already described that *S*-nitrosylation of GSNOR1 could lead to its degradation by selective autophagy (**Zhan *et al*., 2018**). We cannot rule out in which extent the NO_2-Ln might be interfering in GSNOR1 stability. Indeed, GSNOR1 has not been previously detected in other protein-SNO studies (**Lindermayr *et al*., 2005; Romero-Puertas *et al*., 2008; Fares *et al*., 2011; Kato *et al*., 2012**) even though it has been described as a target of *S*-nitrosylation (**Frungillo *et al*., 2014**).
- GSNOR1 protein has been described as an important player during stress responses highlighting plant immunity (**Feechan *et al*. 2005; Kneeshaw *et al*., 2014**), the response to mechanical wounding (**Chaki *et al*., 2011a**) or to salinity (**Valderrama *et al*., 2007; Begara-Morales *et al*., 2014**), among others. Given the application of NO_2-Ln does not represent a stressful situation (as described in **Mata-Pérez *et al*., 2016**), this might be the reason why we do not find GSNOR1-SNO among the identified SNO-proteins in our proteomic study.

Regarding why ABI5 is not detected among the *S*-nitrosylated proteins identified in our study, ABI5 is a transcription factor crucial for seed break dormancy with a high expression at early stages of plant development. We think ABI5 is not present among the *S*-nitrosylated proteins detected by our proteomics study because we used cell suspension cultures where ABI5 is, essentially, not needed. Furthermore, as already described, *S*-nitrosylation of ABI5 leads to its degradation through the proteosome pathway (**Albertos *et al*., 2015**) so we cannot rule out that a combination of both factors is responsible for not detecting ABI5-SNO in this study.

We have included some text related to this aspect in the discussion section (lines **577-590**.

d. For the validation experiments by Western blot, key controls such as input samples (to ensure total protein levels are unaffected by the different treatments) and LA treatment (specificity) were not included. The authors should also include PTIO (NO-dependence) and GSNO in these Western blot validation experiments.

Comment:

In relation to the comment previously raised by the reviewer 2 (comment 1a-iii) we have included the loading controls to ensure the validity of the data. This can be found in Figure 5d. Figure legend has been modified accordingly.

e. For "validated proteins", it should be worthwhile to also determine their nitrosylation states in the aer mutant (where there is higher protein-SNO and Ln-NO_2), as well as in gnsor1 and GSNOR1 overexpressing mutants (to pick out gnsor1-dependent nitrosylation).

Comment:

In relation to the comments previously raised by the reviewer 2 (comments 1a-iii and 1d) we have included the *aer* and the *gsnor1* mutants to ensure the validity of the data. This can be found in Figure 5d. Figure legend has changed accordingly and text related has been included in lines **425-429**.

2. Since the role of GSNOR1 in global protein nitrosylation has previously been published, the novelty of this paper lies in proposal that Ln-NO_2 inhibits GSNOR1 through an NO-dependent S-nitrosylation (Figure 3) but the data appears preliminary.

a. Figure 3 will greatly benefit from the inclusion of key controls to support the above conclusion.

i. Figure 3a, recombinant GSNOR1, lacking Ln, and GSNO, GSNO+PTIO, PTIO - specificity controls. Head-to-head comparison of GSNO vs Ln-NO_2 may provide insights into S-nitrosylation vs nitro-alkylation.

Comment:

We agree with the reviewer that some of the controls were missing in this figure. It is remarkable to note that this experiment has been performed before in the work of **Guerra *et al*., 2016** where it is shown how GSNO leads to a decrease of the GSNOR activity (Figure 1). As observed in that paper, GSNOR activity is rescued by DTT treatment. Also, **Frungillo** and colleagues **(2014)** showed how Cys-NO and DEA-NO treatments lead to a fall in the GSNOR activity by these NO donors.

Following the recommendation of the reviewer 2, we have repeated the experiment including a bunch of different conditions as shown in **Figure 3a**. We have included control conditions such as the untreated, vehicle and Ln conditions for the NO_2-Ln treatment together with the combined treatments of NO_2-Ln and cPTIO or DTT. As it can be seen in the figure, both GSNO and SNAP treatments have been included alone or with the combination with DTT or cPTIO, respectively. Figure legend has been modified accordingly and we have included the experimental information in the Methods section.

This set of results shows that GSNOR activity restores to control conditions when incubated with cPTIO or with DTT, suggesting a NO-dependent mechanism instead of a nitroalkylation process. Explanation can be found in lines **264-275**.

ii. Figure 3b, recombinant GSNOR1, lacking Ln-NO_2 + PTIO, GSNO, GSNO+PTIO- PTIO is needed to show NO-dependence. Also lacks protein load control.

Comment:

This is a very good appreciation, and we coincide with the reviewer some of the controls of the experiment were missing. In this regard, **Figure 3b** has been changed according to the comments raised by the reviewer 2.

We did not include in the assay the condition where GSNO can be used as a NO donor due to GSNO is the substrate of GSNOR1.

Figure legend has been modified accordingly. Text has been included in lines **271-275**.

iii. Figure 3c: The slight increase in GSNOR1-SNO for the aer mutant appears subjective. Also, based on figure 2a, there was an overall decrease in total GSNOR1 levels, which is not the case in Figure 3c.

Comment:

We agree with this appreciation. We have tried to check the *in vivo S*-nitrosylation of GSNOR1 in the *aer* mutant, but we have failed few times in detecting this post-translational modification both at the beginning of plant development and in rosette leaves (where GSNOR1 expression is higher). We think both the low abundance of GSNOR1-SNO, and the putative NO-dependent degradation (**Zhan *et al.*, 2018**), makes very difficult to assess the endogenous GSNOR1-SNO.

However, as the reviewer 2 can see in the next picture, the levels of GSNOR1 in the *aer* mutant are lower compared to Col-0 and this might be the reason why we do not detect GSNOR1-SNO in the *aer* mutant. The picture shows a representative immunoblot of a biotin switch experiment (Unt: untreated; -Asc: negative control where the -SH is not reduced by ascorbate prior the addition of Biotin-HPDP; +DTT: SNO break induced by DTT addition).

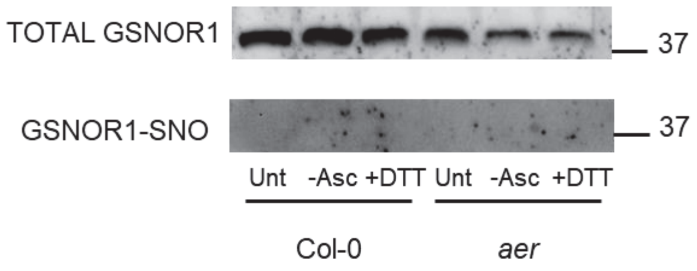

b. The authors propose the need for high (100 μM) Ln-NO_2 concentrations due to complex plant cell walls that limit lipid access. However, based on in vitro assay in figure 3a, the IC50 for GSNOR1 also appears to be in the high micromolar range. This may not correspond to the Ln-NO_2 concentrations in vivo. The authors may want to address this.

Comment:

We agree with the reviewer, and we consider this is a good appreciation.

Previous studies about the presence and concentration of nitro-fatty acids (NO_2-FAs) both in animal and plant systems have shown these molecules stand in the cells at low concentrations, ranging from picomolar to nanomolar in animals (**Tsikas *et al.*, 2009**)

and plants (**Mata-Pérez *et al.*, 2016; Aranda-Caño *et al.*, 2022**). However, most of the studies performed in this field have used higher concentrations of these nitrated derivatives to evidence the physiological implications in biological systems (**Kansanen *et al.*, 2009; Mata-Pérez *et al.*, 2016**). In this sense and when we initiated the study about the role of these signalling molecules in plants, we assayed several concentrations of NO_2-Ln and we observed a dose-dependence response (see below the supplemental figure 6 extracted from (**Mata-Pérez *et al.*, 2016**)). However, we selected 100 µM of NO_2-Ln as standard concentration for all the assays to evidence a clear and solid response due to this NO_2-FA.

We agree the concentration employed for the *in vitro* assays is higher than the endogenously present within the cells. However, we selected the 100 µM concentration as a standard for all the approaches performed along the manuscript.

We have included an explanation about the concentration used throughout the manuscript in the lines **207-216**.

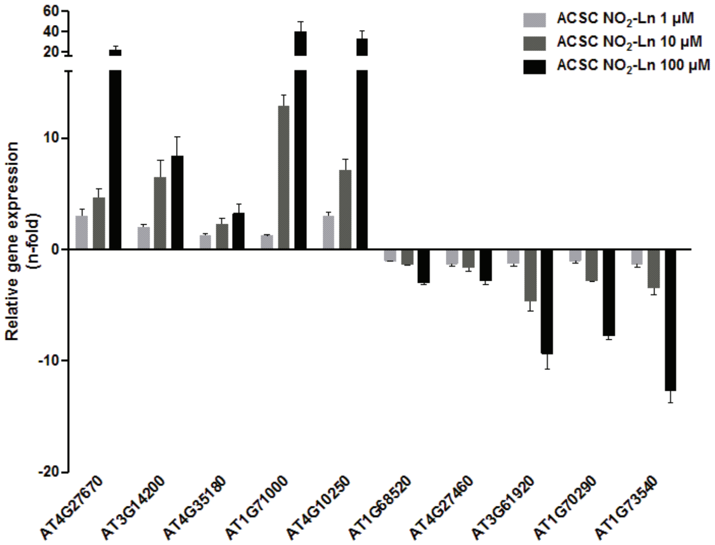

Figure S6. Dose-dependence gene response of NO_2-Ln treatment in ACSC by qRT-PCR. Ten genes previously identified as NO_2-Ln-responsive genes by RNA-seq in ACSC were randomly selected to analyse, by qRT–PCR, the differential expression changes at 1, 10 and 100 µM NO_2-Ln. Results are average data of two independent experiments in triplicate with standard deviation. HPS21 (AT4G27670) *A. thaliana* heat shock protein 21; HSP40 (AT3G14200) *A. thaliana* chaperone DnaJ-domain superfamily protein; LHT7 (AT4G35180) *A. thaliana* lys/his transporter 7; AT1G71000 *A. thaliana* chaperone DnaJ-domain superfamily protein; HSP22.0 (AT4G10250) *A. thaliana* columbia endomembrane-localized small heat shock protein; BBX14 (AT1G68520) *A. thaliana* B-box type zinc finger protein with CCT domain; CBSX5 (AT4G27460) *A. thaliana* cystathionine beta-synthase (CBS) family protein; AT3G61920 *A. thaliana* unknown protein; TPS8 (AT1G70290) *A. thaliana* trehalose-6-phosphatase synthase S8; NUDT21 (AT1G73540) *A. thaliana* nudix hydrolase homolog 21.

c. The authors should consider the roles of protein nitro-alkylation (NO-independent) in their observations, which may or may not be the dominant modification of GSNOR1 compared to nitrosylation. Is it possible to perform intact MS or MS/MS on recombinant GSNOR1 or immunopurified FLAG-tagged GSNOR1 to identify modifications after Ln-NO_2 and GSNO treatments?

Comment:

We coincide this is a good appreciation. Indeed, NO_2-Ln can mediate both nitroalkylation and NO donor activities. However, the body of evidence showed throughout the manuscript directs the bioactivity of NO_2-Ln towards a *S*-nitrosylation process -instead of nitroalkylation- in terms of the processes and targets we are assessing here such as seed germination and GSNOR1 or ABI5, respectively. We also know NO_2-Ln is capable of mediating protein nitroalkylation in other plant targets such as APX (**Aranda-Caño *et al.*, 2019**) so it is not our intention to show that this nitro-fatty acid only mediates protein *S*-nitrosylation. The solely intention in this work is to show that NO_2-FAs, through the capacity to release NO and to modify the protein-SNO content, can modulate relevant processes in plants such as seed germination.

3. Figure 4a showed that both Ln-NO_2 and NO are important in modulating seed germination, but it is insufficient to show Ln-NO_2 modulates seed germination through NO release. This puts additional importance on Figure 4d, which lacks PTIO controls to show that the "increased BST signal" on ABI5 seen with Ln-NO_2 is NO-dependent. Additionally, given the availability of recombinant protein, the authors may want to consider the possibility of protein nitro-alkylation (NO-independent) by mass spectrometry.

Comment:

The reviewer is right, and we think this is a good appreciation. We have repeated the approach carried out in Figure 4d but including two different concentrations of NO_2-Ln (50 and 100 µM), GSNO (500 µM and 1 mM) and the subsequent cPTIO controls. As shown, concomitant cPTIO incubation with both NO_2-Ln and GSNO leads to observe a faint signal in terms of ABI5-SNO. These results highlight that NO_2-Ln is mediating the *S*-nitrosylation of ABI5 in a NO-dependent manner. Nonetheless, we cannot rule out that nitroalkylation could be also playing a role in the regulation of this TF.

We did not show it before, but we carried out the BST with the mutated version of ABI5 (Cys153Ser) to check whether NO_2-Ln might mediate the *S*-nitrosylation of ABI5 in the same Cys residue previously reported to underly this NO-PTM with other NO donors such as GSNO (**Albertos *et al.*, 2015**). As shown in **Figure S2c**, addition of GSNO or NO_2-Ln to this Cys153Ser-ABI5 does not impact on ABI5-SNO suggesting that ABI5-SNO mainly relies on Cys153. Text has been included in lines **343-350**.

4. References to figures need to be checked, some examples below.

   i. Page 9, line 246 - figure 1b seems to be wrongly cited

Comment:

We apologize for the mistake. The text has been corrected accordingly.

   ii. Page 9, line 257 - figure 4d seems to be wrongly cited

Comment:

We apologize for the mistake. The text has been corrected accordingly.

   iii. Page 11, Line 318: No Figure S4b

Comment:

We apologize for the mistake. The text has been modified accordingly.

5. Methods section and figures can be improved in general, some examples below.

i. For example, nitro-linolenic synthesis (in methods), "with minor modifications" was not elaborated.

Comment:

We apologize for not explaining the methodology in more detail. We have included a brief explanation of the procedure in lines **639-650**.

ii. Page 20, line 628, "GSNOR1 enzymatic assay" offered absolutely no details with the exception of two citations and "measured spectrophotometrically". It will greatly help readers if more details can be offered on how the assay was carried out.

Comment:

We apologize for not explaining the methodology in more detail. We have included a brief explanation of the procedure in lines **700-706**.

iii. Figure 1a, what does the controls mean? It's challenging to interpret the data when the assays and reagents/workflow used is not even described in the paper. What does the scale bar mean? Method details on the confocal microscopy and image processing are absent.

Comment:

We apologize for not explaining the methodology in more detail. We have included a brief explanation of the procedure in lines **720-734**. Bars indicate 50 µm and this has been indicated in Figure 1d legend.

iv. Figure 4d, what is asc?

Comment:

We apologize for this missing detail. We have included in the figure legend, accordingly.

Minor Revisions/Comments:

6. Minor comments

a. Page 6, line 175: please change the word "underlined"

Comment:

We have changed the word accordingly.

b. Page 11, line 347, main residues for nitro-alkylation are cysteines and histidines and to a smaller extent lysines

Comment:

We have included Cys and Lys residues in the text.

c. Page 14: line 433: Please edit "We helped us..."

Comment:

We have changed the word according to the reviewer suggestion.

REFERENCES CITED WITHIN THE REVISION

Mata-Pérez C, Padilla MN, Sánchez-Calvo B, Begara-Morales JC, Valderrama R, Chaki M, Aranda-Caño L, Moreno-González D, Molina-Díaz A, Barroso JB. 2020. Endogenous biosynthesis of S-nitrosoglutathione from nitro-fatty acids in plants. *Frontiers in plant science*: 962.

Mata-Pérez C, Sánchez-Calvo B, Begara-Morales JC, Carreras A, Padilla MN, Melguizo M, Valderrama R, Corpas FJ, Barroso JB. 2016a. Nitro-linolenic acid is a nitric oxide donor. *Nitric Oxide* **57**: 57-63.

Chaki M, Valderrama R, Fernández-Ocaña AM, Carreras A, Gómez-Rodríguez MV, López-Jaramillo J, Begara-Morales JC, Sánchez-Calvo B, Luque F, Leterrier M. 2011b. High temperature triggers the metabolism of S-nitrosothiols in sunflower mediating a process of nitrosative stress which provokes the inhibition of ferredoxin–NADP reductase by tyrosine nitration. *Plant, Cell & Environment* **34**(11): 1803-1818.

Valderrama R, Corpas FJ, Carreras A, Fernández-Ocaña A, Chaki M, Luque F, Gómez-Rodríguez MV, Colmenero-Varea P, del Río LA, Barroso JB. 2007. Nitrosative stress in plants. *FEBS letters* **581**(3): 453-461.

Airaki M, Sánchez-Moreno L, Leterrier M, Barroso JB, Palma JM, Corpas FJ. 2011. Detection and quantification of S-nitrosoglutathione (GSNO) in pepper (Capsicum annuum L.) plant organs by LC-ES/MS. *Plant Cell Physiology* **52**(11): 2006-15.

Barroso JB, Valderrama R, Carreras A, Chaki M, Begara-Morales JC, Sánchez-Calvo B, Corpas FJ. 2016. Quantification and Localization of S-Nitrosothiols (SNOs) in Higher Plants. Plant Nitric Oxide: Methods and Protocols. **1424**:139-48.

Albertos P, Romero-Puertas MC, Tatematsu K, Mateos I, Sánchez-Vicente I, Nambara E, Lorenzo O. 2015. S-nitrosylation triggers ABI5 degradation to promote seed germination and seedling growth. *Nature communications* **6**.

Lindermayr C, Saalbach G, Durner J. 2005. Proteomic identification of S-nitrosylated proteins in Arabidopsis. *Plant Physiology* **137**(3): 921-930.

Romero-Puertas MC, Campostrini N, Mattè A, Righetti PG, Perazzolli M, Zolla L, Roepstorff P, Delledonne M. 2008. Proteomic analysis of S-nitrosylated proteins in Arabidopsis thaliana undergoing hypersensitive response. *Proteomics* **8**(7): 1459.

Fares A, Rossignol M, Peltier J-B. 2011. Proteomics investigation of endogenous S-nitrosylation in Arabidopsis. *Biochemical and biophysical research communications* **416**(3): 331-336.

Kato H, Takemoto D, Kawakita K. 2012. Proteomic analysis of *S*-nitrosylated proteins in potato plant. *Physiologia Plantarum* **148**(3):371-86.

Niu L, Yu J, Liao W, Xie J, Yu J, Lv J, Xiao X, Hu L, Wu Y. 2019. Proteomic investigation of *S*-nitrosylated proteins during NO-induced adventitious rooting of cucumber. *Int J Mol Sci* **20**(21): 5363

Palmieri MC, Lindermayr C, Bauwe H, Steinhauser C, Durner J. 2010. Regulation of plant glycine decarboxylase by *S*-nitrosylation and glutathionylation. *Plant Physiology* **152**(3):1514-28.

Zhan N, Wang C, Chen L, Yang H, Feng J, Gong X, Ren B, Wu R, Mu J, Li Y. 2018. S-nitrosylation targets GSNO reductase for selective autophagy during hypoxia responses in plants. *Molecular cell* **71**(1): 142-154. e146.

Frungillo L, Skelly MJ, Loake GJ, Spoel SH, Salgado I. 2014. S-nitrosothiols regulate nitric oxide production and storage in plants through the nitrogen assimilation pathway. *Nature communications* **5**.

Feechan A, Kwon E, Yun B-W, Wang Y, Pallas JA, Loake GJ. 2005. A central role for S-nitrosothiols in plant disease resistance. *Proceedings of the National Academy of Sciences* **102**(22): 8054-8059.

Kneeshaw S, Gelineau S, Tada Y, Loake GJ, Spoel SH. 2014. Selective protein denitrosylation activity of thioredoxin-h5 modulates plant immunity. *Molecular cell* **56**(1): 153-162.

Chaki M, Valderrama R, Fernández-Ocaña AM, Carreras A, Gómez-Rodríguez MV, Pedrajas JR, Begara-Morales JC, Sánchez-Calvo B, Luque F, Leterrier M. 2011a. Mechanical wounding induces a nitrosative stress by down-regulation of GSNO reductase and an increase in S-nitrosothiols in sunflower (Helianthus annuus) seedlings. *Journal of experimental botany* **62**(6): 1803-1813.

Begara-Morales JC, Sánchez-Calvo B, Chaki M, Valderrama R, Mata-Pérez C, López-Jaramillo J, Padilla MN, Carreras A, Corpas FJ, Barroso JB. 2014. Dual regulation of

cytosolic ascorbate peroxidase (APX) by tyrosine nitration and S-nitrosylation. *Journal of experimental botany*: ert396.

Guerra D, Ballard K, Truebridge I, Vierling E. 2016. S-nitrosation of conserved cysteines modulates activity and stability of S-nitrosoglutathione reductase (GSNOR). *Biochemistry* **55**(17): 2452-2464.

Tsikas D, Zoerner AA, Mitschke A, Gutzki F-M. 2009. Nitro-fatty acids occur in human plasma in the picomolar range: a targeted nitro-lipidomics GC–MS/MS study. Lipids 44(9): 855-865.

Aranda-Caño L, Valderrama R, Chaki M, Begara-Morales JC, Melguizo M, Barroso JB. 2022. Nitrated Fatty-Acids Distribution in Storage Biomolecules during Arabidopsis thaliana Development. *Antioxidants* **11**(10): 1869.

Kansanen E, Jyrkkänen H-K, Volger OL, Leinonen H, Kivelä AM, Häkkinen S-K, Woodcock SR, Schopfer FJ, Horrevoets AJ, Ylä-Herttuala S. 2009. Nrf2-dependent and-independent responses to nitro-fatty acids in human endothelial cells identification of heat shock response as the major pathway activated by nitro-oleic acid. *Journal of Biological Chemistry* **284**(48): 33233-33241.

25-Aug-2024

Prof. Dr. Juan B Barroso
Group of Biochemistry and Cell Signalling in Nitric Oxide, University Institute for Research in Olive Groves and Olive Oils, Department of Experimental Biology, University of Jaén
Experimental Biology
Jaen, Jaen 23071
Spain

RE: Nitro-fatty acids as modulators of the germination onset through the S-nitrosothiol metabolism

Dear Dr. Barroso:

We have received reviews of your manuscript, which you may view at the end of this letter. On the basis of the advice received and my own evaluation, I believe that, pending revision, this article may ultimately be acceptable for publication in Plant Physiology.

1. Address the comments from both reviewers regarding streamlining of the figures.
2. Tone down the claims regarding the causal link between NO2-Ln and GSNOR1, as indicated in the comments of reviewer #1.

When you are ready to submit your revision, click the below link and find the red arrow under your Author Tasks heading for this revision submission.

If you want to visit this version of the manuscript, you may click the link below and then click on the folder that reads "Post Decision Manuscripts".

Link Not Available

When uploading the revised version of this article, please be sure to include as complete an answer as possible to the referees' criticisms during the first round of reviews in the "response to reviewers" section.

Plant Physiology considers a revision for up to 60 days after a manuscript has been provisionally accepted; limited extension of this timeframe is at the discretion of the Monitoring Editor. A reminder notice will be sent to you 2 weeks before the deadline, at which time you may choose to contact the editorial office to request an extension; if so, please provide a justification for your request. After the expiration of this deadline, the article would need to be submitted as a resubmission and would be handled as such.

Thank you very much for giving us an opportunity to review this work. I look forward to receiving the next version.

Editor
Plant Physiology

Want to add this revision deadline to your calendar? Click below!

-- Reviewer comments:
Reviewer #1 (Comments for the Author):

The authors have thoroughly revised the manuscript. Authors are to be commended for clarifying and providing detailed description of the methods used. While some other comments have been addressed, some still require attention. Regarding major previous "specific comments":

1. Line 61: changing "biosynthesis" by "levels" would improve text flow. Arguably, by showing evidence of S-nitrosylation and repression of GSNO reductase activity, the manuscript does not address the molecular mechanisms underlying GSNO biosynthesis, but of GSNO bioavailability/metabolism.
4. I suggest changing "total" to "input or actin" to improve the image. From the western blot shown, the difference in GSNOR1 protein levels between WT and aer mutant is not obvious (i.e input signal seems lower in mutant than WT, quantitative western blot could clarify it). If indeed there is a reduction in GSNOR1 protein levels, one could expect it to be the result of lower transcript levels and cause of lower enzymatic activity. This interpretation does not support the author's argument on the impact of NO2-Ln on GSNOR1 stability and activity. Therefore, evidence provided in Fig2 based on the aer should be interpreted with caution. Indeed, NO2-Ln treatment does not seem to have the same effect on GSNOR1 stability, as shown in Fig2D. As highlighted by the authors, NO2-Ln shows a clearer impact on GSNO reductase activity and text should reflect that.
5. Although the authors' interpretation is logical, data provided is not direct evidence of a causal link between reduction in GSNO reductase activity and higher levels of protein-SNO in NO2-Ln-treated samples. The use of gsnor1 mutant lines as suggested before could be informative. Further analysis of the protein-SNO data sets generated with gsnor1 mutant could also provide insights on the signalling mechanisms of NO2-Ln.
7. The proposed role of NO2-Ln in regulating GSNOR1 activity seems to be the main novelty in the manuscript. However, based on the data provided (see comment 8 and particularly 9) its biological function is yet unclear and deserves further investigation.

Reviewer #2 (Comments for the Author):

The authors have addressed most of my concerns and significantly improved the manuscript to support their conclusions. I would recommend the authors put some work into the making the figures to be more "publication-quality" and also streamline the text/figures to improve readability.

Minor revisions
1) The authors should check their manuscript carefully again, especially references to figures/tables.
a. Line 336, page 11, there is a reference to figure S4b, which I cannot locate so I cannot comment on the conclusion made based on that data.
2) While I understand that it may be difficult to combine data from different individuals or labs, it would be great if the figures are more consistent (fonts, format, labels and nomenclature) throughout the paper. For example, it is tough to understand the figure and understand what conditions 1-7 means without moving back and forth to the legends. In another example, figure 3, "actin "may be used in place of "total".
3) Please incorporate loading controls (supplementary figure S1) into Figure 1.
4) Additional comments to improve readability of the manuscript
a. Please check for typos in the text, I have noticed quite a few.
b. Move table 1 to supplementary
c. Significantly streamline the proteomics text in the "results section" since a lot of emphasis is also on that in the "discussion section"
5) The authors have put in significant amount of work into detailing the materials and methods (including legends) of the paper, which is great since it helps in the understanding of the data.
a. Minor point for thought. I realized the samples from three biological repeats are pooled prior to MS analysis. Depending on sample complexity, this may lead to high rates of false positives and negatives, which may or may not be why NDAP-ME and RBCL were picked out by proteomics even though their S-nitrosylation levels were reduced with Ln-NO2 treatment

We have received reviews of your manuscript, which you may view at the end of this letter. On the basis of the advice received and my own evaluation, I believe that, pending revision, this article may ultimately be acceptable for publication in Plant Physiology.

Comment:

Thank you very much for assessing our manuscript. We took all reviewer suggestions and your suggestions into consideration and made substantial improvement to the manuscript.

1. Address the comments from both reviewers regarding streamlining of the figures.

Comment:

We have carefully followed the advice of both reviewers about streamlining the figures. All the changes have been compiled into both the main and supplementary figures.

2. Tone down the claims regarding the causal link between NO2-Ln and GSNOR1, as indicated in the comments of reviewer #1.

Comment:

We agree with the comment of the reviewer #1 and we have tried to tone down those statements as the editor can check mainly on comments 4 and 5.

Reviewer #1 (Comments for the Author):

The authors have thoroughly revised the manuscript. Authors are to be commended for clarifying and providing detailed description of the methods used. While some other comments have been addressed, some still require attention. Regarding major previous "specific comments":

Comment:

Thank you very much for appreciating the effort made. We have taken the suggestions into consideration and have improved the manuscript.

1. Line 61: changing "biosynthesis" by "levels" would improve text flow. Arguably, by showing evidence of S-nitrosylation and repression of GSNO reductase activity, the manuscript does not address the molecular mechanisms underlying GSNO biosynthesis, but of GSNO bioavailability/metabolism.

Comment:

Thank you. According to the reviewer suggestion, we have changed the indicated word in the summary.

4. I suggest changing "total" to "input or actin" to improve the image. From the western blot shown, the difference in GSNOR1 protein levels between WT and aer mutant is not obvious (i.e input signal seems lower in mutant than WT, quantitative western blot could clarify it).

Comment:

Thank you. First, we have changed “total” by actin due to the protein detected as input both in figures 2b and 2d according to the suggestion of the reviewer.

Furthermore, we agree that the decrease in GSNOR1 protein level is quite faint. However, we included that picture because both the GSNOR1 transcript, protein and activity analyses were done in Arabidopsis cell suspension cultures. We have done additional analyses, and we always observed a decrease in the level of GSNOR1 protein in the *aer* mutant. As the reviewer can check on the next picture, GSNOR1 protein content is always lower in *aer* compared to WT plants (plant material in this image corresponds to 7-old-Arabidopsis seedlings).

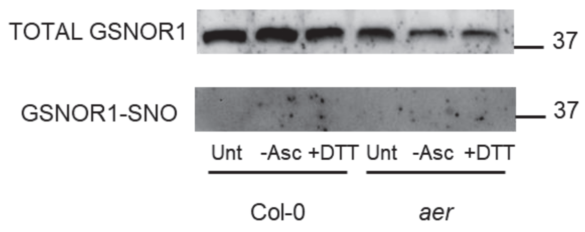

This picture depicts a biotin switch experiment where we tried (but we could not) to observe the *S*-nitrosylation of GSNOR1 in the *aer* mutant (Unt: untreated; -Asc: negative control where the -SH is not reduced by ascorbate prior the addition of Biotin-HPDP; +DTT: SNO break induced by DTT addition).

If indeed there is a reduction in GSNOR1 protein levels, one could expect it to be the result of lower transcript levels and cause of lower enzymatic activity. This interpretation does not support the author's argument on the impact of NO2-Ln on GSNOR1 stability and activity. Therefore, evidence provided in Fig2 based on the aer should be interpreted with caution.

Comment:

We really appreciate this suggestion. We agree with the reviewer that the argument about the impact of NO2-Ln on GSNOR1 stability might be lucubration. In this sense, we have modified the text on the sentences 250-261 (red colour) where we try to focus the attention on the impact of NO2-Ln on GSNOR1 bioactivity and hence on plant SNO metabolism.

Indeed, NO2-Ln treatment does not seem to have the same effect on GSNOR1 stability, as shown in Fig2D. As highlighted by the authors, NO2-Ln shows a clearer impact on GSNO reductase activity and text should reflect that.

Comment:

As the reviewer is aware of and as it has been discussed throughout the manuscript, unlike the *aer* mutant (where NO2-Ln levels are higher compared to Col-0 plants), Figure 2d uses a transgenic line with high levels of GSNOR1 (35S:Flag-GSNOR1, Col-0) in order to analyze the effect/impact of treatment with NO2-Ln on the biological activity of GSNOR1 (not to analyze the stability of GSNOR1 by NO2-Ln). Figure 2d is not intended to discuss the impact of NO2-Ln on GSNOR1 stability but to choose a system where GSNOR1 is found at higher levels (35S:Flag-GSNOR1, Col-0) to reveal the impact of NO2-Ln on its biological activity. We have tried to reflect this idea more clearly with the modification of the sentences mentioned above.

5. Although the authors' interpretation is logical, data provided is not direct evidence of a causal link between reduction in GSNO reductase activity and higher levels of protein-SNO in NO2-Ln-treated samples. The use of gsnor1 mutant lines as suggested before could be informative. Further analysis of the protein-SNO data sets generated with gsnor1 mutant could also provide insights on the signalling mechanisms of NO2-Ln.

Comment:

Thank you. We found the comment of the reviewer very interesting. However, and focusing on the relationship between GSNOR1 and NO2-Ln, we chose the genetic approach of transgenic lines overexpressing GSNOR1 (35S:Flag-GSNOR1, Col-0) instead of *loss-of-function* lines (*gsnor1*) with the aim to clearly see changes in the pattern of accumulation of SNOs. The *gsnor1 loss-of-function* line exhibit high levels of GSNO and, indirectly, of total SNOs. This approach would not allow to clearly see a further increase of SNOs by the application of NO2-Ln and this is the reason why we chose a system with lower levels of SNOs (35S:Flag-GSNOR1, Col-0). We believe with this system we could prove that NO2-Ln modulates the levels of total SNO in a clearer way than with the use of *gsnor1* plants.

The approach suggested by the reviewer is very interesting and will be taken into account for the next experimental designs where, among other behaviours, the molecular mechanisms that support the NO2-Ln-dependent *S*-nitrosylation of the signalling targets observed in recent works will be analysed (Sánchez Vicente et al., 2024, Cell Reports, 43(4):114091. doi: 10.1016/j.celrep.2024.114091).

7. The proposed role of NO2-Ln in regulating GSNOR1 activity seems to be the main novelty in the manuscript. However, based on the data provided (see comment 8 and particularly 9) its biological function is yet unclear and deserves further investigation.

Comment:

Thank you very much for this valuable suggestion. We definitely agree that further investigation is required to deepen into the molecular insights of the role of NO2-FAs and GSNOR1 activity. Indeed, extensive research about the role of NO2-FAs in multiple biological functions still needs to be addressed. However, we offer for the first time a set of evidence showing the role of NO and *S*-nitrosylation mediated by NO2-FAs on relevant plant processes such as i.e. germination. We are just opening new avenues of research where we are just beginning to uncover new functions of NO-FAs in plant physiology.

Reviewer #2 (Comments for the Author):

The authors have addressed most of my concerns and significantly improved the manuscript to support their conclusions. I would recommend the authors put some work into the making the figures to be more "publication-quality" and also streamline the text/figures to improve readability.

Comment:

Thank you very much for appreciating the effort made. We have taken the suggestions into consideration and have improved the manuscript.

Minor revisions

1) The authors should check their manuscript carefully again, especially references to figures/tables.
a. Line 336, page 11, there is a reference to figure S4b, which I cannot locate so I cannot comment on the conclusion made based on that data.

Comment:

We apologize for the mistake. Reference is to figure S2b. We have corrected in the text.

2) While I understand that it may be difficult to combine data from different individuals or labs, it would be great if the figures are more consistent (fonts, format, labels and nomenclature) throughout the paper. For example, it is tough to understand the figure and understand what conditions 1-7 means without moving back and forth to the legends. In another example, figure 3, "actin "may be used in place of "total".

Comment:

Thank you. The reviewer is completely right. We have changed in all the figures the numbers (1-7, 1-5…) by the condition indicated. We have carefully checked this aspect throughout the main and supplemental figures to ease the understanding.

3) Please incorporate loading controls (supplementary figure S1) into Figure 1.

Comment:

Thank you. Following the recommendation of the reviewer, we have cropped a section of the picture in Figure 1 to facilitate the reader the interpretation of the data. However, we still keep the whole loading control in Figure S1b and S1c. To ease the understanding, we have highlighted into the corresponding figure legends where the reader can find the whole picture of the loading controls.

4) Additional comments to improve readability of the manuscript

a. Please check for typos in the text, I have noticed quite a few.

Comment:

Thank you. We have carefully read the manuscript and check for typos.

b. Move table 1 to supplementary

Comment:

Thank you. According to the reviewer suggestion, we have moved Table 1 to supplementary. It can be found in Table S1. Due to this change, additional supplementary tables have changed the number.

c. Significantly streamline the proteomics text in the "results section" since a lot of emphasis is also on that in the "discussion section"

Comment:

Thank you. We appreciate your suggestions and have made streamline the proteomics text in the "results section" of the text. Thus, the text included in lines 387-392 and 406-421 has been removed, since they are covered in the discussion section.

5) The authors have put in significant amount of work into detailing the materials and methods (including legends) of the paper, which is great since it helps in the understanding of the data.

a. Minor point for thought. I realized the samples from three biological repeats are pooled prior to MS analysis. Depending on sample complexity, this may lead to high rates of false positives and negatives, which may or may not be why NDAP-ME and RBCL were picked out by proteomics even though their S-nitrosylation levels were reduced with Ln-NO2 treatment

Comment:

We really appreciate the reviewer thought and we coincide that, indeed, sample complexity is high. However, the results we show on Figure 5d are illustrative of the results showed throughout the manuscript because we included relevant controls for the experiment (fatty acid alone, DTT-treated samples and two different genetic backgrounds such as *aer* and *gsnor1*) which were processed in the same way to avoid the presence of those false positives and negatives.

27-Sep-2024

Prof. Dr. Juan B Barroso
Group of Biochemistry and Cell Signalling in Nitric Oxide, University Institute for Research in Olive Groves and Olive Oils, Department of Experimental Biology, University of Jaén
Experimental Biology
Jaen, Jaen 23071
Spain

RE: Nitro-fatty acids as modulators of the germination onset through the S-nitrosothiol metabolism

Dear Dr. Barroso:

We have received reviews of your manuscript, which you may view at the end of this letter. On the basis of the advice received and my own evaluation, I believe that, pending revision, this article may ultimately be acceptable for publication in Plant Physiology.

Please, specify the following points:
1. Indicate explicitly how many aer mutants were used and which material was sampled for proteomics analyses.
2. Provide controls without ascorbate, to address a comment by reviewer #2.
3. Resolve the discrepancy regarding the claim about no effect of on germination, raised by reviewer #2 regarding Fig. 4a.
4. Tone down and resolve the discussion parts pointed by reviewer #2.
5. Streamline the entire manuscript to improve flow and resolve fragmented structures, in line with the suggestions from both reviewers.

When you are ready to submit your revision, click the below link and find the red arrow under your Author Tasks heading for this revision submission.

If you want to visit this version of the manuscript, you may click the link below and then click on the folder that reads "Post Decision Manuscripts".

Link Not Available

When uploading the revised version of this article, please be sure to include as complete an answer as possible to the referees' criticisms during the first round of reviews in the "response to reviewers" section.

Plant Physiology considers a revision for up to 60 days after a manuscript has been provisionally accepted; limited extension of this timeframe is at the discretion of the Monitoring Editor. A reminder notice will be sent to you 2 weeks before the deadline, at which time you may choose to contact the editorial office to request an extension; if so, please provide a justification for your request. After the expiration of this deadline, the article would need to be submitted as a resubmission and would be handled as such.

Thank you very much for giving us an opportunity to review this work. I look forward to receiving the next version.

Sincerely,

Editor
Plant Physiology

Want to add this revision deadline to your calendar? Click below!

--- Reviewer comments:

Reviewer #2 (Comments for the Author):

I have no more major comments on the manuscript. That said, I highly recommend the authors further streamlining the manuscript text (especially long sentences with unnecessary words and fragmented structures i.e. multiple commas) to improve readability.

Reviewer #3 (Comments for the Author):

The investigation described in this manuscript aims to improve our understanding of the functions of nitro-fatty acids on plant metabolism. It continues a well-stablished research line in the group that has already generated a number of remarkable publications. The results presented here are interesting and allow the authors to establish a link between nitro-fatty acids, NO signaling through S-nitrosylation of key enzymes and transcription factors, and essential physiological processes such as seed germination.
I have de following comments:
1. In my opinion, a control without ascorbate should be included in figures 1b, 1d,3c, and 5d. The specificity of the BST for protein-SNOs is given by the incubation or not with ascorbate because it specifically converts SNOs to free thiols. The control with DTT is not good enough because it is not specific.

2. It is no clear to me how many aer mutant lines were used in the experiments shown in figs. 1-4. The authors refer to a previous article, but in my opinion this important information (number of lines, presence of other insertions...) should be included in the manuscript. Looking at the figures it seems to me that only one mutant line was used.

3. In Fig 4a it is shown that the germination rate is not affected by treatment with 1 mM GSNO in col-0. But looking at Fig. S2, GSNO treatment increases significantly the germination rate. Why this discrepancy?

4. Reading the results section, it is not clear with which material the proteomic analysis was carried out. One has to reach the Discussion (L586) or the M&M section to obtain this information. Out of curiosity, why the proteomic analysis was not performed comparing WT seedlings and aer mutants?

5. When the authors say that "regarding the molecular functions, both binding, catalytic and transcription regulator activities were over-represented (Figure 5b)", they mean in relation to proteins with these functions in the whole proteome? Any statistical analysis to prove it?

6. In their Discussion the authors state: "Thus, we evidenced the increase of total SNO by nitro linolenic acid concomitant to a decrease on GSNO reductase activity, suggesting NO released from this nitro-fatty acid impacts GSNOR stability". And again later (line 494): "Our results highlight that NO2-Ln, under certain circumstances, can release NO targeting GSNOR for S-nitrosylation and putatively to degradation, reinforcing the idea that NO mediates a feedback control over its own signaling pathway". But the authors do not present any evidence that supports an effect of NO on GSNOR stability. On the contrary, data in Fig 2d indicate that the loss of GSNOR activity mediated by NO2-Ln is not related to a decrease in GSNOR protein content. Also, in my opinion this statement "These results show that NO2-Ln is mediating the SNO modification of client proteins to not only regulate protein activity directly, but also by selectively priming proteins for ubiquitin-mediated degradation" should be toned down.

7. In the Discussion (line 579) the authors explain the absence of S-nitrosylated GSNOR in their proteomic analysis by the fact that GSNOR is an important player during stress responses and the application of NO2-Ln does not trigger a stressful situation. But this is at odds with the main conclusion of the manuscript, that NO2-Ln releases NO increasing GSNO, GSNOR-SNO and total protein-SNOs, this affecting processes such as seed germination. The model presented in Fig. 6 is only valid under stress conditions?
Minor
L110. It should read "by breaking it down",
L130. Please rephrase this sentence "In this respect, endogenous occurrence of nitro-fatty acids (NO2-FAs) generated from the interaction of unsaturated fatty acids and NO derived species, have emerged as novel molecules with relevant functions in plants".
L250. In my opinion this sentence in the Results section "In this line, previous findings have shown that NO2-FAs can mediate an electrophilic modification of their targets (i.e. nitroalkylation of members of the heat shock response, HSR) altering the capacity to bind to promoter regions of the heat shock binding elements (HSE)" should be eliminated or moved to de Discussion.
L346. I do not quite understand what the authors mean when they say: "this ABI5 accumulation was based on a dimeric structure as observed in the immunoblot under non-reducing conditions". Why the reduction of ABI5 protein levels in the aer mutants is dependent on its dimeric structure?
L433. The authors use the term "nitrogen monoxide" instead of the far more common, and also used in this manuscript, "nitric oxide".
L448. To me this sentence sounds weird: "To tackle a more in vivo tactic". Experiments can be carried out either in vitro or in vivo, but "more in vivo?".

L475: I do not understand what the authors mean in this sentence: "Similarly, NO2-Ln might be mediating a conformational change affecting to the binding of GSNOR1 to the corresponding regulatory regions, therefore impacting on gene expression". Which regulatory regions? Impacting on the expression of which genes?
L571: Please rephrase this sentence "We cannot rule out in which extent the NO2-Ln might be interfering with GSNOR1 stability" by por example "We cannot determine in which extent the NO2-Ln might be interfering with GSNOR1 stability".
L661, L749. Please provide centrifugation speed as "g".
Throughout the manuscript the authors use interchangeably the terms GSNOR and GSNO reductase. Please use just one of them.

We have received reviews of your manuscript, which you may view at the end of this letter. On the basis of the advice received and my own evaluation, I believe that, pending revision, this article may ultimately be acceptable for publication in Plant Physiology.

Comment

Thank you very much for assessing our manuscript. We took all reviewer suggestions and your suggestions into consideration and made substantial improvement to the manuscript.

Please, specify the following points:

1. Indicate explicitly how many aer mutants were used and which material was sampled for proteomics analyses.

Comment

We have clarified in material in methods the use of only one *aer* mutant line with a T-DNA insertion in the region promoter obtained from Arabidopsis Biological Resource Center.

We have included the information on the sample used for proteomic analyses not only in material and methods but also in the results to facilitate the understanding to the reader.

2. Provide controls without ascorbate, to address a comment by reviewer #2.

Comment

Following the reviewer suggestion, we have included a new supplemental figure (Fig. S4) in material and methods, in which we show that in our experimental conditions the sensitivity of control without ascorbate is quite similar to the use of DTT, so that confirming the results of BST obtained in this work.

3. Resolve the discrepancy regarding the claim about no effect of on germination, raised by reviewer #2 regarding Fig.4a.

Comment

We apologize since this apparent discrepancy is a mistake in the construction of the figure. The germination percentage after GSNO treatment in Fig 4a is really significant.

4. Tone down and resolve the discussion parts pointed by reviewer #2.

Comment

We have carefully revised these points trying to reflect our results in a more clear and precise manner.

5. Streamline the entire manuscript to improve flow and resolve fragmented structures, in line with the suggestions from both reviewers.

Comment

We have carefully revised the manuscript to improve its reading following the indication of the reviewers. We have sent the manuscript to a native English speaker who has carefully revised it to improve its readability with special attention on the reviewer's suggestions about streamlining the manuscript text.

Reviewer 2:

I have no more major comments on the manuscript. That said, I highly recommend the authors further streamlining the manuscript text (especially long sentences with unnecessary words and fragmented structures i.e. multiple commas) to improve readability.

Comment

Thank you very much for your contribution to improve the manuscript. We have sent the manuscript to a native English speaker who has carefully revised it to improve its readability with special attention on the reviewer's suggestions about streamlining the manuscript text.

Reviewer 3:

1. In my opinion, a control without ascorbate should be included in figures 1b, 1d,3c, and 5d. The specificity of the BST for protein-SNOs is given by the incubation or not with ascorbate because it specifically converts SNOs to free thiols. The control with DTT is not good enough because it is not specific.

Comment

Thank you very much four your comments. Throughout the manuscript we have used both controls, Asc removal and addition of DTT (Figures 1d, 3b, 3c, 4d and 5d) showing a similar specificity since in both conditions there is a comparable reduction of the S-nitrosylation pattern after the treatment with NO_2-Ln. The plant material for the S-nitrosylation assays were plant extracts. For this reason, we have carried out a new assay, similar to that in Figure 1d, using plant extracts and both negative controls -Asc and +DTT in order to confirm that the results of this manuscript are consistent. This result has been included as supplemental figure 4 in methods section. In this figure, we use the addition of DTT that reduces the formation of SNOs after NO2-Ln treatment, consistent with the presence of a reversible thiol modification. The biotin switch was assayed without ascorbate, to assure that the immune reactivity of the protein was ascorbate dependent (Supplementary Fig. 4a) and to prevent switching SNO for biotin. Furthermore, the use of DTT as the negative control in NO2-Ln-mediated S-nitrosylation studies has been shown in a recent publication (Sánchez-Vicente et al., 2024) in addition to other S-nitrosylation studies (Albertos et al., 2015; Niu et al: 2019)

The changes have been included in the text manuscript in L731-739 in methods section.

References:

-Sánchez-Vicente et al., 2024. Reversible S-nitrosylation of bZIP67 by peroxiredoxin IIE activity and nitro-fatty acids regulates the plant lipid profile. Cell Rep 43(4):114091.

-Albertos et al., 2015. S-nitrosylation triggers ABI5 degradation to promote seed germination and seedling growth. Nature communications, 6:8669.

-Niu et al., 2019. Proteomic investigation of S-nitrosylated proteins during NO-induced adventitious rooting of Cucumber. IJMS 20: 5363.

2. It is no clear to me how many aer mutant lines were used in the experiments shown in figs. 1-4. The authors refer to a previous article, but in my opinion this important information (number of lines, presence of other insertions...) should be included in the manuscript. Looking at the figures it seems to me that only one mutant line was used.

Comment

Thank you for your suggestion. We have used only one *aer* mutant line that shows and increase in NO_2-FAs and GSNO levels as described in the previous article cited (Mata-Pérez et al., 2020). This mutant is a homozygous line with a T-DNA insertion in the promoter region that was obtained from Arabidopsis Biological Resource Center. To clarify this, we have rephrased the sentences on L595-598 in methods section.

Reference:

Mata-Pérez et al., 2020. Endogenous biosynthesis of S-nitrosoglutathione from nitro-fatty acids in plants. Front Plant Sci., 962.

3. In Fig 4a it is shown that the germination rate is not affected by treatment with 1 mM GSNO in col-0. But looking atFig. S2, GSNO treatment increases significantly the germination rate. Why this discrepancy?

Comment

Thank you very much for your appreciation. As specified in the manuscript, NO is related to seed dormancy and germination and therefore GSNO, as a NO donor, usually improves seed germination. This apparent discrepancy is a mistake in the construction of the figure 4a in which the increase of germination observed after GSNO treatment is really statistically significant. Accordingly, we have inserted the statistical significance on GSNO treatment in comparison to Col0 in the figure 4a (*<0.05)

4. Reading the results section, it is not clear with which material the proteomic analysis was carried out. One has to reach the Discussion (L586) or the M&M section to obtain this information.

Comment

Thank you for this comment. For proteomic analysis we used Arabidopsis cell suspension cultures treated with methanol, Ln or NO_2-Ln. The protein extract obtained after these treatments were subsequently processed to perform LC-MS/MS as specified in Methods

section. We have added this information on L361-L364 in the result section to facilitate its understanding to the readers.

Out of curiosity, why the proteomic analysis was not performed comparing WT seedlings and aer mutants?

Comment

Thank you for this interesting suggestion. We have no performed the proteomic analysis using *aer* mutants because we were interested in analysing the specific S-nitrosylating capacity of NO_2-Ln comparing with other treatments used as controls such as Ln. However, the reviewer's suggestion is important to determine endogenous S-nitrosylation mediated by NO_2-FAs that will be considered in future investigations.

5. When the authors say that "regarding the molecular functions, both binding, catalytic and transcription regulator activities were over-represented (Figure 5b)", they mean in relation to proteins with these functions in the whole proteome? Any statistical analysis to prove it?

Comment

Supplemental table 1 contains the list of proteins that were exclusively S-nitrosylated by NO_2-Ln treatment, after discarding the S-nitrosylated proteins appearing in the respective methanol and linolenic acid controls. This set of S-nitrosylated proteins by NO_2-Ln was compared with the complete *Arabidopsis thaliana* proteome by PANTHER Overrepresentation Test to identify terms enriched by GO ontology. Statistical significance of the study was calculated by Fisher's exact test and Bonferroni correction for multiple testing to select GO terms with adjusted p values < 0.05. In the "Bioinformatics" section of materials and methods (L796-L798) of the manuscript, the enrichment analysis performed by the PANTHER Overrepresentation Test has been described in more detail.

6. In their Discussion the authors state: "Thus, we evidenced the increase of total SNO by nitro linolenic acid concomitant to a decrease on GSNO reductase activity, suggesting NO released from this nitro-fatty acid impacts GSNOR stability". And again later (line 494): "Our results highlight that NO2-Ln, under certain circumstances, can release NO targeting GSNOR for S-nitrosylation and putatively to degradation, reinforcing the idea that NO mediates a feedback control over its own signaling pathway". But the authors do not present any evidence that supports an effect of NO on GSNOR stability. On the contrary, data in Fig 2d indicate that the loss of GSNOR activity mediated by NO2-Ln is not related to a decrease in GSNOR protein content.

Comment

We really appreciate this suggestion. We agree with the reviewer that the argument about the impact of NO_2-Ln on GSNOR stability is only a possibility and it is not demonstrated in our manuscript. Indeed, Figure 2d is not intended to discuss the impact of NO_2-Ln on GSNOR1 stability but to choose a genetic background with increased levels

of GSNOR to reveal the impact of NO_2-Ln on its biological activity. We have tried to reflect this idea more clearly with the modifications made in L471, L472 and L482 where “stability” has been replaced by “(bio)activity/function”.

Also, in my opinion this statement "These results show that NO2-Ln is mediating the SNO modification of client proteins to not only regulate protein activity directly, but also by selectively priming proteins for ubiquitin-mediated degradation" should be toned down.

Comment

This sentence has been rephrased (L548-550) in order to show that NO_2-Ln-mediated S-nitrosylation might be involved in the regulation of protein ubiquitination since different ubiquitin-related proteins has been described as NO_2-Ln targets in our results.

7. In the Discussion (line 579) the authors explain the absence of S-nitrosylated GSNOR in their proteomic analysis by the fact that GSNOR is an important player during stress responses and the application of NO2-Ln does not trigger a stressful situation. But this is at odds with the main conclusion of the manuscript, that NO2-Ln releases NO increasing GSNO, GSNOR-SNO and total protein-SNOs, this affecting processes such as seed germination. The model presented in Fig. 6 is only valid under stress conditions?

Comment

Thank you for this appreciation. We apologize because this sentence was not well-expressed. Reference to stress situations has been removed because we only wanted to highlight that during these stress situations usually increase NO_2-FAs (Mata-Pérez et al., 2016) and total SNOs. This part has been modified (L560-564) in order to clarify that in our conditions the S-nitrosylation of GSNOR after ACSC treatment with NO_2-Ln could affect its proteomic detection taking into account previous publications.

For this reason, figure 6 is a general model not only a specific one for stress conditions.

References:

-Mata-Pérez et al., 2016. Nitro-fatty acids in plant signalling: nitro-linolenic acid induces the molecular chaperone network in Arabidopsis. Plant Physiol. 170(2): 686-701.

Minor

L110. It should read "by breaking it down"

Comment

Thank you for this observation. It has been modified L115

L130. Please rephrase this sentence "In this respect, endogenous occurrence of nitro-fatty acids (NO2-FAs) generated from the interaction of unsaturated fatty acids and NO derived species, have emerged as novel molecules with relevant functions in plants".

Comment

This sentence has been modified in L134-L136.

L250. In my opinion this sentence in the Results section "In this line, previous findings have shown that NO2-FAs can mediate an electrophilic modification of their targets (i.e. nitroalkylation of members of the heat shock response, HSR) altering the capacity to bind to promoter regions of the heat shock binding elements (HSE)" should be eliminated or moved to de Discussion.

Comment

Thank you for this comment. The sentence has been moved to the Discussion section (L540-L543).

L346. I do not quite understand what the authors mean when they say: "this ABI5 accumulation was based on a dimeric structure as observed in the immunoblot under non-reducing conditions". Why the reduction of ABI5 protein levels in the aer mutants is dependent on its dimeric structure?

Comment

Thank you for this appreciation. We apologize because this sentence was not well-expressed. Following the reviewer's comment, this sentence has been modified (L334-336). The accumulation of ABI5 does not depend on the dimeric structure. We wanted to show that the detection of ABI5 S-nitrosylation is carried out on the form of functional homodimer under non-reducing conditions (Nakamura et al., 2001; Albertos et al., 2015).

References:
-Nakamura et al., 2001. Physical interactions between ABA response loci of Arabidopsis. Plant J, 26: 627-635
-Albertos et al., 2015. S-nitrosylation triggers ABI5 degradation to promote seed germination and seedling growth. Nature communications, 6:8669.

L433. The authors use the term "nitrogen monoxide" instead of the far more common, and also used in this manuscript,"nitric oxide".

Comment

This change has been made in L421.

L448. To me this sentence sounds weird: "To tackle a more in vivo tactic". Experiments can be carried out either in vitro or in vivo, but "more in vivo?".

Comment

Thank you, we agree with this comment. The sentence has been modified, L435.

L475: I do not understand what the authors mean in this sentence: "Similarly, NO2-Ln might be mediating a conformational change affecting to the binding of GSNOR1 to the corresponding regulatory regions, therefore impacting on gene expression". Which regulatory regions? Impacting on the expression of which genes?

Comment

Thank you for this observation. We have modified the sentence (L461-462) to show the possibility that NO_2-Ln mediated effect on TFs that bind to GSNOR promoter could be the responsible for the reduction in GSNOR1 expression.

L571: Please rephrase this sentence "We cannot rule out in which extent the NO2-Ln might be interfering with GSNOR1stability" by por example "We cannot determine in which extent the NO2-Ln might be interfering with GSNOR1 stability".

Comment

This change has been addressed in L561.

L661, L749. Please provide centrifugation speed as "g".

Comment

It has been properly amended in L 642 and L 757

Throughout the manuscript the authors use interchangeably the terms GSNOR and GSNO reductase. Please use just one of them.

Comment

Thank you for this observation. We have revised the manuscript to use mainly GSNOR.

27-Nov-2024
Prof. Dr. Juan B Barroso
Group of Biochemistry and Cell Signalling in Nitric Oxide, University Institute for Research in Olive Groves and Olive Oils, Department of Experimental Biology, University of Jaén
Experimental Biology
Jaen, Jaen 23071
Spain

MSID: PP2024-RA-02192DR2
MS TITLE: Nitro-fatty acids as modulators of germination onset through S-nitrosothiol metabolism

Dear Dr. Juan Barroso:

I am pleased to inform you that your revised manuscript "Nitro-fatty acids as modulators of germination onset through S-nitrosothiol metabolism" has been accepted for publication in Plant Physiology. Your manuscript will be checked for consistency with journal formatting and image standards by one of our Science Editors, who will contact you shortly. They may have a number of queries and suggestions for you. Please keep in mind that they work to increase the online visibility of your article and its attraction for readers from the widest of backgrounds. You will have the opportunity to review any revisions, and I hope you will work constructively with them to get the most out of your research. You will also have the opportunity to review all changes when you receive the author proofs for your article.

Once validated for publication, your article will be sent to a Science Editor for a final review of your manuscript and source files. After your final files are uploaded, your article will appear in the next available issue and the online Preview version of your article will be posted after your signed Copyright and Checklist forms and final source files are received.

Please be sure to include the following in your manuscript file:
Harvard style citations
List of all author contributions and funding information at the beginning of manuscript after the titles and author list.
List all supplemental materials associated with the article, with titles, at the end of the ms, before the references and acknowledgments section.
Complete Figure Legends for all figures appearing in the article listed before the Literature Cited.

LICENSE INFORMATION Shortly after your paper is sent to production, you will receive an email from our publisher's author support team, **SciPris**, with information on article processing charges, as well as the types of Open Access license you would be able to purchase. The accepted version of your manuscript will not appear on our Advance Articles page until the license is selected and signed, so please look out for an email from SciPris. For more information see the Article Publication Fees section of our author guidelines: https://academic.oup.com/plcell/pages/General-Instructions

Finally, we encourage your submission of artwork for the journal cover. If there is an image or illustration related to your paper that you would like to have considered, please email it in .pdf or .tif format to clowe@aspb.org. An image title, brief, 2-3 sentence description of the image, and the name of the person credited for the image are also encouraged. If your image is selected to appear on the online cover of the journal, you will be contacted with the scheduled issue date and further information.

Thank you and congratulations on your Plant Physiology paper!

Sincerely,

Editor
Plant Physiology

=====
FIRST AUTHOR PROFILES
=====

We recognize the hard work that goes into being First Author of a Plant Physiology paper by publishing a "First Author Feature" for each First Author alongside your paper.

If the first author(s) would like to submit information for a feature on Facebook and Twitter, fill out the form by clicking on the link below:

Plant Physiology First Author Feature Form: https://forms.office.com/r/ZBCGZfhm8F
If you cannot access the form through the link above, please contact Rachel at rbelsky@aspb.org.

To upload an image of yourself to accompany your profile, please put your photo in the correct journal folder at the link below. You must label your image: Author First Name Last Name Article Title.

First Author Profile Image Dropbox: https://www.dropbox.com/scl/fo/xz029p7qjr2jml2rp2kmq/h?dl=0&rlkey=6fkxcaqmxx35ap152b2osy1fz

=====
IMPORTANT REMINDER: PEER REVIEW REPORTS
=====

If you opted to publish a peer review report along with your article during the original submission process, it will be prepared by the editorial staff and publicly posted with your manuscript, inside the zip file that contains any other supplemental material. As a reminder, the peer review report is a public record of all comments from editors and reviewers, as well as your prior responses, as you received them in the decision letters for each draft of your manuscript. If you agreed to publish this report and have changed your mind, or are not sure if you selected this option, please contact the editorial office as soon as possible before signing the license agreement from our publisher.

-- Reviewer comments:
